# Thyme, Oregano, and Cinnamon Essential Oils: Investigating Their Molecular Mechanism of Action for the Treatment of Bacteria-Induced Cystitis

**DOI:** 10.1021/acsomega.5c10256

**Published:** 2026-01-12

**Authors:** Emanuele Carosati, Laura Beatrice Mattioli, Alberto Santini, Giovanni Caprioli, Matteo Micucci, Gianmarco Mangiaterra, Carla Marzetti, Maria Scola Gagliardi, Franks Kamgang Nzekoue, Sauro Vittori, Giovanni Scala, Michele Ceccarelli, Maria Frosini, Ivan Corazza, Roberta Budriesi

**Affiliations:** † Chemical and Pharmaceutical Sciences Department, 9315 University of Trieste, Trieste 34127, Italy; ‡ Pharmacy and Biotechnology Department, 9296 Alma Mater Studiorum-University of Bologna, Bologna 40126, Italy; § Valsambro S.r.l., Bologna 40121, Italy; ∥ School of Pharmacy, 9313 University of Camerino, Camerino 62032, Italy; ⊥ Biomolecular Sciences Department, University of Urbino “Carlo Bo”, Urbino 61029, Italy; # UniCamillus—Saint Camillus International University of Health Sciences, Rome 00131, Italy; ¶ Biology Department, 9307 University of Naples “Federico II”, Naples 80138, Italy; ∇ Electrical Engineering and Information Technology Department, University of Naples “Federico II”, Naples 80138, Italy; ○ BIOGEM Institute of Molecular Biology and Genetics, Ariano Irpino 83031, Italy; ⧫ Life Sciences Department, University of Siena, Siena 53100, Italy; †† Medical and Surgical Sciences Department, Alma Mater Studiorum-University of Bologna, Bologna 40126, Italy

## Abstract

Pathogen infections, exacerbated by emerging drug resistance, remain among the most challenging health issues, for which multitargeting approaches may offer effective solutions. In this context, medicinal plants, including essential oils, provide complex mixtures of diverse molecules that can exert therapeutic effects, either alone or synergistically with established antibiotics. Although several databases comprehensively collect information on the antibacterial properties of medicinal plants, including chemical composition, bioactivity data, and ethnobotanical uses, there is a notable lack of tools to hypothesize mechanisms of action. To address this gap, we developed a computational pipeline that integrates chemoinformatics and bioinformatics, specifically designed for scenarios in which only the chemical composition of a complex mixture of natural phytocompounds is available. Beginning with an ultralarge, structure-based screening across thousands of proteins and their potential binding sites of six bacterial species, we used the predicted targets as input for bioinformatics tools commonly employed in the omics fields, such as pathway enrichment analysis and network analysis. Using this pipeline, we modeled how the essential oils of thyme, oregano, and cinnamon exert antibacterial activity against six bacterial pathogens. Applied here in the context of urinary tract infection, but extendable to other therapeutic scenarios, this pipeline provides a novel protocol for mode of action investigation and experimental prioritization, to be applied in drug discovery involving natural substances.

## Graphical abstract

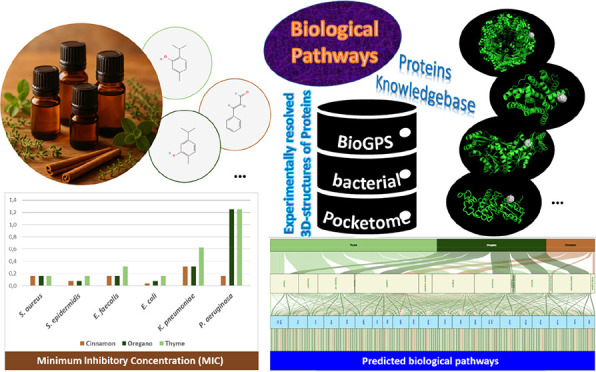

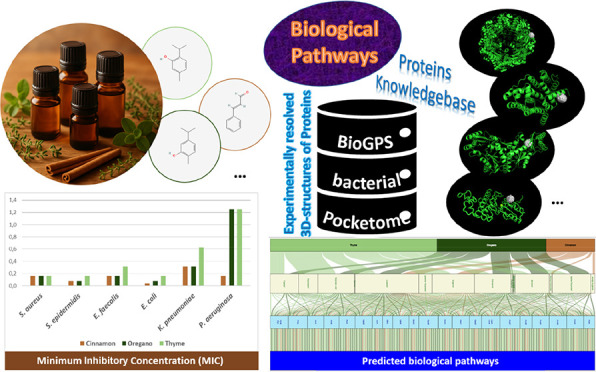

## Introduction

Humans have been using medicinal plants as curative remedies for millennia, and traditional medicine is encouraged by the World Health Organization (WHO) as a means to improve public health^1,2^ by helping address currently unmet health needs.^3^ Many of the most challenging health issues involve complex and multifactorial diseases, such as cancer, syndromes, neurodegenerative disorders, and infectious diseases;^4^ in these cases, multitargeting strategies and network pharmacology may offer effective solutions.^5^ Natural products (NP) from medicinal plants could contribute significantly, through the complementary and potentially synergistic actions of their different molecular components.^6^

Significant progress in chemical characterization of medicinal plants over the recent decades^7^ spans several aspects, including advances in separation and quantification techniques, which have enabled highly sensitive molecular identification, and curated databases, which catalogue natural compounds linking them to biological activities and (less frequently) to compositional data. These databases vary in scope and focus, reflecting the diversity of NPs and their origins; some emphasize molecular properties while others document ethnobotanical uses.^3,8–16^ However, there is a lack of predictive tools specifically dedicated to explore the therapeutic potential of phytocomplexes, which are complete, chemically diverse ensembles of bioactive (and nonbioactive) constituents naturally present in plant extracts or essential oils. Developing such tools could enable the identification of new uses for known substances and ultimately extend their therapeutic potential.^17,18^

This challenge falls within the domain of chemoinformatics, where the term “target fishing” refers to computational methods that generate lists of protein targets for a given molecule.^19–21^ For ligand-based methods concerns arise regarding applicability to NPs because the underlying predictive models are typically trained on drug-like chemical space. Structure-based approaches, in contrast, rely solely on the availability of 3D structural data for the protein targets, which can severely limit the applicability, although recent advances in protein structure prediction^22,23^ have dramatically changed the scenario, giving a new boost to structure-based tools.

Several virtual screening methods exist for target fishing.^24^ Among others, BioGPS^25^ evaluates the numerical “complementarity” between a molecule and a set of protein pockets, organized in a database and characterized by GRID-derived Molecular Interaction Fields (MIF).^26^ Traditionally, BioGPS is used to profile a single molecule by comparing its calculated complementarity scores across protein pockets;^27^ when applied to all molecules within a phytocomplex, these profiles can be merged and integrated with biological information linking the protein-gene-pathway-disease cascade, providing a foundation for bioinformatic interpretation of how the phytocomplex can modulate one or more biological pathways.

In this project, we focused on one of the most widespread infections globally, affecting individuals of all ages and background.^28^ Urinary Tract Infection (UTI), commonly known as cystitis, are caused by both Gram-negative and Gram-positive bacteria and are classified as either uncomplicated (not associated with treatment failure or poor outcomes) or complicated (associated with a higher risk of treatment failure). UTIs represent a significant public health concern, further exacerbated by the rise of multidrug-resistant strains.^28^ Despite considerable progress in prevention and treatment of UTIs, additional research is needed, and NPs may offer promising alternatives.

In the field of NPs, essential oils (EOs) are volatile, hydrophobic mixtures of small aliphatic and aromatic compounds obtained primarily by distillation (in contrast, plant extracts are broader, nonvolatile preparations obtained with solvents that capture a wider range of polar and nonpolar phytochemicals). EOs are particularly relevant due to their therapeutic potential; these lipophilic mixtures often exhibit a broad spectrum of biological activities,^29,30^ including antibacterial activity,^31^ which are important for preventing and controlling pathogenic bacterial growth in both localized^32^ and systemic infections.^30–32^ Notably, the EOs of thyme, oregano, and cinnamon have shown beneficial effects in cystitis attributable to their antimicrobial and anti-inflammatory properties.^33^

Here, we present experimental data on the antibacterial activity of thyme, oregano, and cinnamon EOs against the most common bacterial pathogens responsible for cystitis,^28^ along with a new computational pipeline capable of identifying putative targets for these phytocomplexes and the biological pathways enriched in targets predicted to interact with one or more constituent molecules. By integrating experimental results with computational predictions, we investigated the Mechanism-of-Action (MoA) of the three EOs. This pipeline can be incorporated into discovery projects involving phytocomplexes, helping to prioritize hypotheses and guide experimental validation.

## Materials and Methods

### Essential Oils

#### Overview

Three essential oils, namely *Cinnamomum zeylanicum* Nees from nees bark, *Origanum vulgare* L. from the flowering tops, and *Thymus vulgaris* L. from the flowering tops, were selected due to their traditional use in the treatment of cystitis, although their molecular mechanisms of action remain unknown.^33^ The oils were supplied by BIO-LOGICA S.r.l. (Bologna, Italy), and their experimental characterization included analyses of chemical composition and antibacterial activity.

#### Chemical Characterization: Chemical Composition

The determination of EO chemical composition was carried out using gas chromatography–mass spectrometry (GC–MS) using an Agilent 7890B gas chromatograph equipped with an autosampler (PAL RSI 85) and coupled to a 5977B single quadrupole mass spectrometer (Santa Clara, California, USA). For each EO, after dilution 1:2000 in *n*-hexane (Carlo Erba, Milan, Italy), 1 μL of the diluted sample was injected in the front inlet set at 280 °C.^34^ Injection was performed in split mode (1:100) with a split flow of 120 mL/min using an Agilent 5190-3983 liner (800 μL). A HP-5MS capillary column (30 m × 0.25 mm i.d. × 0.25 μm film thickness, 5% phenylmethylpolysiloxane) was used for separation (Agilent, Folsom, CA, USA), and Helium was used as carrier gas with a flow rate of 1.2 mL/min. The oven temperature was set as follows: 60 °C for 5 min, followed by 4 °C/min up to 160 °C, then 11 °C/min up to 280 °C with a hold time of 15 min, and finally 15 °C/min until 300 °C for a total run time of 57.74 min. MSD transfer line temperature was set at 300 °C. Analysis was made in electron impact (EI) mode (internal ionization source; 70 eV) with a scan range from 29 to 400 *m*/*z*, after a solvent delay of 2.5 min. Compounds were identified by two approaches: (i) comparing the RI reported in libraries^35–37^ with the obtained RI, calculated from a mix of *n*-alkanes (C8–C20 supplied by Supelco, Bellefonte, Pennsylvania, US); (ii) comparing the obtained mass spectra with libraries,^35–38^ and available analytical standards, according to known methods.^39^ For each compound studied, we report the data observed on the three EOs.

#### Biological Characterization: Antibacterial Activity

The antibacterial activity of the essential oils was evaluated against both Gram-positive and Gram-negative bacterial strains (Table 1: ATCC main identifiers). Reference strains were grown on Mueller Hinton (MH) agar plates and stored in MH broth supplemented with 15% glycerol at −80 °C. All culture media were obtained from Oxoid (Thermo Fisher Scientific, Waltham, MA, USA).

**Table 1:** Details for the BioGPS Pocketomes: Number of Pockets, PDB Entries, and UniProt Codes; Additional Data Include the Number of UniProt Codes Obtained When Collectively Considering all the KEGG Entries for the Same Bacterium, and the Number of UniProt Codes as Available from Genomes on the ATCC Website

		Gram+			Gram–		
		*Staphylococcus aureus*	*Staphylococcus epidermidis*	*Enterococcus faecalis*	*Escherichia coli*	*Klebsiella pneumoniae*	*Pseudomonas aeruginosa*
ATCC	Main identifier^a^	29213	RP62A (35984)	29212	25922	700603	27853
BioGPS Pocketomes	Nr Pockets^b^	8615	269	1579	55160	2932	13604
	Nr PDB entries	1623	56	235	6733	555	1773
	Nr Uniprot	611	30	142	2240	172	732
KEGG	Main identifier (T number, org code)^c^	T00225, sac	T00229, ser	T03320, efq	T00007, eco	T00566, kpn	T00035, pae
	Additional identifiers^d^	28	2	3	37	14	4
UniProt	Organism identifiers^e^	**STAAU**, STAAC, STAAE, STAAN, STAAM, STAAR, STAAS, STAAW, STAA1, STAAB, STAA3, STAA8	**STAEP**, STAES, STAEQ	**ENTFL**, ENTFA	**ECOLX**, ECOLI, ECOL6, ECOLC, ECOBD, ECOCB, ECODH, ECOH1	**KLEPN**	**PSEAI**, PSEA8, PSEA7, PSEAB
	Nr UniProt^e^	12759 (**1344**)	2204 (**1245**)	1338 (**1209**)	33981 (**3121**)	13738 (**3072**)	6180 (**2375**)
	Nr UniProt in common with BioGPS Pocketome^e^	319 **(66)**	13 **(3)**	34 **(4)**	1334 **(115)**	21 **(21)**	25 **(16)**
	Bacterial genome coverage^e^	23.7% **(4.9%)**	1.0% **(0.2%)**	2.8% **(0.3%)**	42.7% **(3.7%)**	0.7% **(0.7%)**	1.1% **(0.7%)**
STRINGdb	Main identifier	93061	176279	1260356	199310	272620	208964

a All the bacterial ATCC species belong to the Microbiology strain collection of the University of Urbino (Department of Biomolecular Sciences).

b String filters like ≪% *Escherichia coli* %≫ were used when accessing the BioGPS database.

c These values are obtained from the KEGG Web site, searching the given *T* number as KEGG entry, under the field “Statistics/Number of protein genes”.

d Numbers refer to identifiers that include UniProt-pathway(s) correspondence for at least one UniProt code.

e Data referred to as the “main identifiers” are reported in bold; the entire data set refers to all identifiers, including both the “main identifiers” and the “additional identifiers”, obtained from the field “Mnemonic name”.

Preliminary antimicrobial susceptibility tests were performed using the agar well diffusion method, as previously described,^40^ with cation-adjusted Mueller Hinton (MHII) as the culture medium. Each EO was tested by applying 20 μL of a 10% solution in ethanol. Ciprofloxacin (CPX, Merck) served as the reference antimicrobial agent, while 96% ethanol was included as a control to exclude solvent-related effects.

Minimum inhibitory concentration (MIC) and minimum bactericidal concentration (MBC) values for each EO were determined against the tested bacterial strains, following CLSI guidelines.^41^ All experiments were performed in three independent biological replicates.

### Combined Chemoinformatics and Bioinformatics Analysis

#### Overview

The set of molecules listed in Table 2 (and in Supporting Information S2) underwent chemoinformatics and bioinformatics characterization to identify potential molecular targets and biological pathways. The target fishing approach was performed using the BioGPS software,^25^ with default parameters and a database of protein pockets derived from the RCSB Protein Data Bank.^42^ BioGPS identifies binding pockets using the Flapsite procedure,^43,44^ which detects both internal pockets and those located at the interface between two or more protein chains. As in other structure-based approaches,^45^ the core calculation employs GRID molecular interaction fields (MIF)^26^ to characterize both the binding pockets and the ligands.

**Table 2:** Chemical Constituents of the Three EOs, Grouped by Their Phytochemical Classes^b^

CID	compounds^a^	*Cinnamomum zeylanicum* %	*Origanum vulgare* %	*Thymus vulgaris* %
**Monoterpenoids**				
26049	3-Carene	-	-	-
7460	α-Phellandrene	0.29	-	-
6654	α-Pinene	1.73	0.13	0.48
7462	α-terpinene	-	Traces	Traces
17868	α-Thujene	Traces	Traces	-
31253	β-Myrcene	-	0.1	0.18
14896	β-Pinene	0.75	0.14	Traces
6616	Camphene	0.13	Traces	0.17
440917	d-Limonene	1.67	0.39	**19.35**
7461	γ-Terpinene	-	0.43	-
10703	*o*-Cymene	1.41	**9.54**	**10.26**
18818	Sabinene	-	-	-
79035	Tricyclene	-	-	-
**Oxygenated monoterpenoids**				
17100	α-Terpineol	Traces	0.58	2.94
64685	Borneol	-	0.75	0.20
93009(−)	Bornyl acetate	-	-	2.33
6950274(+)				
2537	Camphor	-	0.73	0.17
10364	Carvacrol	-	**72.36**	**23.23**
2758	Eucalyptol	1.87	1.38	0.53
11467	y-Terpineol	-	-	0.29
6321405	iso-Borneol	-	-	Traces
91496	Limonene oxide	-	-	Traces
6549	Linalool	**6.80**	2.17	1.13
11230	Terpinen-4-ol	-	0.39	0.10
11468	Terpineol-1	-	-	0.10
6989	Thymol	-	**8.08**	**31.08**
14104	Thymol methyl ether	-	0.18	-
14529	*p*-Cymen-8-ol	-	-	Traces
**Phenylpropanoids**				
7136	Acetyl eugenol	0.33	-	0.39
637511	Cinnamaldehyde, (E)	**54.40**	-	-
5282110	Cinnamyl acetate	0.77	-	-
3314	Eugenol	**20.55**	-	**5.97**
5144	Safrole	0.65	-	-
Sesquiterpenes				
5281520	α-Caryophyllene	0.31	-	-
5281515	Caryophyllene	6.73	0.52	0.22
1742210	Caryophyllene oxide	0.29	0.95	0.10
289151	Longifolene	-	-	0.34
**Other organic compounds**				
246728	3-Octanone	-	0.1	-
240	Benzaldehyde	0.10	-	-
2345	Benzyl benzoate	0.88	-	-
Total peaks identified (%)		99.75	98.92	99.70

a Within each class, components are listed according to their elution from an HP-5MS column (30 m × 0.25 mm i.d. × 0.25 μm film thickness, 5% phenylmethyl polysiloxane). Full data is available as Supporting Information, S1: retention time (min); RI calculated via linear retention index experimentally determined according to Van den Dool and Kratz,^39^ calculated using a mixture of *n*-alkanes (C8–C30, Supelco, Bellefonte, CA, US) as well as RI Literature, taken from the NIST 17 library.^35^ “Traces” stands for percentage <0.1%.

b The most representative compounds are reported in bold. PubChem identifiers (CID) are reported in the first column.

For each identified binding pocket, the corresponding protein was mapped to its UniProt code(s) and associated gene(s). Genome data for the bacterial species under study, as well as gene-pathway relationships, were retrieved from the KEGG database (Kyoto Encyclopedia of Genes and Genomes)^46–48^ (additional details are provided in Supporting Information, sections S5, S6, and S7).

This workflow supports a systematic navigation from molecules to pathways, following the scheme

Molecule→Pocket→PDB→UniProt→Gene→Pathway

Each UniProt entry typically corresponds to a unique gene name, and vice versa, with rare exceptios involving protein complexes. In contrast, relationships between genes and pathways are many-to-many and can be sourced from different databases; for the bacterial organisms considered here, we used the KEGG database,^47^ but for other organisms Reactome^49,50^ or WikiPathways^51,52^ may be more suitable. As pathway databases often include very large pathways containing thousands of genes, which can obscure more specific biological signals, we excluded pathways involving more than 1000 genes to reduce bias and highlight smaller, more informative pathways.

Overall, our analysis combines chemoinformatics, using BioGPS to link molecules to genes through the prediction of putative binding pockets, with bioinformatics, applying pathway enrichment analysis. Data mining procedure and manual curation ensure the statistical robustness of the results. The full pipeline is schematized in Figure 1, while the detailed methodology for each step is provided below.

**Figure 1:**
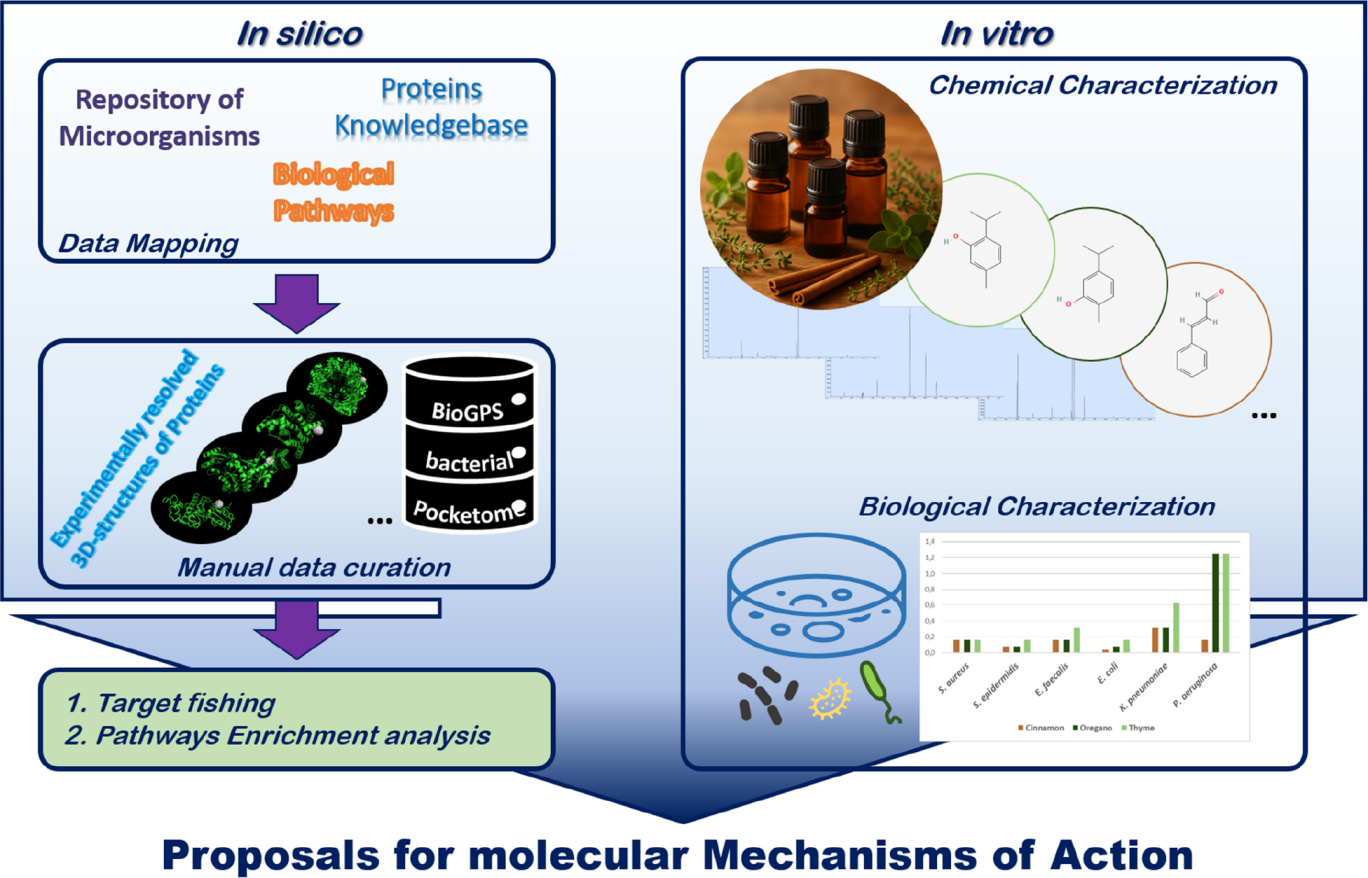
Schematic workflow summarizing the pipeline presented in this work. The schema reports the main steps to combine in silico (left panel) and in vitro (right panel) activities to investigate the phytocomplexes MoA characterization and prioritization of experiments on natural compounds.

#### The BioGPS Pocketome

The BioGPS database currently includes more than 850 K pockets, of which approximately 23.5% originate from *Homo sapiens* (about 200 K entries); in contrast, bacterial pockets are less represented, with substantial variability across species. Any data set of pockets retrieved from BioGPS is referred to as “pocketome”. In this study, pocketomes for the six bacterial species were obtained by querying the BioGPS database using “unstrict criteria”; therefore, the corresponding data extracted from KEGG and UniProt databases through several bacterial identifiers were categorized as either “main”, corresponding to the ATCC studied experimentally, or “additional” (details are given in Table 1).

A key aspect of this analysis was to estimate bacterial genome coverage, defined as the percentage of bacterial proteins represented in the constructed pocketome relative to the full genome of each organism. Using a strict approach (limited only to the main ATCC strains) coverage was extremely low, reaching approximately 5% for only two species, *Staphylococcus aureus* and *Escherichia coli*. This limitation motivated the development of a broader data-mining and manual curation strategy, described above. By incorporating additional strains, we were able to increase the number of UniProt entries and, after converting these to the corresponding gene names, we achieved substantially improved coverage for *E. coli* (42.7%) and *S. aureus* (23.7%). For the remaining species, however, the overall genome coverage remained below 3%, significantly limiting the robustness of subsequent analyses.

#### Target Characterization: Network Analysis

For each bacterial species, we retained protein targets associated with at least one KEGG pathway, and subsequently conducted a protein–protein interaction (PPI) network analysis using STRING (both the Web server and the *STRINGdb* R-package), a database of protein–protein interactions.^53,54^ Given the importance of node centrality in identifying relevant targets within networks, we calculated degree, betweenness, and closeness centrality metrics from the STRINGdb-derived graphs with the *igraph* R-package.^55^ For each essential oil–bacterium pair, these centrality parameters were min–max normalized following the approach implemented in Cytoscape,^56,57^ and then integrated to compute a final score reflecting the overall centrality of each target.

#### Target FishingFocus on Molecules

All NPs component molecules were retrieved from PubChem,^58,59^ where each compound was associated with a unique CID code, with the exception of bornyl acetate due to unresolved stereochemistry. Canonical SMILES representations were downloaded for all compounds (Supporting Information, S2), excluding those reported only as “traces”. Molecule–target complementarity was assessed using BioGPS, a structure-based approach built on the FLAP procedure,^60^ which generates molecule–pocket alignments and evaluates complementarity through its *GlobalSum* scoring function that integrates GRID-derived MIFs (shape, hydrophobicity, and H-bonding interactions). *GlobalSum* (GS) scores typically fall within 0–1 range, although values exceeding 1 may occasionally be observed.

Normalization of GS scores was performed to account for different GS values distributions across molecules and pockets. Specifically, for each molecule–pocket pair *mol,poc*, two Z-scores were computed, namely *Zscore_mol*, which compares the GS value for a given molecule–pocket interaction with the scores of that molecule across all pockets, and *Zscore_poc*, which compares the same GS value with the distribution obtained for that pocket across a set of 100 representative drug-like molecules (Supporting Information, S4).

$$Zscore\text{\_}mol\left(mol,poc\right)=\frac{GlobSum\left(mol,poc\right)-mean\left(GlobSummolecule\right)}{sd\left(GlobSummolecule\right)}$$

$$Zscore\text{\_}poc\left(mol,poc\right)=\frac{GlobSum\left(mol,poc\right)-mean\left(GlobSumpocket\right)}{sd\left(GlobSumpocket\right)}$$

Their sum defined the *ZZscore*

ZZscore(mol,poc)=Zscore_mol(mol,poc)+Zscore_poc(mol,poc)

A similar normalization strategy has been previously described for docking results by Kim et al.,^61^ who applied a double normalization across ligands and receptors, assigning different weights to the two components (30% for ligand contribution and 70% for receptor contribution).

To associate BioGPS-derived interactions with specific protein target, results were grouped, for each molecule, using UniProt identifiers or gene names, given that a single protein may correspond to multiple PDB-derived pockets. For each molecule–target pair, the maximum ZZscore (among all pocket-level scores) was selected to define the molecule–target interaction. Specifically, *ZZscore_moltar* was computed as the maximum ZZscore of the molecule *mol* across the *n* pockets associated with the target *tar*.

$$ZZscore\text{\_}moltar\left(mol,tar\right)=\max\left\{ZZscore\left(mol,{poc}_{1}\right),...,ZZscore\left(mol,{poc}_{n}\right)\right\}$$

A deep analysis of a similar workflow, carried out in another context and published elsewhere,^62^ investigated the impact of the above-defined thresholds, and finally proposed the joint use of the following filters

ZZscore>2.0

Zscore_mol>1.0

Zscore_poc>1.0

In this study, these filters were applied selectively at key steps of the workflow.

#### Target Fishing Focus on Phytocomplexes

For each essential oil–target pair, a composite score was calculated by weighting the *ZZscore_moltar* values of individual molecules according to their relative abundance, excluding trace components. To prevent highly abundant molecules from dominating the score, weights were defined as

$$Weight=\{\begin{array}{l} perc\text{\_}comp,if\;perc\text{\_}comp<1\\ 1+\log_{10}(perc\text{\_}comp)\;if\;perc\text{\_}comp\geq 1 \end{array}$$

The weighted sum of ZZscores yielded the oil–target interaction score

$$WSumZZscore\text{\_}full\left(oil,target\right)=\sum_{mol=1}^{N}Weight\;\left(mol\right)\times ZZscore\text{\_}moltar\;\left(mol,target\right)$$

Because low or negative *ZZscore* values could artificially inflate the sum with weak and nonrelevant interactions, a refined score (*WSumZZscore_refined*) was also calculated. In this approach, *ZZscore_moltar* values were set to zero if the corresponding *ZZscore*, *Zscore_mol*, or *Zscore_poc* did not meet the predefined thresholds detailed above. Targets were then ranked first by decreasing *WSumZZscore_refined* and subsequently by *WSumZZscore_full*, generating sufficiently long ranked lists for downstream bioinformatics analyses. Based on these scores, phytocomplex–target interactions were categorized as follows: “strong” (*WSumZZscore_refined* > 0), “weak” (*WSumZZscore_refined* = 0 and *WSumZZscore_full* > 0), or “null” (*WSumZZscore_refined* = 0 and *WSumZZscore_full* ≤ 0). These ranked target lists were subsequently used for the analysis of each essential oil–bacterium pair.

#### Pathway Enrichment Analysis

The final step consisted of identifying the biological pathways enriched for each oil. For each essential oil–bacterium pair, ranked target lists were compared against KEGG pathway gene sets, using gene set enrichment analysis (GSEA),^63^ as implemented in the *clusterProfiler* R-package.^64–68^ Pathways with *p* < 0.05 were considered statistically significant, indicating that the ranked targets were nonrandomly enriched for genes associated with those pathways. As an additional refinement, pathways were discarded when the majority of contributing targets corresponded to null rather than strong or weak categories, as determined by *WSumZZscore_refined* values. This filtering step ensured that the final set of pathways reflected biologically meaningful interactions supported by the chemoinformatics analysis.

## Results

### Chemical Composition of EOs

The chemical composition of the three EOs was determined by GC–MS (Table 2). Cinnamon bark oil was dominated by cinnamaldehyde (54.40%) and eugenol (20.55%), which together accounted for the majority of its profile. In contrast, both oregano and thyme oils contained significant amounts of *o*-cymene, carvacrol, and thymol, although in different proportions: oregano was particularly rich in carvacrol, whereas thyme showed a higher relative abundance of thymol and was further distinguished by a notable d-limonene content (19%). Overall, oregano and thyme exhibited considerable compositional overlap, while cinnamon differed markedly from both. Some compounds were sought but not detected in all oils; their absence is indicated in Table 2 by the symbol “–”, whereas compounds present at very low levels (<0.1%) are reported as “traces”.

### Determination of Antibacterial Activity

All EOs exhibited antibacterial activity against *Staphylococcus* spp. and *E. coli*, with weaker effects on *Klebsiella pneumoniae* and *Pseudomonas aeruginosa* (Table 3); for these latter species we observed a solvent contribution to the activity, and a similar effect explained the low activity of thyme EO against *Enterococcus faecalis*. Across oils, cinnamon EO exhibited the lowest MICs against Gram-negative bacteria, key UTI pathogens, while maintaining activity against Gram-positives, thus showing the broadest antimicrobial spectrum. By contrast, thyme EO was the least effective, with the highest MICs in four of the six tested species. Across bacteria, *E. coli* was the most sensitive strain, whereas the three Gram-positive species showed comparable MICs. However, all EOs exhibited bactericidal activity, as indicated by their Minimum Bactericidal Concentrations (MBCs), which were comparable to, or at most 4-fold higher than, their MICs against each tested strain (Supporting Information, S3).

**Table 3:** EOs’ Minimum Inhibitory Concentration (MIC) Against Representative Bacterial Species, Reported as a Percentage Value and mg/mL

		*Cinnamomum zeylanicum*		*Origanum vulgare*		*Thymus vulgaris*		CIPROFLOXACIN	EtOH
		MIC	EtOH^a^	MIC	EtOH^a^	MIC	EtOH^a^	MIC	MIC
**Gram+**									
*Staphylococcus aureus* (ATCC29213)	%	0.16	1.6	0.16	1.6	0.16	1.6	0.0025	12.5
	mg/mL	1.58	12.63	1.53	12.63	1.48	12.63	0.00025	98.68
*Staphylococcus epidermidis* (RP62A)	%	0.08	0.8	0.08	0.8	0.16	1.6	0.0006	6.2
	mg/mL	0.79	6.32	0.77	6.32	1.48	12.63	0.00006	49.33
*Enterococcus faecalis* (ATCC 29212)	%	0.16	1.6	0.16	1.6	0.31	3.1	0.005	6.2
	mg/mL	1.58	12.63	1.53	12.63	2.87	25.26	0.0005	49.33
**Gram–**									
*Escherichia coli* (ATCC 25922)	%	0.04	0.4	0.08	0.8	0.16	1.6		12.5
	mg/mL	0.39	3.16	0.77	6.32	1.48	12.63	0.00008	98.68
*Klebsiella pneumoniae* (ATCC 700603)	%	0.31	3.1	0.31	3.1	0.62	6.2	0.0025	25
	mg/mL	3.06	25.26	2.97	25.26	5.74	48.94	0.00025	197.36
*Pseudomonas aeruginosa* (ATCC 27853)	%	0.16	1.6	1.25	12.5	1.25	12.5	0.005	>25
	mg/mL	1.58	12.63	11.98	98.67	11.58	97.88	0.0005	>197.36

a = percentage of EtOH 96% present in the MIC of each EO; Ciprofloxacin, a commonly prescribed antibiotic for UTIs, was included for comparison.

### Prediction of Molecule–Target Interactions

All molecules were subjected to target fishing, and the resulting data were aggregated into oil–bacterium target lists, which varied substantially across essential oils and bacterial species, reflecting differences in oil composition (Table 2) as well as in the availability of binding pockets for each organism (Table 1). As an initial analysis step, we focused on individual molecules, quantifying the number of putative targets identified for each compound, independently of their relative abundance in the oil. The results are summarized in Figure 2, with additional details provided in Supporting Information S8.

**Figure 2:**
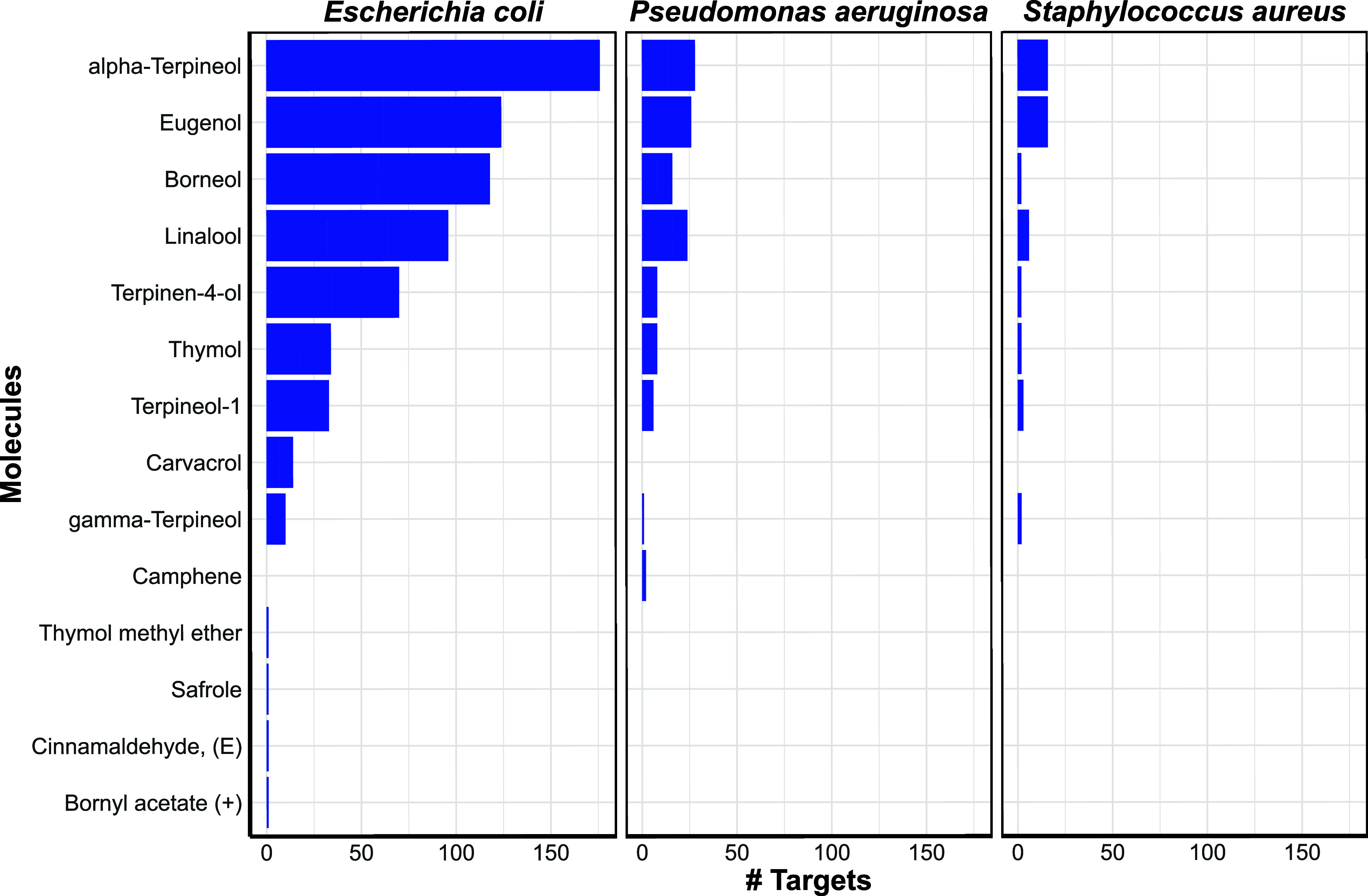
Bar plots of the number of targets identified for each molecule. Data are reported only for *E. coli*, *P. aeruginosa*, and *S. aureus*. For the other bacteria, the overall amount was very low, and the same plots are reported as Supplementary, S8.

Alpha-terpineol, eugenol, linalool, and borneol were predicted to interact with several targets across all bacteria: notably, eugenol and linalool are major components of the EOs, whereas borneol and α-terpineol are present at lower abundance; beyond these frequent hitters, several additional molecules contributed through more selective interactions.

Target analysis was conducted in two main steps: (i) evaluating network relevance using STRING (Web server and *STRINGdb* R-package) and computing *igraph*-derived centrality metrics (degree, betweenness, closeness), which were integrated into a single centrality score; and (ii) assessing pathway involvement through KEGG. The number of targets retrieved in STRING and the ten most central nodes for each oil–bacterium pair are reported in Table 4.

**Table 4:** Number of Targets Identified in STRING, and the Ten Most Central Nodes for Each Oil–Bacterium Pair, According to the Normalized Average of Parameters Degree, Betweenness, and Closeness^a^

bacteria	oils	input targets	STRINGdb targets found (%)	top ten central targets (decreasing value of the centrality score) for each bacterium–oil pair
*E. coli*	Cinnamon	982	612 (62.3%)	GuaA, BarA, NuoC, RpoA/RpoB, EntF, BirA, RecA, FtsN, RsmB, MetH
*E. coli*	Oregano	1027	654 (63.7%)	GuaA, ArcB, NuoC, **RpoA/RpoB**, BirA, RecA, FtsN, RsmB, MetH, LptC
*E. coli*	Thyme	1083	678 (62.6%)	GuaA, ArcB, BarA, NuoC, **RpoA/RpoB**, EntF, BirA, RecA, FtsN, RsmB
*P. aeruginosa*	Cinnamon	201	138 (68.7%)	Eft; GyrB; **Hfq**; CoaD; IlvE; TrmD; FolD; MurA; LysC; AlgL
*P. aeruginosa*	Oregano	209	143 (68.4%)	Efp, MucA/MucB, GyrB, Hfq, TrmD, IlvE, CoaD, FolD, MurA, LysC
*P. aeruginosa*	Thyme	229	156 (68.1%)	Efp, MucA/MucB, GyrB, Hfq, TrmD; IlvE, CoaD, FolD, MurA, LysC
*S. aureus*	Cinnamon	183	106 (57.9%)	**Spa**, Eno, **GlyA**, Ndk, Map, **FtsZ**, CshA, **TrmD**, ClfA, Pnp
*S. aureus*	Oregano	189	108 (57.1%)	**Spa**, Eno, GlyA, Ndk, **FtsZ**, CshA, **TrmD**, Pnp, Ldh1, MenE
*S. aureus*	Thyme	204	118 (57.8%)	**Spa**, Eno, GlyA, Ndk, Map, **FtsZ**, CshA, **TrmD**, ClfA, Pnp

a The nodes with *WSumZZscore* values in the top 20% are reported in bold. All data is available in the sheet “Centrality” of Results.xlsx (Supporting Information).

Among the three bacteria with the highest number of predicted targets (*E. coli*, *P. aeruginosa*, *S. aureus*), most of the top-ranked central proteins (sorted by decreasing centrality) were not effectively hit by EO molecules. The only exception was LysC in *P. aeruginosa*, ranked ninth–10th across oils and predicted to interact with linalool. Other highly central proteins (Table 4) received low scores because many molecule–target interactions were filtered out by the applied Zscore and ZZscore thresholds. Nevertheless, several targets remain noteworthy, ranking within the top 10% of their respective bacterial lists (for at least one EO): ClpP, CarA/CarB, PckA, and Crp in *E. coli*; LpxC and FtsI in *P. aeruginosa*; and Spa, AaaA/AccD, ClpP, MoaA, IlvC, and PanC in *S. aureus*.

A critical step involved manual curation of the target lists: expanding the analysis to additional strains introduced the risk of including proteins absent from the experimentally tested ATCC strains, while using the entire BioGPS pocket collection raised the possibility of including unreliable PDB structures. To mitigate these, we verified gene presence in the ATCC genomes by integrating data from ATCC, UniProt, and KEGG web-portals (Supporting Information, S9). Among the top 30 genes per oil–bacterium pair, approximately 14–18% were removed (11/61 for *E. coli*, 8/49 for *P. aeruginosa*, 4/27 for *S. aureus*).

Pocket suitability was assessed at both the PDB and pocket levels (Supporting Information, S10). At the PDB level, entries unrelated to drug design, involving nonrelevant mutants, or using *E. coli* solely as an expression system were excluded (removing 3/28 entries for *S. aureus*, 9/53 for *P. aeruginosa*, and 98/209 for *E. coli*). At the pocket level, all 250 curated pockets exhibited acceptable geometries; a few external but concave pockets were retained. An illustrative example of corrective refinement involved Spa (staphylococcal protein A^69^) in *S. aureus*, initially predicted to interact with α-terpineol through pocket 5cbn1_003. Manual inspection of PDB entry 5CBN^70^ revealed it to be a fusion protein, raising concerns about the reliability of the predicted binding-site in drug design scenarios, and prompting its exclusion.

The full manual curation workflow is detailed in Supporting Information (S9 and S10), whereas Figure 3 summarizes results for the top 20 genes of each oil–bacterium pair after curation. For each bacterium, the heatmap shows the contribution of the three oils in terms of molecule–target interactions: specifically, *ZZscore* values appear as red cells, bar plots reflect the relative abundance of individual molecules in each oil, and an additional column denotes target centrality. Aggregated data for all three oils, integrating composition and ZZscore values, are shown in the accompanying scatterplots. Overall, targets are ranked by decreasing *WSumZZscore_refined* values averaged across oils, with each oil–bacterium pair contributing with its top 20 targets.

**Figure 3:**
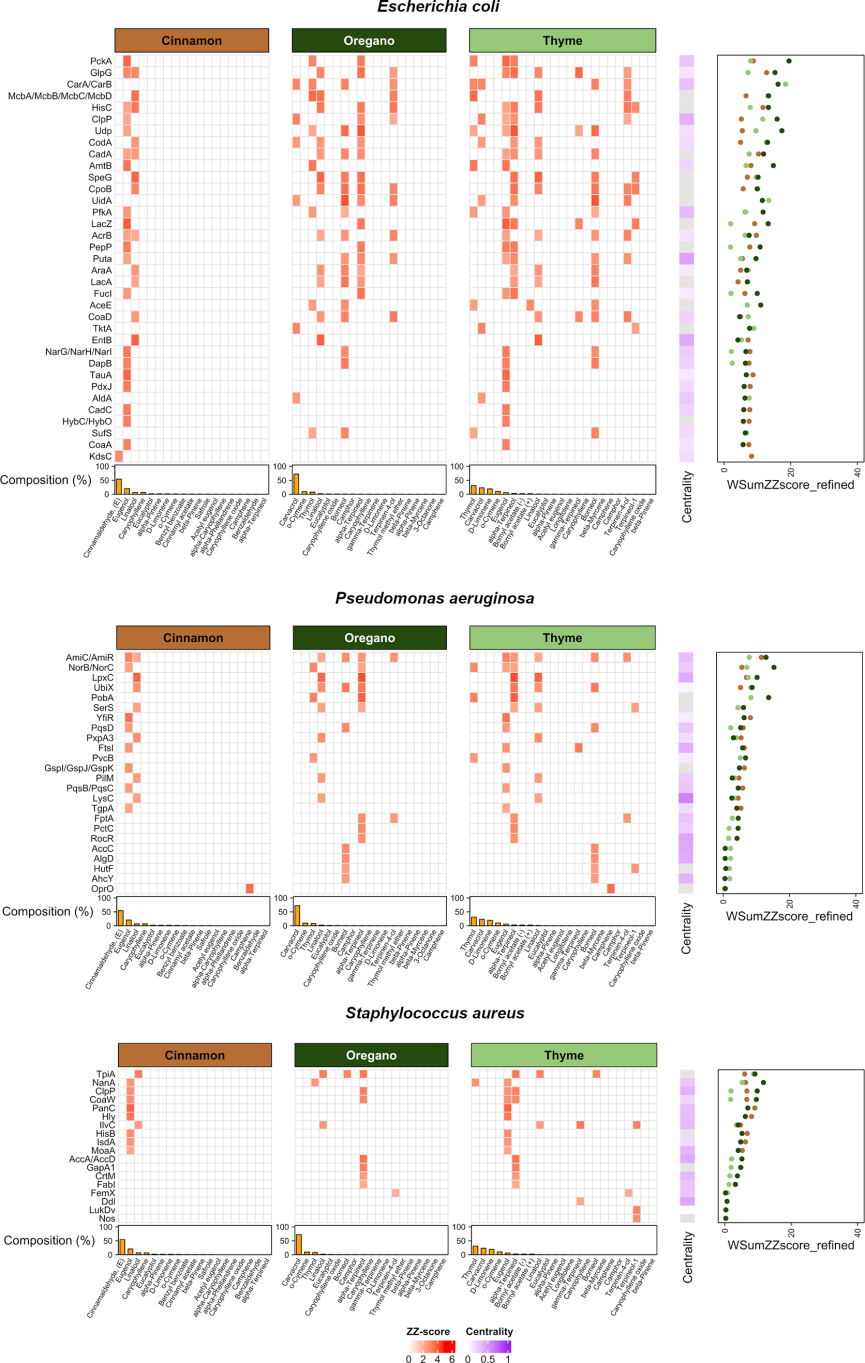
Heatmap with the most relevant targets from the BioGPS analysis for *E. coli*, *P. aeruginosa* *,* and *S. aureus*, including composition data (Bar plots), molecular *ZZscores* (heatmaps), network analysis (Centrality column in the heatmap), and *WSum_ZZscore_refined* for phytocomplexes (weighted sum of ZZscores, scatterplots). The plot is based on the R-package *ComplexHeatmap*.^71–73^

These visualizations allow exploration of both multihit targets and singletons (targets predicted to interact with only one molecule). The upper portion of the heatmaps highlights several multihit targets, including CarA/CarB, HisC, and CodA in *E. coli*, AmiC/AmiR, NorB/NorC, and UbiX in *P. aeruginosa*, and TpiA and IlvC in *S. aureus*. Numerous singletons are also present. For several of these targets, we performed literature validation, as discussed in the corresponding Results section.

### Pathway Enrichment Analysis

The curated target lists were evaluated through pathway enrichment analysis. Because enrichment results can be strongly biased by pathway size, we applied a pathway-size threshold (>1000 genes) to exclude overly broad KEGG categories such as *Metabolic pathways* and *Biosynthesis of cofactors*. Significant pathways were identified only for *E. coli*, *P. aeruginosa*, and *S. aureus*, with results summarized in Figure 4. In total, 30 pathways were enriched in *E. coli*, eight for *P. aeruginosa*, and two for *S. aureus*. In the heatmap, cell numbers denote both the count of pathway-associated targets predicted to interact with EO molecules and the number of molecules contributing to these interactions, whereas cell color reflects statistical significance (darker shading = lower *p*-value). For *E. coli*, each oil exhibited its own set of preferentially enriched pathways, whereas several pathways were shared across oils, indicating a combination of either selective or overlapping mechanisms of action, respectively.

**Figure 4:**
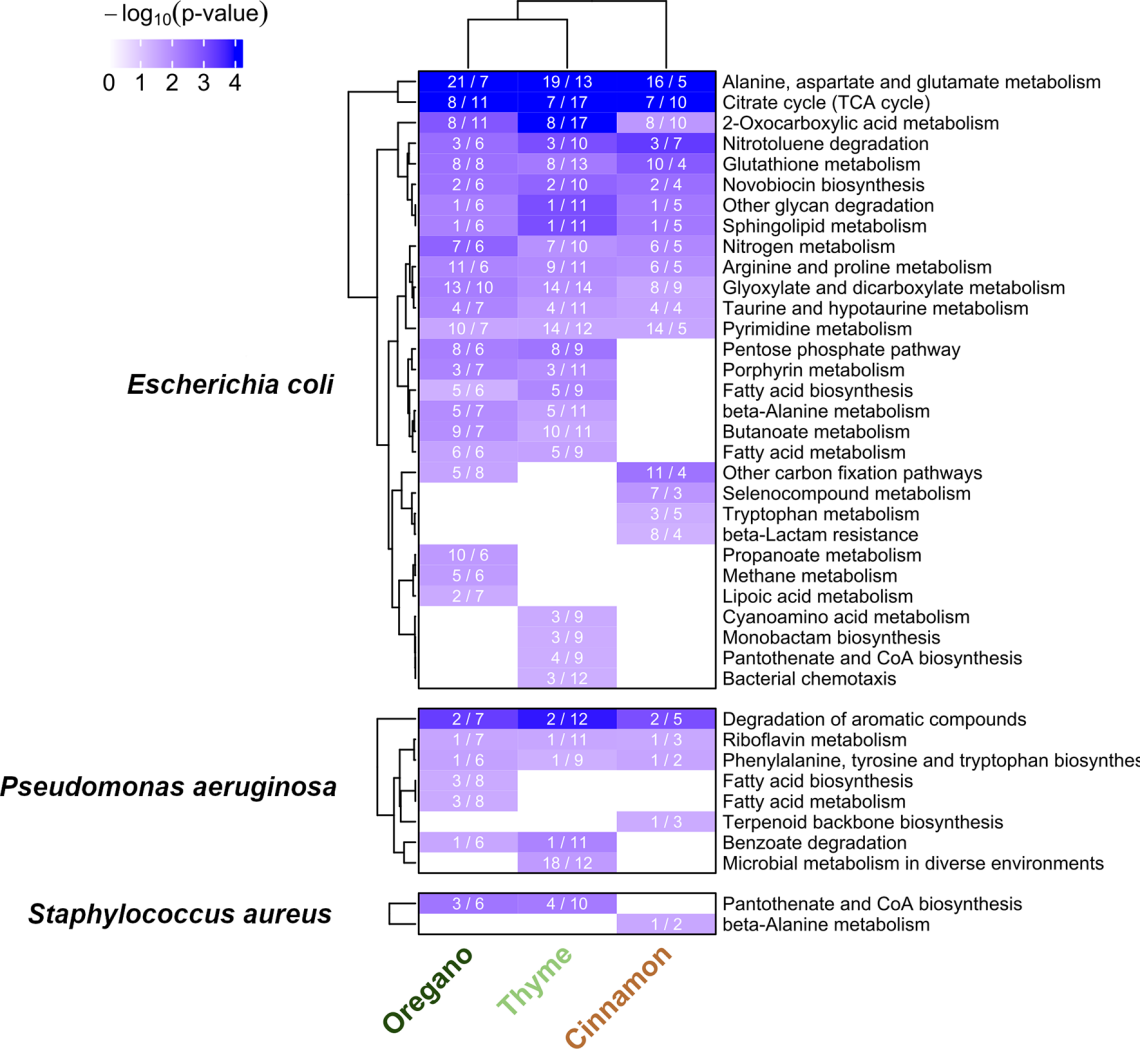
Heatmap for the enriched pathways for the three oils and *E. coli*, *P. aeruginosa*, and *S. aureus* bacteria. For the remaining bacteria, no significant pathway was retrieved. Numbers in each cell report tar/mol: tar is a counter for how many targets of the pathway are hit, by how many molecules overall (mol). The plot is based on the R-package *ComplexHeatmap*.^71–73^ White cells simply denote interactions that did not reach statistical significance. Notably, the same set of targets can yield different scores for different oils depending on their molecular composition, leading to variations in *p*-values.

The complexity of molecule–target–pathway relationships can be effectively visualized using alluvial plots (based on the R-package *ggalluvional*),^74,75^ which depict how individual targets and their associated molecules contribute to each biological pathway. Figure 5 illustrates several representative pathways, emphasizing the multiligand/multitarget nature of the interactions: certain targets are modulated by multiple molecules, while some molecules engage with more than one target. Comprehensive alluvial plots covering all significant pathways are provided in Supporting Information S11.

**Figure 5:**
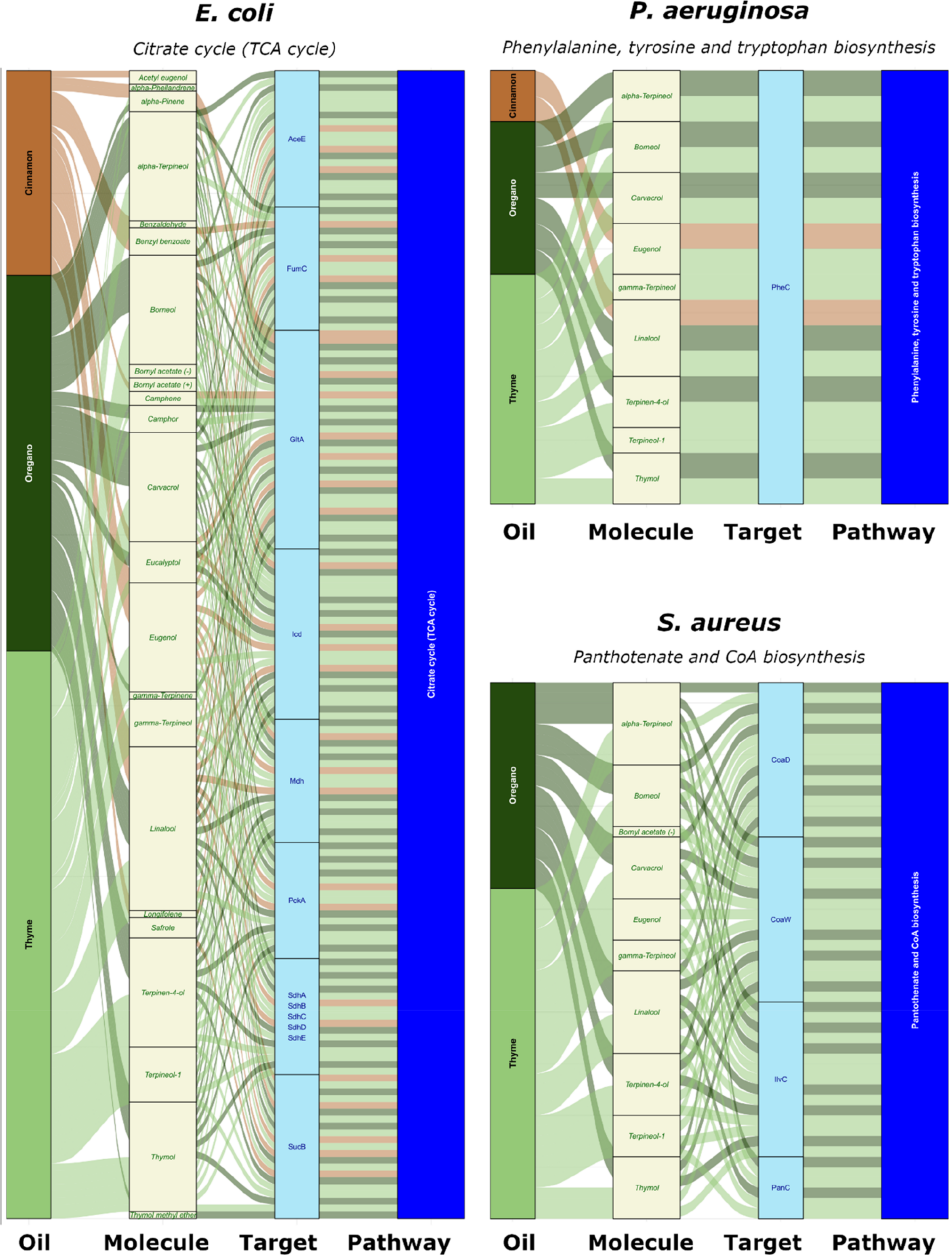
Alluvial plots for representative pathways of *E. coli*, *P. aeruginosa*, and *S. aureus*.

## Discussion

Natural medicines are receiving increasing attention due to their unique chemical scaffolds, potential synergistic effects, favorable safety profiles, and environmental sustainability.^76–80^ In studies involving complex mixtures of natural phytocompounds—referred to here as phytocomplexes—the identification of a biological activity is often only the first step, while elucidating the underlying molecular mechanisms is typically far more challenging and time-consuming, yet remains essential for clinical translation. Despite this need, to our knowledge no computational approaches currently exist that systematically generate mechanistic hypotheses for such chemically diverse and complex mixtures. To address this gap, we propose a structure-based strategy that complements existing ligand-based methodologies:^81,82^ our computational approach integrates chemoinformatics and bioinformatics to investigate the mechanisms of action of complex mixtures of natural products.

We applied the pipeline to cinnamon, oregano, and thyme EOs in the context of UTIs. Antibacterial assays confirmed activity against both Gram-positive and Gram-negative bacteria, with cinnamon exhibiting the strongest potency and thyme the weakest. Recognizing that certain mechanisms, such as membrane disruption by lipophilic molecules require different modeling strategies, we focused here on molecular scenarios in which specific molecule–target interactions play a central role. We are convinced that EO-mediated bacterial killing cannot be fully explained by nonspecific membrane perturbation alone. The novel target fishing procedure introduced in this study, based on normalization and aggregation of BioGPS scores, provides a systematic framework to link phytocomplex molecules with putative protein targets.

Through this process, we identified a refined panel of bacterial targets exhibiting high predicted complementarity to EOs components. To contextualize these targets within the cellular landscape, we integrated protein–protein interaction data from STRING and applied network centrality metrics to assess the relative importance of each protein within the bacterial interactome. These curated target sets were then analyzed using established bioinformatics pipelines to identify enriched biological pathways potentially modulated by EO molecules, thereby generating testable mechanistic hypotheses for the observed antibacterial effects.

In the following sections, we discuss in detail the main findings arising from the target fishing analysis and subsequent pathway enrichment results.

### Target Fishing

The computational workflow produces, for each molecule–target pair, complementary information including PDB entry, binding pocket name, number of the pockets per target, percent composition, and score values (Supporting Information, xlsx). Among the bacteria analyzed, we focused on *E. coli* due to its major contribution to UTIs (∼80%)^83,84^ and its extensive genome–pocketome coverage (Table 1). For several top-ranked targets (Figure 3, Table 5), we conducted a literature review to assess their biological relevance. Nevertheless, targets predicted for only a few molecules may still be meaningful and warrant further examination. An illustrative example is KdsC, a promising antibacterial target^85^ involved in the biosynthesis of 3-deoxy-d-manno-octulosonate (KDO), an essential precursor of lipopolysaccharide required for membrane biogenesis and bacterial viability; this enzyme was selectively targeted by cinnamaldehyde from cinnamon EO.

**Table 5:** Some of the Most Relevant Targets for *E. coli*

target	details	notes
PckA	UniProt identifier: P22259 PCKA_ECOLI phosphoenolpyruvate carboxykinase	It catalyzes the conversion of oxaloacetate (OAA) to phosphoenolpyruvate (PEP), a key step in gluconeogenesis. Although this enzyme is required for metabolic adaptation during growth on nonglucose carbon sources, it is not essential for survival under nutrient-rich or glucose-containing conditions
GlpG	UniProt identifier: P09391 GLPG_ECOLI Rhomboid protease GlpG	It catalyzes intramembrane proteolysis and contributes to colonization capacity in pathogenic E. coli by maintaining membrane protein homeostasis. Several studies have demonstrated functional roles for rhomboid proteases^86^ and proposed that they participate in bacterial communication through diffusible chemical signals.^87^ These intramembrane proteases therefore likely play an essential role in bacterial physiology, particularly in membrane protein quality control
CarA	UniProt identifier: P0A6F1 CARA_ECOLI	Both the small and large subunits of glutamine-dependent carbamoyl phosphate synthetase (CPSase) were identified. CPSase catalyzes the formation of carbamoyl phosphate, the first committed step in biosynthesis of arginine, urea, and pyrimidine nucleotides, which are pathways essential for bacterial growth and survival.^88^ Across the series, this enzyme was predicted to interact with several EOs components, including molecules found at high abundance (α-terpineol, carvacrol, eugenol, linalool, and thymol). Inhibition of CPSase could disrupt arginine and pyrimidines biosynthesis, leading to severe growth defects and potentially bactericidal effects in the absence of external supplementation
CarB	UniProt identifier: P00968 CARB_ECOLI	
McbA	UniProt identifier: P05834 MCBA_ECOLX	Oregano and thyme showed higher scores than cinnamon for the McbA/B/C/D complex, which produces the bacteriocin microcin B17, a peptide toxin used by bacteria to inhibit the growth of competing strains.^89^ Perturbation of this system may impair competitive fitness or interbacterial interactions
McbB	UniProt identifier: P23184 MCBB_ECOLX	
McbC	UniProt identifier: P23185 MCBC_ECOLX	
McbD	UniProt identifier: P23186 MCBD_ECOLX	
HisC	UniProt identifier: P06986 HIS8_ECOLI, Histidinol-phosphate aminotransferase	It is a key enzyme in histidine biosynthesis,^90^ a pathway particularly important in nutrient-limited environments where histidine availability may be low^91^
ClpP	UniProt identifier: P0A6G7 CLPP_ECOLI ATP-dependent Clp protease proteolytic subunit	It participates in the degradation of misfolded, aggregated, and toxic peptides and proteins; thus, its inhibition can lead to loss of proteostasis and cellular death. A recent review^92^ identifies ClpP as a promising target for novel antibiotics, and several inhibitors have been reported, although none are yet approved due to toxicity concerns. Acyldepsipeptides (ADEP) activate and dysregulate ClpP, leading to structural alterations.^93^ ClpP is central to numerous essential pathways across diverse bacterial species, with substrates involved in the cell cycle, metabolism, stress tolerance, virulence regulation, antibiotic tolerance, and biofilm formation. Disruption of ClpP activity is therefore lethal in many organisms.^94,95^
Udp	Uniprot identifier: P12758 UDP_ECOLI Uridine phosphorylase	It plays a central role in salvaging uracil from uridine.^96^ Due to redundancy in pyrimidine salvage pathways, inhibition of uridine phosphorylase is expected to impair bacterial growth by limiting the availability of pyrimidine precursors, but is unlikely to be lethal unless combined with inhibition of complementary pathways, as demonstrated by the knockout of the NAD^+^ salvage enzyme xapA in E. coli, which reduces growth rate but remains nonlethal^97^
CodA	UniProt identifier: P25524 CODA_ECOLI heterologously expressed cytosine/isoguanine deaminase	It is the only enzyme in E. coli capable of deaminating isoguanine, a role confirmed by knockout experiments in which deletion of CodA resulted in a pronounced reduction of isoguanine deaminase activity compared to the wild-type strain^98^
CadA	UniProt identifier: P0A9H3 LDCI_ECOLI Inducible lysine decarboxylase	It contributes to pH homeostasis by consuming protons during the decarboxylation reaction, a mechanism essential for E. coli survival under acidic conditions but dispensable at neutral pH^99^
AmtB	UniProt identifier: P69681 AMTB_ECOLI Ammonium transporter	It is a protein with a key role in the ammonium acquisition, which is vital for nitrogen assimilation, a fundamental metabolic requirement; however, bacteria can alternatively fulfill their nitrogen needs through other routes, such as via amino acid uptake^100^
SpeG	UniProt identifier: P0A951 ATDA_ECOLI Spermidine N(1)-acetyltransferase	It regulates intracellular spermidine levels, an essential molecule required for DNA packaging, RNA stability, and oxidative stress tolerance; by preventing polyamine toxicity, particularly under stress or nutrient imbalance, it contributes to cellular homeostasis. Its modulation may become relevant when acting synergistically with other mechanisms^101^
CpoB	UniProt identifier: P45955 CPOB_ECOLI Cell division coordinator	It is a periplasmic protein with a key coordinating role during cell division, synchronizing peptidoglycan synthesis with the outer membrane constriction machinery, and is therefore essential for proper cell division and cell envelope integrity.^102^ When the CpoB function is disrupted, peptidoglycan synthesis and outer membrane remodeling become uncoupled, resulting in severe envelope destabilization and ultimately bacterial cell death

Overall, the predicted targets cover a wide spectrum of functions, including energy production pathways, biosynthesis of essential metabolites, and quality-control processes that preserve membrane integrity. While many targets may not be individually essential (due to functional redundancy of bacterial systems) the concurrent action of multiple EO constituents on diverse cellular processes suggests potential synergistic effects leading to bacterial death. Experimental validation of individual or combined targets falls outside the scope of this study, and we acknowledge the possibility of false positives. Nonetheless, the generated target lists for all tested bacteria represent a rich resource that can guide future mechanistic studies and experimental investigations.

### Pathways Analysis

Transitioning from target fishing to pathway enrichment allows to move beyond individual proteins and examine groups of biologically connected targets within the same pathway, thereby revealing potential multiligand/multitarget synergies. Using gene set enrichment analysis (GSEA), we identified statistically significantly enriched pathways (*p* < 0.05) for *S. aureus* (2), *P. aeruginosa* (8), and *E. coli* (30). Considerable overlap was observed: 13 pathways in *E. coli* and 3 in *P. aeruginosa* were shared across all three oils, while thyme and oregano shared additional pathways. Singular pathways specific to individual oils were also detected, suggesting selective mechanisms of action.

For *E. coli*, the pathway with the highest coverage was *Alanine*, *aspartate*, and *glutamate metabolism*, with 21, 19, and 16 targets hit by oregano (with 7 different molecules), thyme (13 molecules), and cinnamon (5 molecules), respectively. This pathway is linked to nitrogen balance and clusters with other significant metabolic routes, including *Nitrogen metabolism*, *Arginine* and *Proline metabolism*, *Pyrimidine metabolism* and many others.

Other enriched pathways involve amino acid metabolism, cofactor and vitamin biosynthesis (e.g., *Pantothenate* and *CoA biosynthesis*, *Porphyrin metabolism*), lipid metabolism (*Fatty acid biosynthesis*/*metabolism*, *Sphingolipid metabolism*, *Lipoic acid* *metabolism*), and energy production (*Citrate cycle*, *2-Oxocarboxylic acid metabolism*). Inhibition of these pathways can disrupt carbon metabolism and redox balance, ultimately reducing bacterial growth and viability. Alluvial diagrams (Figure 5) illustrate oil–molecule–target–pathway relationships, highlighting representative pathways for each bacterium; for example, in the *E. coli* *Citrate cycle*, linalool and eugenol interact with multiple targets, consistent with literature reports of linalool inhibiting TCA cycle enzymes in *Pseudomonas fluorescens*.^103^ These observations demonstrate the utility of this integrative approach for guiding literature searches and experiment design, and future investigations into the mechanisms of essential oils.

## Conclusions

We present a pipeline that integrates chemoinformatics and bioinformatics for the study of complex mixtures of bioactive natural compounds, such as essential oils, to help generate hypotheses and experimental prioritization. By matching phytocomplex composition data with bacterial binding pockets, we identified complementary target–molecule pairs and translated these findings into pathway-level insights. This approach adapts enrichment techniques typically applied to gene expression data and, to our knowledge, represents the first structure-based strategy to predict putative pathways directly from phytocomplex composition. Although experimental validation was beyond the scope of this study, several top-ranked targets and pathways were discussed, providing a foundation for future investigations. Our findings indicate that EOs can modulate multiple bacterial pathways, particularly those associated with energy production and DNA/RNA synthesis. Because bacterial metabolism strongly influences antibiotic efficacy, resistance, and clinical outcomes, metabolism-based strategies may support personalized antimicrobial therapies,^104^ motivating further studies that could incorporate omics analyses. While our method identifies putative pathways, it does not distinguish between induction, activation, or inhibition of individual targets, and it cannot directly assign bactericidal mechanism versus bacteriostatic.^105,106^

In the context of UTIs, inhibiting energy and nucleic acid pathways may reduce bacterial virulence, whereas interference with protein export could impair toxin release and adhesion. Conversely, stimulation of specific metabolic routes could enhance antibiotic lethality; for instance, upregulation of amino acid metabolism, the TCA cycle, or nucleotide metabolism has been proposed to restore antibiotic effectiveness.^106–108^ Accordingly, combining EOs with standard UTI treatments (nitrofurantoin, fosfomycin, trimethoprim-sulfamethoxazole, fluoroquinolones^109^) could enhance antimicrobial activity, reduce required doses, and help overcome resistance.

However, it must be emphasized that, despite the potential of EOs to enhance standard UTI therapies through bacterial metabolic modulation, their practical application might be limited by intrinsic physicochemical constraints. EOs are mixtures of highly unstable, volatile, and irritant lipophilic compounds with poor systemic bioavailability,^110^ where insufficient absorption and rapid metabolism can prevent adequate concentrations from reaching the urinary tract. Accordingly, the development of formulations capable of improving stability,^111^ enhancing absorption,^112^ and enabling controlled release^111^ indicates that this route is indeed feasible for translating mechanistic insights into clinically applicable interventions. In this context, our work contributes to defining the activity of individual components as a foundation for their informed use, once appropriately formulated, within the target pathological network.

Further refinement of the computational procedure, including more detailed analyses of molecule–target interactions, will help confirm or rule out mechanistic hypotheses. Our conservative approach was designed to preserve comprehensive information, leaving prioritization to researchers’ expertise. Ultimately, this framework may accelerate mechanistic studies of phytocomplexes and support the development of metabolism-based antimicrobial strategies.

Our computational pipeline and predicted targets provide a valuable resource to guide both our group and the wider research community in the rational selection of EO-antibiotic combinations and the design of targeted experiments to elucidate phytocomplex activity in urinary infections.

## Supplementary Material





## Data Availability

Scripts and aggregated data to reproduce the results are available on GitHub (https://github.com/caddunits/biogps_essoils). While some licensed input data (e.g., ATCC) cannot be shared, all essential information is deposited in a public repository (figshare) and detailed in the Supporting Information.

## Notes

Results.xlsx (XLSX)
Chemical characterization of the essential oils (including GC–MS chromatograms of the three essential oils, canonical SMILES codes, and retention times of the studied compounds); biological characterization (including minimum bactericidal concentration MBC values of the essential oils against the tested bacterial species); procedure for the selection of reference molecules from the DrugCentral database, and the complete set of 100 reference molecules; procedures for data extraction from the ATCC Genome Portal and the KEGG database; procedure for linking PDB entries to UniProt accession codes and gene names; bar plots reporting the number of identified targets for each molecule in *E. faecalis* and *K. pneumoniae*; manual curation of gene targets from the ATCC database and KEGG pathways for the strains used in the experimental assays; manual curation of gene targets associated with corresponding PDB entries; alluvial plots; complete workflow of the study, including scripts with input files, outputs, and detailed descriptions (PDF)
