# Supplementary material for: Thyme, Oregano, and Cinnamon Essential Oils: Investigating Their Molecular Mechanism of Action for the Treatment of Bacteria-Induced Cystitis
Source: ACS Omega. 2026 Jan 12;11(6):9757–73. doi: 10.1021/acsomega.5c10256 (PMC12917644; doi:10.1021/acsomega.5c10256)
Supplement: Supplementary file 2 [file ao5c10256_si_002.pdf]

SUPPLEMENTARY MATERIAL

Thyme, Oregano, and Cinnamon Essential Oils: Investigating their Molecular Mechanism of Action for the Treatment of Bacteria-Induced Cystitis

Emanuele Carosati[1,*], Laura Beatrice Mattioli[2], Alberto Santini[3], Giovanni Caprioli[4], Matteo Micucci[5,6], Gianmarco Mangiaterra[5], Carla Marzetti[3], Maria Scola Gagliardi[3], Franks Kamgang Nzekoue[4], Sauro Vittori[4], Giovanni Scala[7], Michele Ceccarelli[8,9], Maria Frosini[10], Ivan Corazza[11], Roberta Budriesi[2,*]

[1]Chemical and Pharmaceutical Sciences Department, University of Trieste, Trieste, Italy
[2]Pharmacy and Biotechnology Department, Alma Mater Studiorum-University of Bologna, Bologna, Italy
[3]Valsambro S.r.l., Bologna, Italy
[4]School of Pharmacy, University of Camerino, Camerino, Italy
[5]Biomolecular Sciences Department, University of Urbino "Carlo Bo", Urbino, Italy
[6]UniCamillus – Saint Camillus International University of Health Sciences, Rome, Italy
[7]Biology Department, University of Naples "Federico II", Naples, Italy
[8]Electrical Engineering and Information Technology Department, University of Naples "Federico II", Naples, Italy
[9]BIOGEM Institute of Molecular Biology and Genetics, Ariano Irpino, Italy
[10]Life Sciences Department, University of Siena, Siena, Italy
[11]Medical and Surgical Sciences Department, Alma Mater Studiorum-University of Bologna, Bologna, Italy

*** corresponding authors**: EC emanuele.carosati@units.it +39(0)405582732 RB roberta.budriesi@unibo.it +39(0)51209737

Table of Contents

Section	Page(s)	Content
S1	2-3	Chemical characterization: GC-MS chromatograms for the three essential oils.
S2	3-4	Canonical SMILES code for the studied compounds, and retention time.
S3	5	Biological characterization: EOs' Minimum Bactericidal Concentration (MBC) against the tested bacterial species, reported as a percentage value and mg/mL.
S4	6	Procedure to select the reference molecules from DrugCentral, including the set of 100 reference molecules
S5	6	Procedure to extract data from the website of the ATCC Genome Portal.
S6	7-10	Procedure to access data from the KEGG database.
S7	11	Procedure to access data to link PDB entries to UniProt codes and gene names.
S8	11	Bar plots of the number of targets identified for each molecule for *E. faecalis* and *K. pneumoniae*
S9	12-17	Manual curation of gene targets on ATCC website for the strains used in the experimental tests, and in the KEGG pathways.
S10	18-23	Manual curation of gene targets referring to the corresponding PDB entries.
S11	24-48	Alluvial plots.
S12	49-53	Sequence of all the steps (scripts with input, output and description).

Section S1. GC-MS chromatograms for the three essential oils.

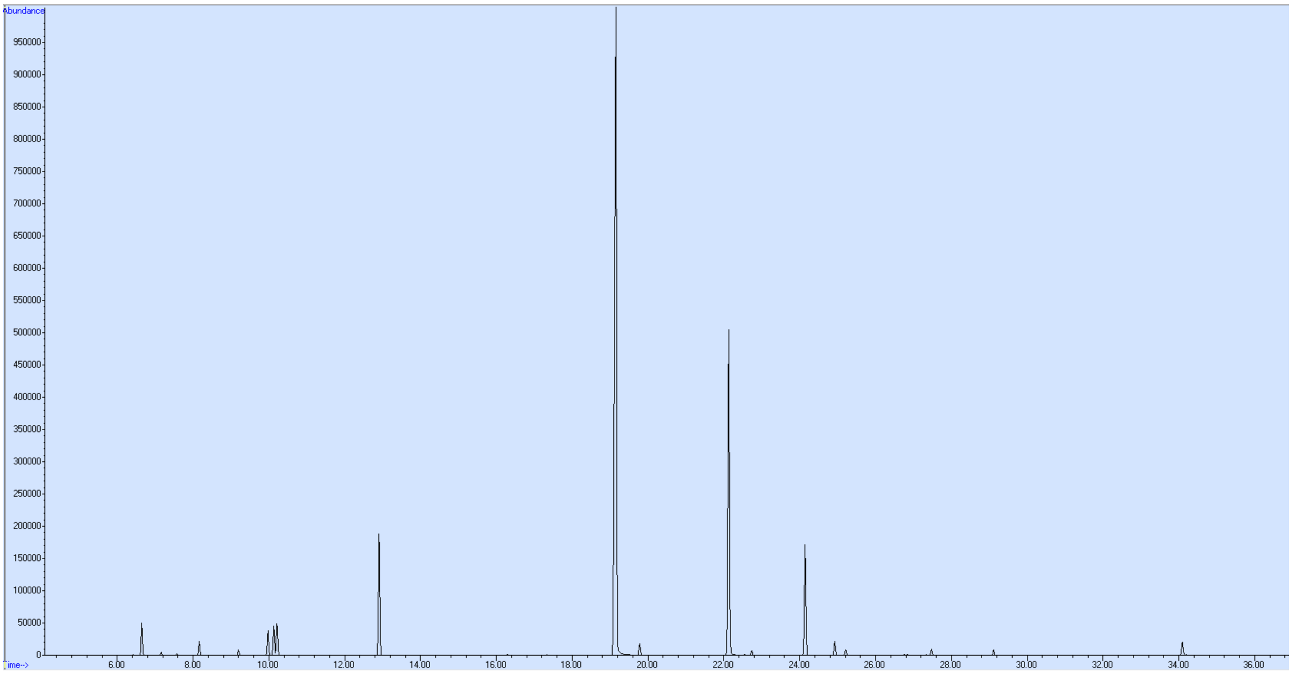

GC-MS chromatogram of ***Cinnamomum zeylanicum*** EO.

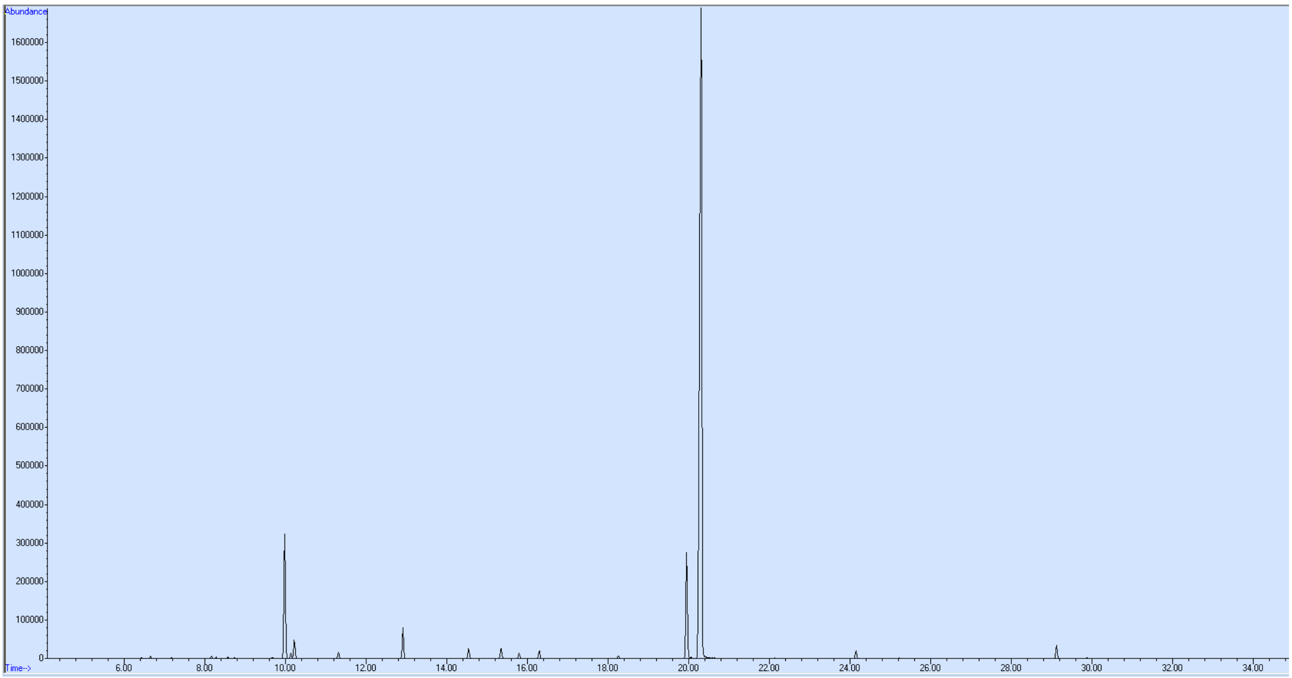

GC-MS chromatogram of ***Origanum vulgare*** EO.

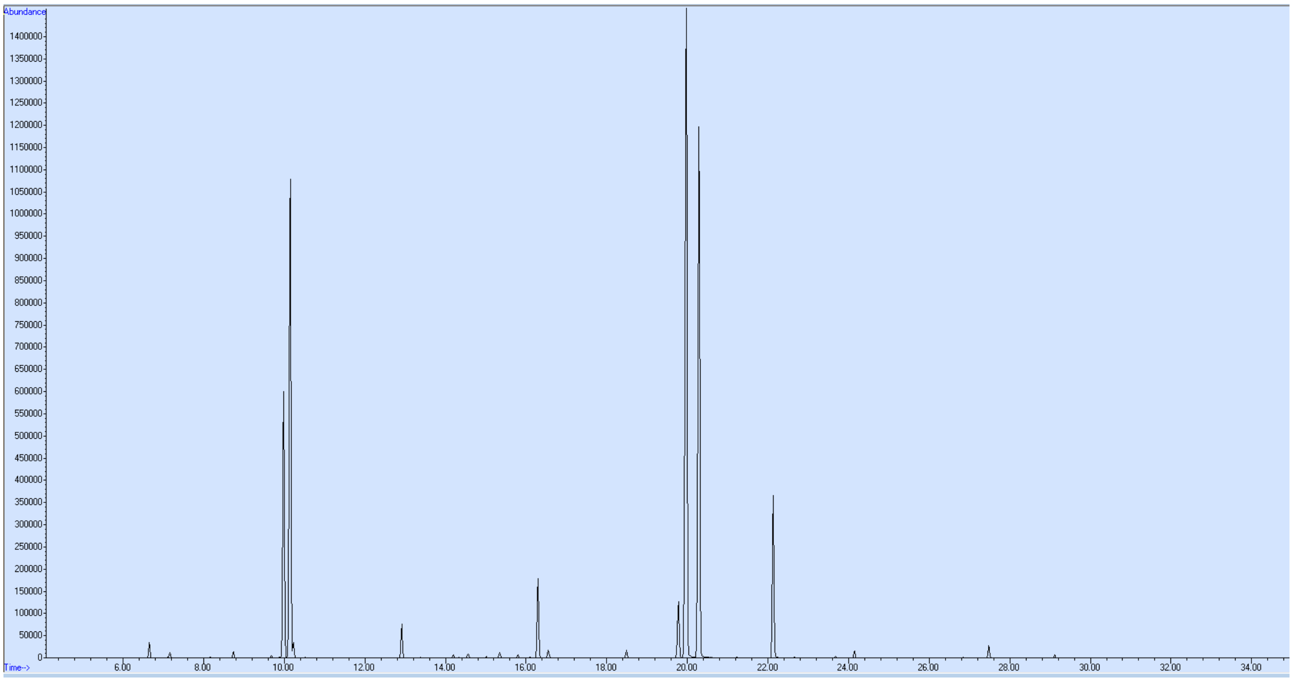

GC-MS chromatogram of ***Thymus vulgaris*** EO.

Section S2. Canonical SMILES codes for the studied compounds, and detailed identification procedure.

CID	Compounds	SMILES	RT[a]	RI Calc[b]	RI Lit[c]
**Monoterpenoids**					
**7460**	α-Phellandrene	CC1=CCC(C=C1)C(C)C	9.2	1004	1002
**6654**	α-Pinene	CC1=CCC2CC1C2(C)C	6.7	933	932
**31253**	β-Myrcene	CC(=CCCC(=C)C=C)C	8.7	991	988
**14896**	β-Pinene	CC1(C2CCC(=C)C1C2)C	8.2	975	974
**6616**	Camphene	CC1(C2CCC(C2)C1=C)C	7.2	947	946
**440917**	D-Limonene	CC1=CC[C@@H](CC1)C(=C)C	10.1	1028	1024
**7461**	γ-Terpinene	CC1=CCC(=CC1)C(C)C	9.7	1016	1014
**10703**	o-Cymene	CC1=CC=CC=C1C(C)C	10.0	1024	1022
**Oxygenated monoterpenoids**					
**17100**	α-Terpineol	CC1=CCC(CC1)C(C)(C)O	16.3	1190	1186
**64685**	Borneol	CC1(C2CCC1(C(C2)O)C)C	15.3	1165	1165
**93009**	(-)Bornyl acetate	CC(=O)O[C@@H]1C[C@@H]2CC[C@]1(C2(C)C)C	19.7	1286	1287
**6950274**	(+)Bornyl acetate	CC(=O)O[C@H]1C[C@H]2CC[C@@]1(C2(C)C)C			
**2537**	Camphor	CC1(C2CCC1(C(=O)C2)C)C	14.5	1142	1141
**10364**	Carvacrol	CC1=C(C=C(C=C1)C(C)C)O	20.3	1303	1298
**2758**	Eucalyptol	CC1(C2CCC(O1)(CC2)C)C	10.2	1030	1026
**11467**	γ-Terpineol	CC(=C1CCC(CC1)(C)O)C	16.5	1197	1199
**6549**	Linalool	CC(=CCCC(C)(C=C)O)C	12.9	1100	1095
**11230**	Terpinen-4-ol	CC1=CCC(CC1)(C(C)C)O	15.8	1176	1174
**11468**	Terpineol-1	CC(C)C1=CCC(CC1)(C)O	14.2	1134	1130

CID	Compounds	SMILES	RT[a]	RI Calc[b]	RI Lit[c]
**6989**	Thymol	CC1=CC(=C(C=C1)C(C)C)O	19.9	1292	1289
**14104**	Thymol methyl ether	CC1=CC(=C(C=C1)C(C)C)OC	18.3	1245	1231
**Phenylpropanoids**					
**7136**	Acetyl eugenol	CC(=O)OC1=C(C=C(C=C1)CC=C)OC	27.5	1528	1521
**637511**	Cinnamaldehyde, (E)	C1=CC=C(C=C1)/C=C/C=O	19.2	1270	1267
**5282110**	Cinnamyl acetate	CC(=O)OC/C=C/C1=CC=CC=C1	24.9	1445	1443
**3314**	Eugenol	COC1=C(C=CC(=C1)CC=C)O	22.1	1358	1356
**5144**	Safrole	C=CCC1=CC2=C(C=C1)OCO2	19.8	1288	1285
**Sesquiterpenes**					
**5281520**	α-Caryophyllene (Humulene)	C/C/1=C\CC(/C=C/C/C(=C/CC1)/C)(C)C	25.2	1454	1456
**5281515**	Caryophyllene	C/C/1=C\CCC(=C)[C@H]2CC([C@@H]2CC1)(C)C	24.1	1420	1417
**1742210**	Caryophyllene oxide	C[C@@]12CC[C@@H]3[C@H](CC3(C)C)C(=C)CC[C@H]1O2	29.1	1584	1582
**289151**	Longifolene	CC1(CCCC2(C3C1C(C2=C)CC3)C)C	23.7	1405	1407
**Other organic compounds**					
**246728**	3-Octanone	CCCCCC(=O)CC	8.6	987	979
**240**	Benzaldehyde	C1=CC=C(C=C1)C=O	7.6	958	952
**2345**	Benzyl benzoate	C1=CC=C(C=C1)COC(=O)C2=CC=CC=C2	34.1	1765	1759
**Compounds searched but non-identified in the three EOs**					
**79035**	Tricyclene	CC1(C2CC3C1(C3C2)C)C	6.2	921	921
**17868**	α-Thujene	CC1=CCC2(C1C2)C(C)C	6.4	926	924
**18818**	Sabinene	CC(C)C12CCC(=C)C1C2	8.1	972	969
**26049**	3-Carene	CC1=CCC2C(C1)C2(C)C	9.4	1010	1008
**7462**	α-terpinene	CC1=CC=C(CC1)C(C)C	9.7	1016	1014
**91496**	Limonene oxide	CC(=C)C1CCC2(C(C1)O2)C	14.3	1138	1137
**6321405**	iso-Borneol	C[C@@]12CC[C@@H](C1(C)C)C[C@H]2O	15.0	1156	1157
**14529**	p-Cymen-8-ol	CC1=CC=C(C=C1)C(C)(C)O	16.1	1185	1179

[a] *RT: retention time (min).* [b] *RI Calculated: Linear retention index experimentally determined on an HP-5MS column (30 m x 0.25 mm, 0.251 mμm) according to Van den Dool and Kratz (1963) calculated using a mixture of n-alkanes (C8 – C30, Supelco, Bellefonte, CA).* [c] *RI Literature: Value taken from the NIST 17 library.*

The determination of EO chemical composition was carried out using gas chromatography–mass spectrometry (GC–MS). After dilution in n-hexane each EO (1 µL) was injected at 280°C. A HP-5MS capillary column was used for separation, using Helium as carrier gas. Analysis was made in electron impact (EI) mode (internal ionization source; 70 eV) with a scan range from 29 to 400 m/z, after a solvent delay of 2.5 min. Compounds were identified by comparing the RI reported in libraries with the obtained RI, calculated from a mix of n-alkanes, and also by comparing the obtained mass spectra with libraries with mass spectra of available analytical standards.

Section S3. EOs' Minimum Bactericidal Concentration (MBC) against the tested bacterial species, reported as a percentage value and mg/mL.

		Cinnamomum zeylanicum		*Origanum vulgare*		*Thymus vulgaris*	
**Gram +**		**MBC**	**EtOH***	**MBC**	**EtOH***	**MBC**	**EtOH***
Staphylococcus aureus	%	0.31	3.1	0.16	1.6	0.16	1.6
(ATCC29213)	mg/mL	3.06	25.26	1.53	12.63	1.48	12.63
Staphylococcus epidermidis	%	0.31	3.1	0.16	1.6	0.16	1.6
(RP62A)	mg/mL	3.06	25.26	1.53	12.63	1.48	12.63
Enterococcus faecalis	%	0.31	3.1	0.31	3.1	0.31	3.1
(ATCC 29212)	mg/mL	3.06	25.26	2.97	25.26	2.87	25.26
**Gram –**							
Escherichia coli	%	0.16	1.6	0.08	0.8	0.16	1.6
(ATCC 25922)	mg/mL	1.58	12.63	0.77	6.32	1.48	12.63
Klebsiella pneumoniae	%	0.31	3.1	0.31	3.1	0.62	6.2
(ATCC 700603)	mg/mL	3.06	25.26	2.97	25.26	5.74	48.94
Pseudomonas aeruginosa	%	0.16	1.6	1.25	12.5	1.25	12.5
(ATCC 27853)	mg/mL	1.58	12.63	11.98	98.67	11.58	97.88

*= percentage of EtOH 96% present in the MBC of each EO.

Section S4. From the DrugCentral 2023 website (drugcentral.org) we downloaded the sdf which contained (downloaded on the 10th nov 2023) 4278 molecules; these were first subjected to preliminary filtering based on molecular weight (MW between 100 and 700 u.m.a) and atoms other than B, C, N, O, P, and halogens. The remaining molecules underwent fingerprinting characterization via DataWarrior software and with the tool Self-Organising Map (SOM) we divided the set into 100 cells (10x10 map). From each cell we kept the molecule closer to the cell.

The set of 100 reference molecules from DrugCentral is reported here:

acetanilide, alsactide, atovaquone, barnidipine, benactyzine, benzyl benzoate, betamethasone butyrate, propionate, betamipron, bisbentiamine, buflomedil, butobarbital, butriptyline, canrenone, carazolol, carbocromen, cefalotin, cefradine, cefuroxime pivoxetil, chlorhexidine, chlormezanone, chloropyrilene, chlorpropamide, chromocarb, cianidanol, cibenzoline, cilnidipine, cinalukast, cinepazide, clebopride, clidanac, clocapramine, clofarabine, clofibric acid, clonidine, clorexolone, clotrimazole, cloxazolam, clozapine, crotetamide, cyclopenthiazide, dapiprazole, delorazepam, deserpidine, desoxycortone, dexetimide, diazoxide, dicycloverine, diethazine, diethylstilbestrol, digoxin, dihydroergotamine, diloxanide furoate, dimethyltubocurarinium, dinoprostone, diphenhydramine, dirithromycin, distigmine, dithiazanine, dosulepin, doxazosin, doxefazepam, doxorubicin, droperidol, dropropizine, drotaverine, droxicam, duloxetine, edoxudine, egualen, eletriptan, enalapril, epalrestat, epinephrine, eprozinol, erdosteine, estradiol, etamivan, fenpiverinium, homarylamine, hydroquinidine, lactitol, levodopa, levorphanol, mannitol, mequinol, methamphetamine, norethandrolone, norfloxacin, oxacillin, pheniramine, posatirelin, prenalterol, proglumide, promazine, propipocaine, propylthiouracil, proxyphylline, scopolamine butylbromide, tertatolol, trolnitrate.

Section S5. Procedure to extract data from the website of the ATCC Genome Portal (https://genomes.atcc.org/).

After logging in into website ATCC (note that data download is subjected to the user’s plan), and searching for a genome, in the "View" button, in correspondence to the column "Genomic Data", there is the option “Download the table CSV” under the tab Genome Browser. With a data mining procedure, available as R scripting (*step01_prepare_data_atcc_mapping.R*, available in the github repository), ATCC codes were associated to gene names and UniProt identifiers.

Section S6. Procedure to access data from the KEGG database.

Bacteria names were used in the search form available at the given link (https://www.kegg.jp/brite/br08611), accessed last time in June 2024. All the codes available are listed below, in alphabetic order except the first row of each bacteria which is reserved to the main entry. In the table, the last column reports the success rate for each org_code (as percentage of data for which corresponding UniProt or NCBI identifiers were retrieved). Data were accessed via R scripting (the scripts *step02a_read_kegg_data.R* and *step03_prepare_data_kegg_mapping.R* are both available in the github repository), through the functions *keggList* and *keggGet* of the R package KEGGREST (1.42.0).

**Bacteria**	**Org code**	**T number**	**Nr UniProt**	**Nr NCBI**	**Nr Pathways**	**Success rate (%)**
**Staphylococcus aureus**						
Staphylococcus aureus subsp. aureus COL, methicillin-resistant	sac	T00225	932	932	112	97.4
Staphylococcus aureus subsp. aureus USA300_FPR3757, community-acquired methicillin-resistant	saa	T00323	952	952	112	97.4
S. aureus RF122/ET3-1, bovine mastitis-causing	sab	T00303	380	918	111	97.3
S. aureus subsp. aureus ED98	sad	T01124	1	928	112	97.0
S. aureus subsp. aureus Newman	sae	T00557	937	938	112	97.3
Staphylococcus aureus subsp. aureus JH1, methicillin-resistant, vancomycin-susceptible	sah	T00556	283	950	112	97.2
Staphylococcus aureus subsp. aureus JH9, methicillin-resistant, vancomycin-intermediate resistance	saj	T00522	281	953	115	97.2
Staphylococcus aureus subsp. aureus MW2, community-acquired methicillin-resistant	sam	T00086	945	946	112	97.1
S. aureus MS4	sams	T04800	793	879	112	97.3
S. aureus subsp. aureus NCTC 8325	sao	T00324	927	928	112	97.3
Staphylococcus aureus subsp. aureus MRSA252, methicillin-resistant	sar	T00182	927	927	111	97.3
Staphylococcus aureus subsp. aureus MSSA476, methicillin-susceptible	sas	T00183	528	935	112	97.8
Staphylococcus aureus subsp. aureus N315, hospital-acquired methicillin-resistant, vancomycin-susceptible	sau	T00051	949	950	112	97.1
S. aureus subsp. aureus 55/2053	saua	T02810	925	925	112	97.6
S. aureus 08BA02176, livestock-associated methicillin-resistant	saub	T02339	0	917	112	97.3
S. aureus CA-347, methicillin-resistant	sauc	T02702	0	944	112	97.3
S. aureus 502A	saud	T03491	908	933	112	97.0
S. aureus subsp. aureus 6850, methicillin-sensitive	saue	T02825	0	926	112	96.7
S. aureus subsp. aureus FDAARGOS_5	sauf	T03492	0	942	112	97.4
S. aureus subsp. aureus SA268, community-associated methicillin-resistant	saug	T03790	0	935	112	97.2
S. aureus USA300-ISMMS1, methicillin-resistant	saui	T03115	0	958	112	97.3
S. aureus subsp. aureus ST228/10497, methicillin-resistant	sauj	T03144	821	826	110	96.5
S. aureus subsp. aureus ST228/15532, methicillin-resistant	sauk	T03145	821	827	110	96.5
S. aureus M1, methicillin-resistant	saum	T02618	0	947	111	97.8
S. aureus subsp. aureus CN1, community-associated methicillin-resistant	saun	T02826	0	937	112	97.6
S. aureus subsp. aureus ST228/16035, methicillin-resistant	sauq	T03146	822	827	110	96.5
S. aureus Bmb9393, methicillin-resistant	saur	T02717	0	949	112	97.3
S. aureus subsp. aureus SA40, community-associated methicillin-resistant	saus	T02849	0	922	111	97.1
S. aureus subsp. aureus ST228/10388, methicillin-resistant	saut	T03143	822	827	110	96.5
S. aureus subsp. aureus SA957, community-associated methicillin-resistant	sauu	T02850	0	925	111	97.2
S. aureus subsp. aureus ST228/18412, methicillin-resistant	sauv	T03147	826	826	110	96.6
S. aureus subsp. aureus ST228/16125, methicillin-resistant	sauw	T03148	818	824	110	96.7
S. aureus subsp. aureus ST228/18341, methicillin-resistant	saux	T03149	822	827	110	96.7
S. aureus subsp. aureus ST228/18583, methicillin-resistant	sauy	T03150	822	825	110	96.6

Bacteria	Org code	T number	Nr UniProt	Nr NCBI	Nr Pathways	Success rate (%)
S. aureus subsp. aureus Z172, methicillin-resistant, vancomycin-intermediate resistance	sauz	T02886	0	954	112	97.4
Staphylococcus aureus subsp. aureus Mu50, methicillin-resistant, vancomycin-intermediate resistance	sav	T00052	950	951	112	97.3
Staphylococcus aureus subsp. aureus Mu3, methicillin-resistant, heterogeneous vancomycin-intermediate resistance	saw	T00588	299	950	112	97.2
Staphylococcus aureus subsp. aureus USA300_TCH1516, community-acquired methicillin-resistant	sax	T00629	262	912	112	96.9
S. aureus subsp. aureus ECT-R 2, methicillin-sensitive	suc	T01887	0	942	115	97.1
S. aureus subsp. aureus 71193, methicillin-sensitive	sud	T02043	0	920	112	97.4
S. aureus subsp. aureus ED133	sue	T01869	0	929	112	97.3
S. aureus subsp. aureus LGA251, methicillin-resistant	suf	T02085	0	933	112	97.1
S. aureus subsp. aureus ST398, methicillin-resistant	sug	T02071	1	912	111	97.9
S. aureus subsp. aureus JKD6159, community-acquired methicillin-resistant	suj	T01870	0	935	112	97.1
S. aureus subsp. aureus JKD6008, methicillin-resistant, vancomycin-intermediate resistance	suk	T01886	0	929	112	96.5
S. aureus subsp. aureus TCH60	suq	T01889	0	941	112	96.8
S. aureus subsp. aureus T0131, methicillin-resistant	sut	T01888	0	940	111	97.4
S. aureus subsp. aureus M013, community-acquired methicillin-resistant	suu	T01744	0	930	112	97.1
S. aureus subsp. aureus VC40	suv	T01745	0	930	112	97.0
Staphylococcus aureus subsp. aureus TW20, methicillin-resistant	suw	T02060	1	955	115	96.4
S. aureus subsp. aureus HO 5096 0412, methicillin-resistant	sux	T02059	0	924	111	95.8
S. aureus 04-02981, methicillin-resistant	suy	T02070	3	950	112	97.0
S. aureus subsp. aureus 11819-97, community-acquired methicillin-resistant	suz	T01950	0	949	112	97.3
**Staphylococcus epidermidis**						
Staphylococcus epidermidis RP62A, methicillin-resistant	ser	T00229	854	857	105	96.9
Staphylococcus epidermidis ATCC 12228	sep	T00110	866	868	105	97.0
Staphylococcus epidermidis PM221	sepp	T03289	0	857	105	97.5
Staphylococcus epidermidis SEI	seps	T03343	484	845	105	96.8
**Enterococcus faecalis**						
Enterococcus faecalis ATCC 29212	efq	T03320	1	916	111	97.5
Enterococcus faecalis V583	efa	T00123	930	931	110	97.7
Enterococcus faecalis D32	efd	T02172	0	910	112	97.6
Enterococcus faecalis OG1RF	efi	T01882	7	899	111	97.0
Enterococcus faecalis 62	efl	T01881	0	942	112	97.8
Enterococcus faecalis DENG1	efn	T03043	0	918	112	97.5
Enterococcus faecalis Symbioflor 1	efs	T02399	0	894	112	97.3
Enterococcus faecalis 7L76	ene	T02585	400	727	107	97.7
**Escherichia coli**						
Escherichia coli K-12 MG1655	eco	T00007	1625	1629	130	96.0
Escherichia coli ABU 83972	eab	T01995	0	1735	131	97.8
Escherichia coli BL21-Gold(DE3)pLysS AG	ebd	T00939	1631	1631	131	97.5
Escherichia coli BL21(DE3)	ebe	T02096	8	1619	131	97.6
Escherichia coli BL21(DE3)	ebl	T00931	8	1621	131	96.1
Escherichia coli B REL606	ebr	T00944	7	1615	130	97.6
Escherichia coli BW2952 (K-12 MC41000 with placMu50 fusion)	ebw	T00913	430	1617	130	96.0
Escherichia coli O6:K2:H1 CFT073 (UPEC)	ecc	T00106	1729	1729	131	97.9
Escherichia coli K-12 DH10B	ecd	T00666	443	1608	128	96.4
Escherichia coli O157:H7 EDL933 (EHEC)	ece	T00044	1736	1747	131	97.7
Escherichia coli O157:H7 EC4115 (EHEC)	ecf	T00778	438	1732	131	96.9
Escherichia coli O127:H6 E2348/69 (EPEC)	ecg	T00796	1660	1662	129	96.3
Escherichia coli O18:K1:H7 UTI89 (UPEC)	eci	T00338	1739	1739	129	97.9
Escherichia coli K-12 W3110	ecj	T00068	1632	1632	130	96.5

Bacteria	Org code	T number	Nr UniProt	Nr NCBI	Nr Pathways	Success rate (%)
Escherichia coli 55989 (EAEC)	eck	T00826	1699	1699	131	95.6
Escherichia coli ATCC 8739	ecl	T00697	480	1696	130	97.1
Escherichia coli SMS-3-5 (environmental isolate)	ecm	T00672	1729	1729	132	97.8
Escherichia coli APEC O78 (APEC)	ecoa	T02445	0	1682	131	97.7
Escherichia coli SHuffle B C3029	ecob	T09610	0	1609	131	97.4
Escherichia coli SHuffle K-12 C3026	ecoc	T09611	0	1698	130	97.4
Escherichia coli O145:H28 RM13516 (EHEC)	ecoh	T03011	0	1782	133	97.8
Escherichia coli O18:K1 PMV-1 (ExPEC)	ecoi	T02847	0	1745	128	94.6
Escherichia coli JJ1886	ecoj	T02896	0	1798	132	97.9
Escherichia coli K-12 MDS42	ecok	T02541	0	1449	129	96.0
Escherichia coli LY180	ecol	T02846	0	1674	130	97.7
Escherichia coli O145:H28 RM13514 (EHEC)	ecoo	T03010	0	1779	133	97.8
Escherichia coli O25b:K100:H4-ST131 EC958 (UPEC)	ecos	T04373	1789	1789	132	97.8
Escherichia coli O6:K15:H31 536 (UPEC)	ecp	T00373	1690	1691	129	97.9
Escherichia coli O81 D1a (commensal strain)	ecq	T00827	1691	1694	130	96.2
Escherichia coli O8 IAI1 (commensal strain)	ecr	T00828	448	1675	130	95.8
Escherichia coli O157:H7 Sakai (EHEC)	ecs	T00048	1716	1742	131	97.7
Escherichia coli O7:K1 IAI39 (ExPEC)	ect	T00829	1701	1703	131	95.9
Escherichia coli APEC O1 (Avian pathogenic Escherichia coli)	ecv	T00425	1692	1693	130	98.1
Escherichia coli O139:H28 E24377A (ETEC)	ecw	T00590	1651	1651	131	97.9
Escherichia coli O9 HS (commensal strain)	ecx	T00591	1615	1615	130	96.6
Escherichia coli O152:H28 SE11 (commensal strain)	ecy	T00784	1706	1713	131	97.9
Escherichia coli O45:K1:H7 S88 (ExPEC)	ecz	T00830	0	1738	128	96.1
Escherichia coli DH1	edh	T01996	5	1634	134	97.4
Escherichia coli DH1	edj	T02102	0	1623	130	96.6
Escherichia coli O18:K1:H7 IHE3034 (ExPEC)	eih	T01997	0	1735	129	97.6
Escherichia coli KO11FL	ekf	T02104	0	1738	130	97.8
Escherichia coli KO11FL	eko	T01718	0	1705	130	97.7
Escherichia coli clone D i14	elc	T02003	0	1718	131	97.8
Escherichia coli clone D i2	eld	T02004	0	1718	131	97.8
Escherichia coli LF82	elf	T00854	0	1709	129	96.5
Escherichia coli O78:H11:K80 H10407 (ETEC)	elh	T02069	2	1680	130	97.8
Escherichia coli W	ell	T02101	4	1707	130	97.8
Escherichia coli O83:H1 NRG 857C (AIEC)	eln	T02068	0	1733	130	97.7
Escherichia coli O44:H18 042 (EAEC)	elo	T02067	0	1711	130	97.8
Escherichia coli P12b	elp	T02030	0	1638	131	97.7
Escherichia coli O55:H7 RM12579 (EPEC)	elr	T02066	0	1746	130	97.6
Escherichia coli UM146	elu	T02000	0	1727	129	97.8
Escherichia coli W	elw	T02002	309	1689	130	96.3
Escherichia coli O157:H7 Xuzhou21 (EHEC)	elx	T02122	0	1750	131	97.8
Escherichia coli NA114 (UPEC)	ena	T01998	0	1736	132	98.0
Escherichia coli O7:K1 CE10	eoc	T01999	0	1751	131	97.9
Escherichia coli O103:H2 12009 (EHEC)	eoh	T01096	5	1759	132	96.6
Escherichia coli O111:H- 11128 (EHEC)	eoi	T01097	6	1771	132	96.6
Escherichia coli O26:H11 11368 (EHEC)	eoj	T01098	5	1847	132	96.8
Escherichia coli O55:H7 CB9615 (atypical EPEC)	eok	T01190	7	1755	131	96.8
Escherichia coli O150:H5 SE15 (commensal)	ese	T02077	0	1716	131	97.7
Escherichia coli O104:H4 2011C-3493 (EAEC)	esl	T02257	1717	1719	132	97.8
Escherichia coli O104:H4 2009EL-2050 (EAEC)	esm	T02316	0	1719	132	97.7
Escherichia coli O104:H4 2009EL-2071 (EAEC)	eso	T02256	1	1716	132	97.7
Escherichia coli O157:H7 TW14359 (EHEC)	etw	T00949	7	1740	131	97.7
Escherichia coli O17:K52:H18 UMN026 (ExPEC)	eum	T00831	1788	1789	131	96.3
Escherichia coli UMNK88 (ETEC, porcine)	eun	T02001	0	1708	131	98.0
**Klebsiella pneumoniae**						
Klebsiella pneumoniae subsp. pneumoniae MGH 78578 (serotype K52)	kpn	T00566	1867	1870	128	97.9
Klebsiella pneumoniae 30660/NJST258_1	kpa	T03176	1830	1832	128	98.3
Klebsiella pneumoniae blaNDM-1	kpb	T03440	1788	1846	128	97.7

Bacteria	Org code	T number	Nr UniProt	Nr NCBI	Nr Pathways	Success rate (%)
Klebsiella pneumoniae subsp. pneumoniae KPNIH10	kpc	T03788	0	1865	128	97.9
Klebsiella pneumoniae subsp. pneumoniae KPNIH32	kpg	T03468	1821	1916	129	98.0
Klebsiella pneumoniae subsp. pneumoniae KPNIH24	kph	T03369	0	1863	127	97.9
Klebsiella pneumoniae CG43	kpi	T02866	0	1823	129	97.9
Klebsiella pneumoniae JM45	kpj	T02799	0	1782	127	97.9
Klebsiella pneumoniae subsp. pneumoniae HS11286	kpm	T01733	1867	1870	127	98.5
Klebsiella pneumoniae 32192	kpne	T03746	1831	1848	128	97.9
Klebsiella pneumoniae 34618	kpnu	T03747	1264	1915	131	98.0
Klebsiella pneumoniae Kp52.145	kpnk	T05281	1826	1868	128	98.0
Klebsiella pneumoniae KCTC 2242	kpo	T01982	0	1885	130	97.9
Klebsiella pneumoniae subsp. pneumoniae 1084 (serotype K1)	kpp	T02213	0	1865	128	97.9
Klebsiella pneumoniae subsp. pneumoniae KPR0928	kpq	T03371	0	1830	128	97.8
Klebsiella pneumoniae subsp. rhinoscleromatis SB3432	kpr	T02793	0	1738	128	98.3
Klebsiella pneumoniae 30684/NJST258_2	kps	T03177	1838	1838	128	98.1
Klebsiella pneumoniae subsp. pneumoniae ATCC 43816 KPPR1	kpt	T03418	931	1873	128	97.9
Klebsiella pneumoniae subsp. pneumoniae NTUH-K2044 (serotype K1)	kpu	T00910	9	1906	128	97.7
Klebsiella pneumoniae subsp. pneumoniae KPNIH29	kpv	T03465	1157	1856	127	97.9
Klebsiella pneumoniae subsp. pneumoniae KPNIH30	kpw	T03466	1763	1841	127	97.9
Klebsiella pneumoniae PMK1	kpx	T03419	1864	1895	128	98.0
Klebsiella pneumoniae subsp. pneumoniae KPNIH31	kpy	T03467	1715	1836	128	97.9
Klebsiella pneumoniae subsp. pneumoniae KPNIH27	kpz	T03370	0	1860	127	98.0
**Pseudomonas aeruginosa**						
Pseudomonas aeruginosa PAO1	pae	T00035	1929	1941	131	98.1
Pseudomonas aeruginosa NCGM 1900	paeb	T03789	1	1942	132	98.7
Pseudomonas aeruginosa c7447m	paec	T03098	0	1938	132	98.7
Pseudomonas aeruginosa YL84	paeg	T03035	0	1935	131	98.7
Pseudomonas aeruginosa PAO1-VE2	paei	T03170	0	1942	131	98.7
Pseudomonas aeruginosa LES431	pael	T02970	0	1923	133	98.7
Pseudomonas aeruginosa MTB-1	paem	T02951	0	1966	132	98.8
Pseudomonas aeruginosa PA1	paep	T02928	0	1949	132	98.7
Pseudomonas aeruginosa PA1R	paer	T02929	0	1886	130	98.6
Pseudomonas aeruginosa SCV20265	paes	T02971	0	1962	132	98.8
Pseudomonas aeruginosa PA38182	paeu	T03031	0	1970	131	98.9
Pseudomonas aeruginosa PAO1-VE13	paev	T03097	0	1942	131	98.7
Pseudomonas aeruginosa M18	paf	T01973	0	1942	133	98.6
Pseudomonas aeruginosa LESB58	pag	T00818	325	1924	133	98.3
Pseudomonas aeruginosa PA7	pap	T00569	1913	1918	131	98.8
Pseudomonas aeruginosa UCBPP-PA14	pau	T00401	1955	1959	131	98.8
Pseudomonas aeruginosa DK2	pdk	T02161	0	1908	132	98.7
Pseudomonas aeruginosa NCGM2.S1	pnc	T01974	0	1953	132	98.6
Pseudomonas aeruginosa RP73	prp	T02711	0	1906	131	98.7
Pseudomonas aeruginosa B136-33	psg	T02627	0	1957	132	98.7
Pseudomonas aeruginosa B18	sech	T06681	0	1949	132	98.7

Section S7. Procedure to access data to link PDB entries to UniProt codes and gene names.

Data were downloaded from UniProt website via ftp, through the following link:

https://ftp.uniprot.org/pub/databases/uniprot/current_release/knowledgebase/complete/docs/pdbtosp.txt

With a data mining procedure, available as R scripting (*step04_prepare_data_genes_mapping.R*, available in the github repository), PDB entries were associated to gene names and UniProt identifiers.

Section S8 (Complementary to Figure 3 of the paper). Bar plots of the number of targets identified for each molecule; data are reported *E. faecalis* and *K. pneumoniae*, for which the overall amount was very low.

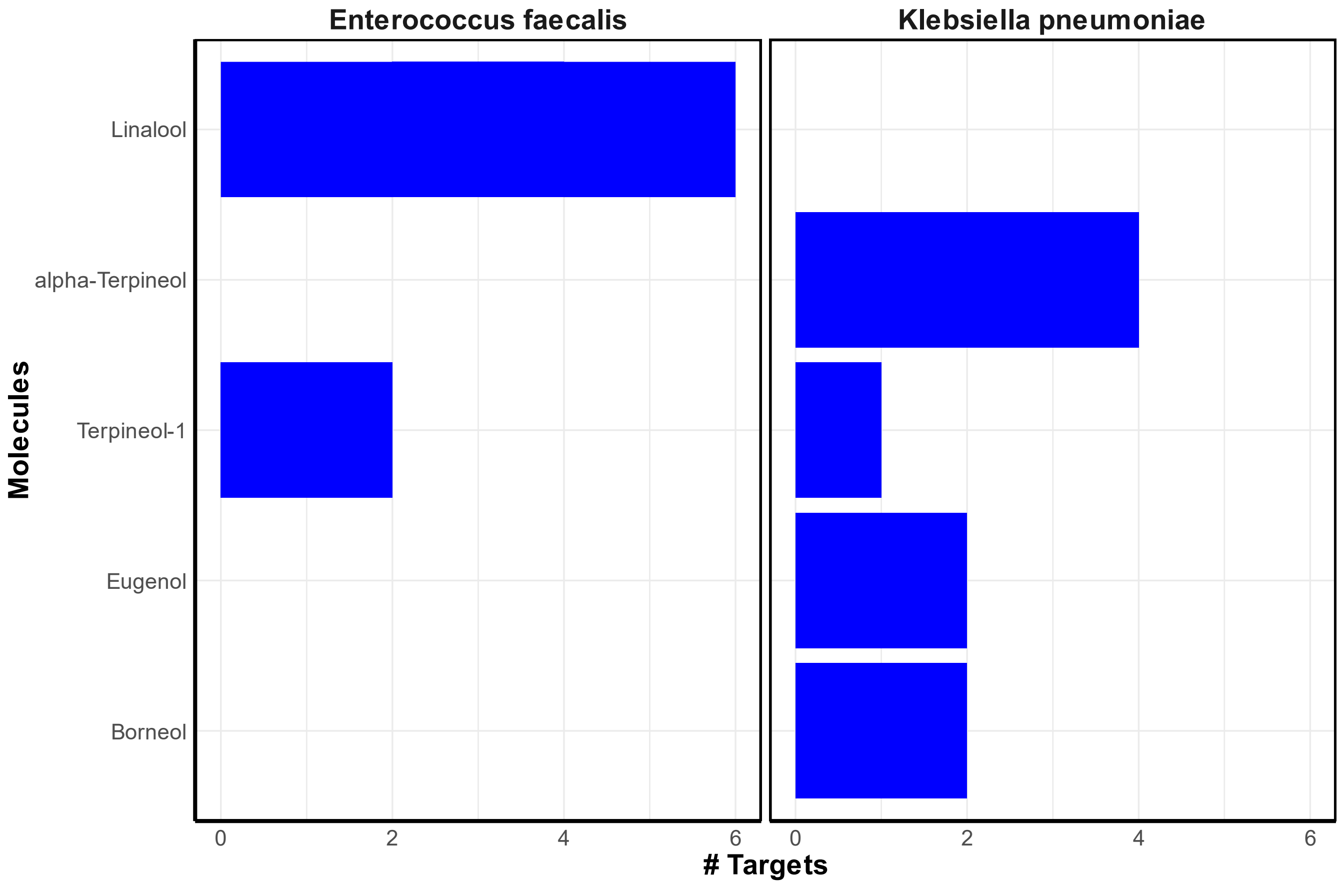

Section S9. Manual curation of the genes on ATCC strains for different bacteria. Manual curation on the ATCC website (available at https://genomes.atcc.org/genomes) was done with the options *Search*, *View* and *Genome Browser*. Unsuccessful searches were further refined through the UniProt website, by using the Search prompt available (https://www.uniprot.org). The same website was used to extract the EC code for each protein. Most of the genes were found in the genome of the corresponding ATCC code, and for most of the genes the corresponding EC code were found. Manual curation was successful for most of the genes, with a few exceptions: 7/26 genes for *S. aureus*, 11/200 for *E. coli* and 11/59 for *P. aeruginosa*. Targets involved in significant KEGG pathways are reported in bold, with the code to be retrieved in the pathways through the KEGG website. The manual curation on the KEGG website was carried out at https://www.genome.jp/kegg/pathway.html For all the bacterium-pathways pairs, the corresponding codes (org_code and pathway name) were used in the forms "Select prefix" and "Enter keywords", respectively.

In the tables below, the column "Code in KEGG" reports the corresponding codes for the listed genes; in the case of *E. coli*, 16/169 genes were not retrieved in the pathways ("Not found"), but most of the times the reason was multiple names due to protein complexes or different protein subunits. The only exceptions were NrdI and NagD for E coli and UbiX and PhnW for *P. aeruginosa*, for which we were not able to retrieve in the KEGG pathways through the website. Notably, the collected data reported below for the three bacteria with significant pathways, are subjected to webserver updates and are not considered by the authors as a substitution of the original website content, but as a tool to easily access and compare relevant information.

S. aureus. (ATCC® 29213™).

Gene	Curation	Code in KEGG	EC code	Protein
ACCA	accA		2.1.3.15	Acetyl-coenzyme A carboxylase carboxyl transferase subunit alpha
ACCD	accD		2.1.3.15	Acetyl-coenzyme A carboxylase carboxyl transferase subunit beta
CLPP	clpP		3.4.21.92	ATP-dependent Clp endopeptidase proteolytic subunit ClpP
**COAD**	**coaD** (alternate name for kdtB)	SACOL1134	2.7.7.3	Phosphopantetheine adenylyltransferase
**COAW**	**coaW**	SACOL2122	2.7.1.33	Type II pantothenate kinase
CRTM	crtM		2.5.1.96	4,4'-diapophytoene synthase CrtM
DDL	Not found		6.3.2.4	D-alanine-D-alanine ligase
ETB	Not found		-	
FABI	fabI		1.3.1.-	enoyl-ACP reductase FabI
FEMX	femX		2.3.2.16	lipid II:glycine glycyltransferase
G3P1	sap		-	type I glyceraldehyde-3-phosphate dehydrogenase
HIS7	hisB		4.2.1.19	imidazoleglycerol-phosphate dehydratase HisB
HLA	Hyl		-	alpha-hemolysin
**ILVC**	**ilvC**	SACOL2045	1.1.1.86	ketol-acid reductoisomerase
ISDA	isdA		-	LPXTG-anchored heme-scavenging protein IsdA
LUKDV	lukD		-	bi-component leukocidin LukED subunit D
MAZF	Not found		3.1.-.-	type II toxin-antitoxin system PemK/MazF family toxin
MOAA	moaA		4.1.99.22	GTP 3',8-cyclase MoaA
NANA	Not found		4.3.1.1	N-acetylneuraminate lyase
NOSO	Not found		-	nitric oxide synthase oxygenase
**PANC**	**panC**	SACOL2614	6.3.2.1	pantoate--beta-alanine ligase
SARR	sarR		-	HTH-type transcriptional regulator SarR
SPA	spa		-	staphylococcal protein A
T2S3	Not found		-	
TPIS	tpiA		5.3.1.1	triose-phosphate isomerase
Y1800	Not found		-	

E. coli (ATCC® 25922™).

Gene	Curation	Code in KEGG	EC code	Protein
**5DNU**	**yfbR**	b2291	3.1.3.89	5'-deoxynucleotidase
**6PGD**	**gnd**	b2029	1.1.1.44	6-phosphogluconate dehydrogenase, decarboxylating
**AAT**	**aspC**	b0928	2.6.1.1	aspartate aminotransferase
**ABDH**	**patD**	b1444	1.2.1.19	gamma-aminobutyraldehyde dehydrogenase
**ACCC**	**accC**	b3256	6.3.4.14	biotin carboxylase
**ACEA**	**aceA**	b4015	4.1.3.1	isocitrate lyase
**ACNB**	**acnB**	b0118	4.2.1.3	Aconitate hydratase B
ACP	acpP	Not found		
**ACRB**	**acrB**	b0462	-	multidrug efflux pump subunit AcrB
**ADHE**	**adhE**	b1241	1.1.1.1, 1.2.1.10	bifunctional aldehyde-alcohol dehydrogenase
**ADIA**	**adiA**	b4117	4.1.1.19	biodegradative arginine decarboxylase
**ALAA**	**alaA**	b2290	2.6.1.2	glutamate-pyruvate aminotransferase AlaA
**ALDA**	**aldA**	b1415	1.2.1.21, 1.2.1.22	aldehyde dehydrogenase
**ALF**	**fbaA**	b2925	4.1.2.13	fructose-bisphosphate aldolase class II
**ALKH**	**eda**	b1850	4.1.2.14, 4.1.3.42	KHG/KDPG aldolase
**AMO**	**tynA**	b1386	1.4.3.21	primary amine oxidase
**AMPA**	**pepA**	b4260	3.4.11.1	cytosol aminopeptidase
**AMPC**	**ampC**	b4150	3.5.2.6	beta-lactamase
**AMPN**	**pepN**	b0932	3.4.11.2	aminopeptidase N
AMPP	pepP		3.4.11.9	Xaa-Pro aminopeptidase
AMTB	amtB		-	ammonium transporter AmtB
ARAA	araA		5.3.1.4	L-arabinose isomerase
ARGB	argB		2.7.2.8	acetylglutamate kinase
ASCG	Not found			
**ASNB**	**asnB**	b0674	6.3.5.4	asparagine synthetase B
**ASPG1**	**ansA**	b1767	3.5.1.1	L-asparaginase 1
**ASPG2**	**ansB**	b2957	3.5.1.1	L-asparaginase 2
**ASSY**	**argG**	b3172	6.3.4.5	argininosuccinate synthase
**ASTB**	**astB**	b1745	3.5.3.23	N-succinylarginine dihydrolase
**ASTC**	**astC**	b1748	2.6.1.81	succinylornithine transaminase
**ATDA**	**speG**	b1584	2.3.1.57	spermidine N-acetyltransferase
**ATOB**	**atoB**	b2224	2.3.1.9	acetyl-CoA acetyltransferase
**BCCP**	**accB**	b3255	-	biotin carboxyl carrier protein of acetyl-CoA carboxylase
**BFR**	**bfr**	b3336	1.16.3.1	bacterioferritin
**BGAL**	**lacZ**	b0344	3.2.1.23	beta-galactosidase
**BGLR**	**uidA**	b1617	3.2.1.31	beta-glucuronidase
**BLAT**	**bla**	Not found		
CADC	cadC		-	lysine decarboxylation/transport transcriptional activator CadC
**CAN**	**can**	b0126	4.2.1.1	carbonic anhydrase 2
**CAPP**	**ppc**	b3956	4.1.1.31	phosphoenolpyruvate carboxylase
**CARA**	**carA**	b0032	6.3.5.5	carbamoyl-phosphate synthase small chain
**CARB**	**carB**	b0033	6.3.5.5	carbamoyl-phosphate synthase large chain
**CATE**	**katE**	b1732	1.11.1.6	catalase HPII
**CDD**	**cdd**	b2143	3.5.4.5	cytidine deaminase
**CHEA**	**cheA**	b1888	2.7.13.3	chemotaxis protein CheA
**CHEY**	**cheY**	b1882	-	chemotaxis protein CheY
CLPA	clpA	Not found		
CLPS	clpS	Not found		
CLPP	clpP		3.4.21.92	ATP-dependent Clp endopeptidase proteolytic subunit ClpP
COAD	coaD		2.7.7.3	pantetheine-phosphate adenylyltransferase
**CISY**	**gltA**	b0720	2.3.3.1	citrate synthase
**CODA**	**codA**	b0337	3.5.4.1	cytosine/isoguanine deaminase
CPOB	cpoB		-	cell division protein CpoB
CRP	crp		-	cAMP-activated global transcriptional regulator CRP
**CYBH**	**hyaC**	Not found		
**CYNS**	**cynS**	b0340	4.2.1.104	cyanate hydratase

Gene	Curation	Code in KEGG	EC code	Protein
**DAPA**	**dapA**	b2478	4.3.3.7	4-hydroxy-tetrahydrodipicolinate synthase
**DAPB**	**dapB**	b0031	1.17.1.8	4-hydroxy-tetrahydrodipicolinate reductase
DAUA	dauA	Not found		
**DCD**	**dcd**	b2065	3.5.4.13	dCTP deaminase
**DCEA**	**gadA**	b3517	4.1.1.15	glutamate decarboxylase alpha
**DCEB**	**gadB**	b1493	4.1.1.15	glutamate decarboxylase beta
**DEOC**	**deoC**	b4381	4.1.2.4	deoxyribose-phosphate aldolase
**DHAS**	**asd**	b3433	1.2.1.11	aspartate-semialdehyde dehydrogenase (ASA dehydrogenase)
**DHE4**	**gdhA**	b1761	1.4.1.4	glutamate dehydrogenase
**DHSC**	**sdhC**	b0721	-	succinate dehydrogenase cytochrome b subunit
**DHSD**	**sdhD**	b0722	-	succinate dehydrogenase hydrophobic membrane anchor subunit
**DPS**	**dps**	Not found		
**DUT**	**dut**	b3640	3.6.1.23	dUTP diphosphatase
**FABA**	**fabA**	b0954	4.2.1.59	3-hydroxydecanoyl-ACP dehydratase
**FABB**	**fabB**	b2323	2.3.1.41	3-oxoacyl-ACP synthase 1
**FABD**	**fabD**	b1092	2.3.1.39	malonyl CoA-acyl carrier protein transacylase
**FABF**	**fabF**	b1095	2.3.1.179	3-oxoacyl-ACP synthase 2
**FABH**	**fabH**	b1091	2.3.1.180	beta-ketoacyl-ACP synthase III
**FABI**	**fabI**	b1288	1.3.1.9	enoyl-ACP reductase
**FABZ**	**fabZ**	b0180	4.2.1.59	3-hydroxyacyl-ACP dehydratase
**FCTA**	**frc**	b2374	2.8.3.16	formyl-CoA transferase
**FDHF**	**fdhF**	b4079	1.17.98.4	formate dehydrogenase H
**FLIM**	**fliM**	b1945	-	flagellar motor switch protein FliM
**FOLD**	**folD**	b0529	1.5.1.5	bifunctional methylenetetrahydrofolate dehydrogenase/cyclohydrolase
**FTSI**	**ftsI**	b0084	3.4.16.4	peptidoglycan DD-transpeptidase FtsI
FUCI	fucI		5.3.1.25	L-fucose isomerase
**FUCO**	**fucO**	b2799	1.1.1.77	lactaldehyde reductase
**FUMC**	**fumC**	b1611	4.2.1.2	fumarate hydratase
**G6PI**	**pgi**	b4025	5.3.1.9	glucose-6-phosphate isomerase
**GABD**	**gabD**	b2661	1.2.1.16, 1.2.1.79	succinate-semialdehyde dehydrogenase [NADP(+)]
**GABT**	**gabT**	b2662	1.2.1.19	4-aminobutyrate aminotransferase
**GCSH**	**gcvH**	b2904	-	glycine cleavage system H protein
**GCST**	**gcvT**	b2905	2.1.2.10	aminomethyltransferase
**GGT**	**ggt**	b3447	2.3.2.2	glutathione hydrolase proenzyme
**GHRA**	**ghrA**	b1033	1.1.1.79, 1.1.1.81	glyoxylate/hydroxypyruvate reductase A
**GLMS**	**glmS**	b3729	2.6.1.16	glutamine-fructose-6-phosphate aminotransferase
GLPG	glpG		3.4.21.105	rhomboid family intramembrane serine protease GlpG
**GLSA1**	**glsA**	b0485	3.5.1.2	glutaminase 1
**GSH1**	**gshA**	b2688	6.3.2.2	glutamate-cysteine ligase
**GSP**	**gss**	b2988	3.5.1.78, 6.3.1.8	bifunctional glutathionylspermidine synthetase/amidase
**HEM2**	**hemB**	b0369	4.2.1.24	Delta-aminolevulinic acid dehydratase / porphobilinogen synthase
**HIS8**	**hisC**	b2021	2.6.1.9	histidinol-phosphate aminotransferase
HPPK	folK		2.7.6.3	2-amino-4-hydroxy-6- hydroxymethyldihydropteridine diphosphokinase
**IAAA**	**iaaA**	b0828	3.5.1.1, 3.4.19.5	isoaspartyl peptidase
**IDH**	**icd**	b1136	1.1.1.42	isocitrate dehydrogenase
**ILVE**	**ilvE**	b3770	2.6.1.42	branched-chain-amino-acid aminotransferase
**ILVN**	**ilvN**	b3670	2.2.1.6	acetolactate synthase isozyme 1 small subunit
KDSC	kdsC		3.1.3.45	3-deoxy-manno-octulosonate-8-phosphatase KdsC
LDCI	cadA		4.1.1.18	lysine decarboxylase CadA
**MALE**	**malE**	b4034	7.5.2.1	maltose/maltodextrin-binding periplasmic protein
**MALF**	**malF**	Not found		
**MALG**	**malG**	Not found		
**MALK**	**malK**	Not found		
**MALY**	**malY**	b1622	4.4.1.13	protein MalY
**MASY**	**aceB**	b4014	2.3.3.9	malate synthase A

Gene	Curation	Code in KEGG	EC code	Protein
**MBHM**	**hybC**	b2994	1.12.99.6	hydrogenase 2 large subunit
**MBHL**	**hyaB**	b0973	1.12.99.6	hydrogenase 1 large subunit
**MBHS**	**hyaA**	b0972	1.12.99.6	hydrogenase 1 small subunit
**MBHT**	**hybO**	b2997	1.12.99.6	hydrogenase 2 small subunit
MCBA	mcbA		-	DUF1471 family periplasmic protein McbA
MCBB	Not found			
MCBC	Not found			
MCBD	Not found			
MCCF	Not found			
**MDH**	**mdh**	b3236	1.1.1.37	malate dehydrogenase
**METB**	**metB**	b3939	2.5.1.48	cystathionine gamma-synthase
**METC**	**metC**	b3008	4.4.1.13	cystathionine beta-lyase
**METF**	**metF**	b3941	1.5.1.54	5,10-methylenetetrahydrofolate reductase
**METH**	**metH**	b4019	2.1.1.13	methionine synthase
**MGLB**	**mglB**	b2150	-	D-galactose/methyl-galactoside binding periplasmic protein MglB
**MGSA**	**mgsA**	b0963	4.2.3.3	methylglyoxal synthase
**MRDA**	**mrdA**	b0635	-	peptidoglycan D,D-transpeptidase MrdA
MURE	Not found			
MUTS	mutS	Not found		
**NADB**	**nadB**	b2574	1.4.3.16	L-aspartate oxidase
NAGD	nagD	Not found		
**NAPA**	**napA**	b2206	1.9.6.1	periplasmic nitrate reductase
**NARG**	**narG**	b1224	1.7.5.1	nitrate reductase subunit alpha
**NARH**	**narH**	b1225	1.7.5.1	nitrate reductase subunit beta
**NARI**	**narI**	Not found	-	respiratory nitrate reductase subunit gamma
**NARK**	**narK**	b1223	-	nitrate/nitrite antiporter
**NFSA**	**nfsA**	b0851	-	Oxygen-insensitive NADPH nitroreductase
**NRDI**	**nrdI**	Not found		
**NRFA**	**nrfA**	b4070	1.7.2.2	cytochrome c552 nitrite reductase
**ODO2**	**sucB**	b0727	2.3.1.61	dihydrolipoyllysine-residue succinyltransferase component of 2-oxoglutarate dehydrogenase complex
**ODP1**	**aceE**	b0114	1.2.4.1	pyruvate dehydrogenase E1 component
**OMPC**	**ompC**	b2215	-	outer membrane porin C
**OMPF**	**ompF**	b0929	-	outer membrane porin F
**OPPA**	**oppA**	b1243	-	periplasmic oligopeptide-binding protein OppA
**OXC**	**oxc**	b2373	4.1.1.8	oxalyl-CoA decarboxylase
PAAA	Not found			
PAAC	Not found			
**PAAH**	**paaH**	b1395	1.1.1.157	3-hydroxyadipyl-CoA dehydrogenase
PAC	Not found			
PADL	Not found			
**PAND**	**panD**	b0131	4.1.1.11	aspartate 1-decarboxylase
**PANZ**	**panZ**	Not found		
**PAT**	**patA**	b3073	2.6.1.82	putrescine aminotransferase
PCKA	pckA			phosphoenolpyruvate carboxykinase (ATP)
PDXJ	pdxJ		2.6.99.2	pyridoxine 5'-phosphate synthase
**PEPB**	**pepB**	b2523	3.4.11.23	peptidase B
**PFKA**	**pfkA**	b3916	2.7.1.11	6-phosphofructokinase 1
**PFKB**	**pfkB**	b1723	2.7.1.11	6-phosphofructokinase 2
**PFLB**	**pflB**	b0903	2.3.1.54	pyruvate formate-lyase
PPB	phoA		3.1.3.1	alkaline phosphatase
**PROB**	**proB**	b0242	2.7.2.11	glutamate 5-kinase
**PRPB**	**prpB**	b0331	4.1.3.30	2-methylisocitrate lyase
**PUR1**	**purF**	b2312	2.4.2.14	amidophosphoribosyltransferase (ATase)
**PURA**	**purA**	b4177	6.3.4.4	adenylosuccinate synthetase
**PUR8**	**purB**	b1131	4.3.2.2	adenylosuccinate lyase
**PUTA**	**putA**	b1014	1.5.5.2, 1.2.1.88	bifunctional protein PutA
**PXPA**	**pxpA**	b0713	3.5.2.9	5-oxoprolinase subunit A
**PYRB**	**pyrB**	b4245	2.1.3.2	aspartate carbamoyltransferase catalytic subunit

Gene	Curation	Code in KEGG	EC code	Protein
**PYRC**	**pyrC**	b1062	3.5.2.3	dihydroorotase
**PYRI**	**pyrI**	b4244	2.1.3.2	aspartate carbamoyltransferase regulatory chain
**RIR1**	**nrdA**	b2234	1.17.4.1	ribonucleoside-diphosphate reductase 1 subunit alpha
**RIR2**	**nrdB**	b2235	1.17.4.1	ribonucleoside-diphosphate reductase 1 subunit beta
**RIR4**	**nrdF**	b2676	1.17.4.1	ribonucleoside-diphosphate reductase 2 subunit beta
**SCPB**	**scpB**	b2919	4.1.1.-	methylmalonyl-CoA decarboxylase
**SDHA**	**sdhA**	b0723	1.3.5.1	succinate dehydrogenase flavoprotein subunit
**SDHB**	**sdhB**	b0724	1.3.5.1	succinate dehydrogenase iron-sulfur subunit
**SDHE**	**sdhE**	Not found		
**SERA**	**serA**	b2913	1.1.1.95, 1.1.1.399	phosphoglycerate dehydrogenase
**SPEB**	**speB**	b2937	3.5.3.11	agmatinase
**SUCC**	**sucC**	b0728	6.2.1.5	succinyl-CoA synthetase subunit beta
**SUCD**	**sucD**	b0729	6.2.1.5	succinyl-CoA synthetase subunit alpha
**SUFS**	**sufS**	b1680	2.8.1.7, 4.4.1.16	cysteine desulfurase
**SYM**	**metG**	b2114	6.1.1.10	methionine-tRNA ligase
T2E5	Not found			
**TALB**	**talB**	b0008	2.2.1.2	transaldolase B
TAUA	tauA		7.2.2.-	taurine ABC transporter substrate-binding protein
**TAUD**	**tauD**	b0368	1.14.11.17	taurine dioxygenase
THGA	lacA		2.3.1.18	galactoside O-acetyltransferase
**TKT1**	**tktA**	b2935	2.2.1.1	transketolase 1
**TNAA**	**tnaA**	b3708	4.1.99.1	tryptophanase
**TOLC**	**tolC**	b3035	-	outer membrane protein TolC
**TRXB**	**trxB**	b0888	1.8.1.9	thioredoxin reductase
**TYPH**	**deoA**	b4382	2.4.2.4	thymidine phosphorylase
**TYSY**	**thyA**	b2827	2.1.1.45	thymidylate synthase
**UDP**	**udp**	b3831	2.4.2.3	uridine phosphorylase
**USHA**	**ushA**	b0480	3.1.3.5	5'-nucleotidase/UDP-sugar diphosphatase
**YDIF**	**ydiF**	b1694	2.8.3.8	acetate-CoA transferase
**YFCF**	**yfcF**	b2301	2.5.1.18	glutathione S-transferase
**YQHD**	**yqhD**	b3011	1.1.1.2	NADPH-dependent aldehyde reductase

P. aeruginosa (ATCC® 27853™).

Gene	Curation	Code in KEGG	EC code	Protein
**ACCC**	**accC**	PA4848	6.4.1.2, 6.3.4.14	acetyl-CoA carboxylase biotin carboxylase subunit
**ACEA**	**aceA**	PA2634	4.1.3.1, 4.1.3.30	isocitrate lyase
**ADE**	Not found			
**AK**	**lysC**	PA0904	2.7.2.4	aspartokinase
**ALGC**	**algC**	PA5322	5.4.2.8, 5.4.2.2	phosphomannomutase/phosphoglucomutase
ALGD	algD		1.1.1.132	GDP-mannose 6-dehydrogenase
ALGE	algE		-	outer membrane porin AlgE
AMIC	amiC		-	aliphatic amidase expression-regulating protein AmiC
**AMIE**	**amiE**	PA3366	3.5.1.4	acylamide amidohydrolase / aliphatic amidase
AMIR	amiR		-	transcriptional antitermination factor AmiR
AMPC	Not found			
ARS	atsA		-	arylsulfatase AtsA
AZOR1	azoR1		1.7.1.17	FMN-dependent NADH-azoreductase AzoR1
**BAUA**	bauA		2.6.1.18	beta-alanine--pyruvate aminotransferase
CAS6	Not found			
COAX	Not found		2.7.1.33	pantothenate kinase
**DAPB**	**dapB**	PA4759	1.17.1.8	4-hydroxy-tetrahydrodipicolinate reductase
**DAPD**	**dapD**	PA3666	2.3.1.117	2,3,4,5-tetrahydropyridine-2,6-dicarboxylate N-succinyltransferase
**FOLD**	**folD**	PA1796	1.5.1.5, 3.5.4.9	bifunctional 5,10-methylene-tetrahydrofolate dehydrogenase/5,10-methylene-tetrahydrofolate cyclohydrolase

Gene	Curation	Code in KEGG	EC code	Protein
FPTA	fptA		-	Fe(3+)-pyochelin receptor FptA
FTSI	ftsI		3.4.16.4	peptidoglycan D,D-transpeptidase FtsI
**GSA**	**hemL**	PA3977	5.4.3.8	glutamate-1-semialdehyde aminotransferase
GSPI	gspI		-	type II secretion system minor pseudopilin GspI
GSPJ	xcpW			GspJ family T2SS minor pseudopilin variant XcpW
GSPK	gspK		-	type II secretion system minor pseudopilin GspK
**HEM2**	**hemB**	PA5243	4.2.1.24	delta-aminolevulinic acid dehydratase / porphobilinogen synthase
HUTF	Not found		3.5.3.13	formimidoylglutamate deiminase
**HYDA**	Not found			
KINB	Not found			
LPXC	lpxC		3.5.1.108	UDP-3-O-acyl-N-acetylglucosamine deacetylase
**MASZ**	**glcB**	PA0482	2.3.3.9	malate synthase G
**NIRS**	**nirS**	PA0519	1.7.2.1, 1.7.99.1	nitrite reductase
**NORB**	**norB**	PA0524	1.7.2.5	nitric oxide reductase subunit B
**NORC**	**norC**	PA0523	-	nitric oxide reductase subunit C
PCTC	pctC		-	methyl-accepting chemotaxis protein PctC
**PHEC**	**pheC**	PA3475	4.2.1.51, 4.2.1.91	cyclohexadienyl dehydratase
**PHHY**	**pobA**	PA0247	1.14.13.2	4-hydroxybenzoate 3-monooxygenase
**PHNW**	**phnW**	PA1310	2.6.1.37	2-aminoethylphosphonate-pyruvate transaminase
PILM	pilM		-	type IV pilus biogenesis protein PilM/type IV pilus assembly protein PilM
PORO	oprO		-	outer membrane porin OprO
PQSA	pqsA		6.2.1.32	anthranilate--CoA ligase
PQSB	pqsB		-	2-heptyl-4(1H)-quinolone synthase subunit PqsB
PQSC	pqsC		-	2-heptyl-4(1H)-quinolone synthase subunit PqsC
PQSD	pqsD		2.3.1.262	anthraniloyl-CoA anthraniloyltransferase
PVCB	pvcB		-	paerucumarin biosynthesis oxygenase PvcB
PXPA3	Not found		3.5.2.9	5-oxoprolinase subunit PxpA
ROCR	rocR		-	two-component system response regulator cyclic di-GMP-specific phosphodiesterase RocR
SAHH	ahcY		3.13.2.1	adenosylhomocysteinase
**SERC**	**serC**	PA3167	2.6.1.52	3-phosphoserine/phosphohydroxythreonine aminotransferase
SYS	serS		6.1.1.11	serine-tRNA ligase
TGPA	tgpA		2.3.2.13	protein-glutamine gamma-glutamyltransferase TgpA
**THRH**	**thrH**	PA1757	3.1.3.3, 2.7.1.39	phosphoserine phosphatase
TSE1	tse1			type VI secretion system effector peptidoglycanhydrolase Tse1
TSI1	tsi1			type IV secretion system immunity protein Tsi1
**UBIX**	**ubiX**	PA4019	2.5.1.129	flavin prenyltransferase UbiX
WBPE	Not found			
Y1727	Not found			
Y3435	Not found			
YFIR	yfiR			diguanylate cyclase inhibitor YfiR

Section S10. Manual curation of the PDB entries for the studied bacteria.

S. aureus.

Gene name	PDB entry	PubMed ID	Include/exclude (reason)
ACCA/ACCD	2F9I	16460018	include
CLPP	3ST9	21900233	include
CLPP	5W18	31588734	include
COAW	4M7X	27759386	include
COAW	4M7Y	27759386	include
COAW	6AWG	To be published	include
CRTM	2ZCS	18276850	include
DDL	2I87	17015835	include
FABI	3GR6	19768684	include
FEMX	6SNR	33740396	include
G3P1	3K9Q	20620151	include
HIS7	2AE8	To be published	include
HLA	3M3R	20400691	exclude (mutant)
HLA	6U49	32179646	include
ILVC	6VO2	32198779	include
ILVC	6C55	To be published	include
ILVC	6C5N	To be published	include
ISDA	3QZM	21893067	include
LUKDV	6U2S	32179646	include
MAZF	4OF1	To be published	exclude (toxin)
NANA	5KZD	27943302	include
NOSO	1MJT	12467576	include
PANC	2X3F	20419351	include
SARR	1HSJ	11381122	include
SPA	5CBN	26980593	exclude (unsuitable for drug design)
TPIS	3M9Y	22813930	include
TPIS	3UWV	22813930	include
TPIS	3UWW	22813930	include

E. coli.

Gene name	PDB entry	PubMed ID	Include/exclude (reason)
AAT	1AHG	7664122	exclude (active site mutant)
AAT	1AMQ	7896726	include
AAT	1QIS	10708649	exclude (mutant)
AAT	1QIT	10708649	exclude (mutant)
AAT	1IX6	12488449	exclude (mutant)
AAT	5VWR	28816437	include
AAT	1ASA	To be published	exclude (mutant)
AAT	1ASE	To be published	include
AAT	2D61	To be published	exclude (mutant)
ACRB	4U8V	25248080	exclude (inactive mutant)
ACRB	5ENT	26976576	include
ACRB	5JMN	27982032	include
ACRB	6ZO5	34188038	include
ALDA	2IMP	17173928	include
AMPP	1A16	9520390	include
AMPP	1M35	12777807	include
AMPP	1W2M	16229471	exclude (metal substitution)
AMPP	1W7V	16229471	exclude (metal substitution)
AMPP	1WL6	16229471	exclude (metal substitution)
AMPP	1WL9	16229471	include
AMPP	1WLR	16229471	include

Gene name	PDB entry	PubMed ID	Include/exclude (reason)
AMPP	2BHA	16229471	include
AMPP	2BHC	16229471	exclude (metal substitution)
AMPP	2BN7	16229471	exclude (metal substitution)
AMPP	2BWU	16411772	exclude (mutant)
AMPP	2BWX	16411772	exclude (mutant)
AMPP	2BWY	16411772	exclude (mutant)
AMTB	1U77	15361618	include
AMTB	1U7C	15361618	include
AMTB	1U7G	15361618	include
AMTB	2NMR	17040913	exclude (mutant)
AMTB	2NOP	17040913	exclude (mutant)
AMTB	2NOW	17040913	exclude (mutant)
AMTB	2NPD	17040913	exclude (mutant)
AMTB	2NPE	17040913	exclude (mutant)
AMTB	2NS1	17190799	exclude (complex with other protein)
AMTB	3C1G	18362341	exclude (mutant)
AMTB	3C1H	18362341	exclude (mutant)
AMTB	3C1I	18362341	exclude (mutant)
AMTB	3C1J	18362341	exclude (mutant)
ARAA	4F2D	To be published	include
ARGB	1OHB	12875848	exclude (mutant affecting conformation)
ASPG1	2P2N	17451745	include
ASSY	1KP3	11809762	include
ATDA	6CY6	31205017	include
BFR	2VXI	18946693	include
BGAL	1DP0	11045615	include
BGAL	1JYN	11732897	include
BGAL	1JYV	11732897	include
BGAL	1JZ2	11732897	include
BGAL	1JZ3	11732897	include
BGAL	1JZ4	11732897	include
BGAL	1JZ6	11732897	include
BGAL	1JZ7	11732897	include
BGAL	1PX4	14621996	exclude (mutant)
BGAL	3DYO	19472413	exclude (mutant)
BGAL	3DYP	19472413	exclude (mutant)
BGAL	3I3B	20921997	exclude (mutant)
BGAL	3I3D	20921997	exclude (mutant)
BGAL	3I3E	20921997	exclude (mutant)
BGAL	3MUY	21102659	exclude (mutant)
BGAL	3MV0	21102659	exclude (mutant)
BGAL	3SEP	22155115	exclude (mutant)
BGAL	3T0A	22155115	exclude (mutant)
BGAL	3T0B	22155115	exclude (mutant)
BGAL	3VDA	22446164	exclude (mutant)
BGAL	4DUX	23486479	exclude (mutant)
BGAL	4TTG	25820412	include
BGAL	6TSH	31877353	include
BGAL	6TTE	31877353	include
BGAL	6X1Q	32695410	include
BGAL	4DUV	To be published	exclude (mutant)
BGLR	3K46	21051639	include
BGLR	3LPF	21051639	include
BGLR	5CZK	26364932	include

Gene name	PDB entry	PubMed ID	Include/exclude (reason)
BGLR	6LEL	33664385	include
CADC	5JU7	28432336	include
CARA/CARB	1A9X	9636022	exclude (mutant)
CARA	1JDB	10089390	include
CARA/CARB	1BXR	10029528	include
CARA/CARB	1CE8	10428826	include
CARA/CARB	1C30	10587438	exclude (mutant)
CARA/CARB	1C3O	10587438	exclude (mutant)
CARA/CARB	1CS0	10587438	exclude (mutant)
CARA/CARB	1KEE	11729189	include
CARA/CARB	1M6V	12130656	exclude (mutant)
CARA/CARB	1T36	15322282	exclude (mutant)
CATE	1GGF	11455600	exclude (mutant)
CATE	1GGK	11455600	exclude (mutant)
CATE	1P7Y	12777389	exclude (mutant)
CATE	1P80	12777389	exclude (mutant)
CATE	1P81	12777389	exclude (mutant)
CATE	1QWS	12777389	exclude (mutant)
CATE	3P9P	21332158	exclude (mutant)
CATE	3P9R	21332158	exclude (mutant)
CATE	3P9S	21332158	exclude (mutant)
CATE	3PQ2	21332158	exclude (mutant)
CATE	3PQ3	21332158	exclude (mutant)
CATE	3PQ5	21332158	exclude (mutant)
CATE	3PQ8	21332158	exclude (mutant)
CATE	3TTT	22172685	exclude (mutant)
CATE	6ZTW	33485289	include
CLPP	1TYF	9390554	include
CLPP	1YG6	16406682	include
CLPP	3MT6	20851345	include
COAD	6B7E	29190085	exclude (docking protocol)
COAD	6CCO	29498517	include
COAD	6CCQ	29498517	include
COAD	6CCS	29498517	include
COAD	6CHM	29551072	include
COAD	6CHQ	29551072	include
COAD	6CKW	29551072	include
CODA	1K6W	11812140	include
CODA	1K70	11812140	include
CODA	1R9Y	15381761	exclude (mutant)
CODA	1R9Z	15381761	exclude (mutant)
CODA	1RA5	15381761	exclude (mutant)
CODA	3G77	19487291	exclude (mutant)
CODA	3O7U	21545144	include
CPOB	2WZ7	20816983	include
CRP	1HW5	11124966	exclude (mutant)
CRP	4I09	24058293	exclude (mutant)
DHAS	1BRM	10369777	include
DHAS	1T4B	15288787	include
FABI	1D8A	10201369	include
FABI	1QSG	10398587	include
FABI	1I30	11527706	include
FABI	3PJF	21094257	exclude (mutant)
FUCI	1FUI	9367760	include

Gene name	PDB entry	PubMed ID	Include/exclude (reason)
FUMC	1YFE	16204892	include
GLPG	6PJQ	31570873	include
GLPG	6VJ8	32888502	exclude (mutant)
GLPG	6VJ9	32888502	exclude (mutant)
GLPG	2IC8	17051161	include
GLPG	5MTF	29107700	include
GLPG	5MT8	29107700	include
IDH	1IDC	7761851	exclude (mutant)
IDH	1IDE	7761851	exclude (mutant)
IDH	1IDF	7761851	exclude (mutant)
IDH	4AJ3	22891681	include
IDH	1AI2	9211842	exclude (cation substitution, Ca with Mg)
HEM2	1B4E	10194344	include
HIS8	1GEW	11294630	include
HIS8	1GEY	11294630	include
HIS8	1FG3	11518529	include
HIS8	1FG7	11518529	include
HPPK	1EX8	11311059	include
HPPK	3HSG	To be published	exclude (mutant)
KDSC	2R8E	19726684	include
LDCI	3N75	21278708	include
MALE	6KI0	33431827	exclude (E.coli used as expression system)
MALE	6QGD	31459977	exclude (E.coli used as expression system)
MALE	1HSJ	12794084	exclude (protein fusion)
MALE	5AZ7	26694222	exclude (protein fusion)
MALE	7CY5	25909780	exclude (E.coli used as expression system)
MALE	7CY4	28202898	exclude (E.coli used as expression system)
MALE	3MP8	21685874	exclude (E.coli used as expression system)
MALE	4WMT	25909780	exclude (protein fusion)
MALE	4WMX	25909780	exclude (protein fusion)
MALE	7CY7	33531488	exclude (synthetic construct)
MALE	4RWF	25982113	include
MCBA	6GRG	30661981	include
MCBA	6GRG	30661981	include
NARG/NARH/NARI	1SIW	15122898	exclude (low temperature, <15K)
NARG/NARH/NARI	1Y5L	15615728	include
NARG/NARH/NARI	1Y5N	15615728	include
NARG/NARH/NARI	3IR7	20053990	include
NARG/NARH/NARI	3EGW	To be published	include
ODO2	1SCZ	To be published	include
ODP1	1RP7	14992577	include
ODP1	2QTC	17635929	include
ODP1	3LPL	20106967	include
PCKA	1K3D	11724534	include
PCKA	2OLQ	17475535	include
PCKA	2OLR	17475535	include
PCKA	2PY7	To be published	include
PCKA	6CRT	To be published	include
PDXJ	1M5W	12269807	include
PFKA	1PFK	2975709	include
PPB	1ANJ	7473737	exclude (mutant)
PPB	1URB	8648634	exclude (mutant)
PPB	1KH7	11884134	exclude (mutant)
PUR1	1ECG	8663035	exclude (inactivated enzyme)

Gene name	PDB entry	PubMed ID	Include/exclude (reason)
PUR1	1ECF	9514258	include
PYRC	2EG6	17550785	include
PYRC	2Z25	17711307	exclude (mutant)
RIR2	1MRR	1328209	exclude (cation substitution, Fe with Mn)
RIR2	1BIQ	9692970	exclude (mutant)
RIR2	1JQC	11315567	exclude (cation substitution)
RIR2	1PIZ	14677973	exclude (mutant)
RIR2	1R65	14677973	include
RIR2	2X0X	20518462	exclude (mutant)
RIR2	1PIM	10.1021/ja991839l	exclude (mutant)
RIR2	1PIU	10.1021/ja991839l	exclude (mutant)
TAUA	6SSY	31802112	include
THGA	1KRR	11937062	include
TKT1	2R8P	17914867	include
TKT1	6TJ9	32839604	include
UDP	1LX7	12499542	include
UDP	1RXC	15003451	include
UDP	1RXY	15003451	include
UDP	1T0U	15003451	include
UDP	1U1C	15983408	include
UDP	1U1D	15983408	include
UDP	1U1E	15983408	include
UDP	1U1F	15983408	include
UDP	1U1G	15983408	include
UDP	3KVV	20364833	include
UDP	1TGV	To be published	include
UDP	1TGY	To be published	include

P. aeruginosa.

Gene name	PDB entry	PubMed ID	Include/exclude (reason)
ACCC	2C00	18725455	include
AK	5YEI	29382741	include
ALGD	1MV8	12705829	include
ALGE	5D5D	26894538	exclude (unsuitable for drug design)
AMIC/AMIR	1QO0	10508151	include
ARS	4CXU	30012610	exclude (laboratory evolution)
AZOR1	3KEG	20057057	exclude (mutant)
BAUA	4B98	23519665	include
BAUA	4B9B	23519665	include
COAX	2F9W	16905099	include
FPTA	1XKW	16139844	include
FTSI	3PBT	21135211	include
FTSI	7ATO	34337941	include
GSPI/GSPJ/GSPK	5VTM	30346996	include
HEM2	1W5M	15644204	exclude (mutant)
HEM2	2C14	16819823	include
HEM2	2C15	16819823	include
HUTF	4RDV	To be published	include
LPXC	3UHM	22257165	include
LPXC	7K99	33160146	include
LPXC	7CI5	33210531	include
NORB/NORC	3WFC	24338896	include
NORB/NORC	3WFD	24338896	include
NORB/NORC	3WFE	24338896	include
PCTC	5LTV	31964737	include

Gene name	PDB entry	PubMed ID	Include/exclude (reason)
PHHY	1DOC	7939628	include
PHHY	1PXA	8312276	exclude (mutant)
PHHY	1PXB	8312276	exclude (mutant)
PHHY	1PXC	8312276	exclude (mutant)
PHHY	1IUT	8555229	include
PHHY	1IUV	8555229	include
PHHY	1IUW	8555229	include
PHHY	6JU1	31639299	exclude (mutant, enzyme design)
PILM	5EOX	27022027	include
PORO	4RJX	26445443	include
PQSB/PQSC	5DWZ	26811339	include
PQSD	3H76	19694421	include
PQSD	3H77	19694421	include
PVCB	3EAT	18824174	include
PXPA3	2XU2	20419351	include
ROCR	3SY8	22753070	include
SAHH	6F3P	30054521	include
SYS	6HDZ	31869198	include
SYS	6HE1	31869198	include
TGPA	6G49	30599211	include
TSE1/TSI1	3VPJ	22700987	exclude (complex with immune protein)
UBIX	4ZAF	26083743	include
UBIX	4ZAG	26083743	include
UBIX	4ZAV	26083743	include
UBIX	4ZAW	26083743	include
UBIX	4ZAY	26083743	include
UBIX	4ZAZ	26083743	include
YFIR	5EB3	27113583	include

Section S11. Alluvial plots

S. aureus

Beta-Alanine metabolism (https://www.kegg.jp/pathway/sac00410)

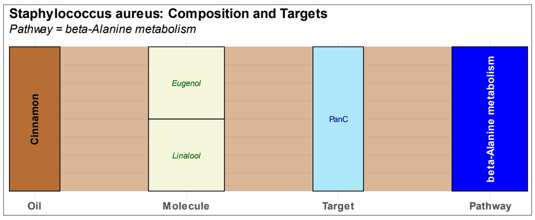

Pantothenate and CoA biosynthesis (https://www.kegg.jp/pathway/sac00770)

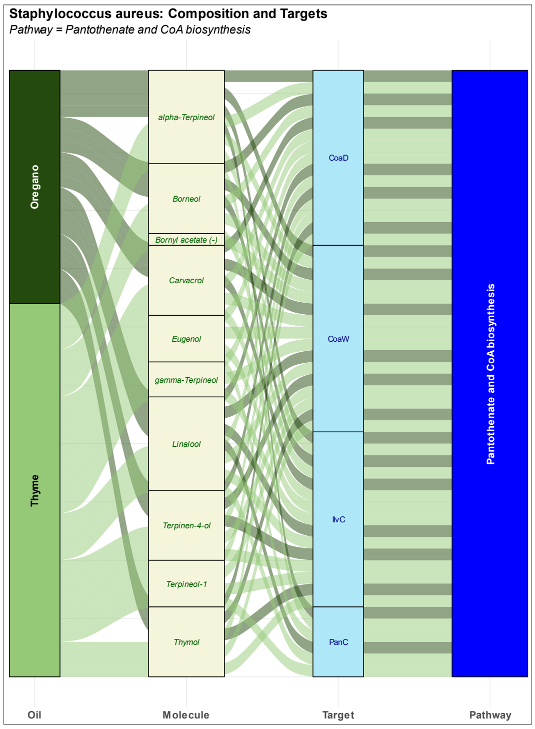

E. coli

2-Oxocarboxylic acid metabolism (https://www.kegg.jp/pathway/eco01210)

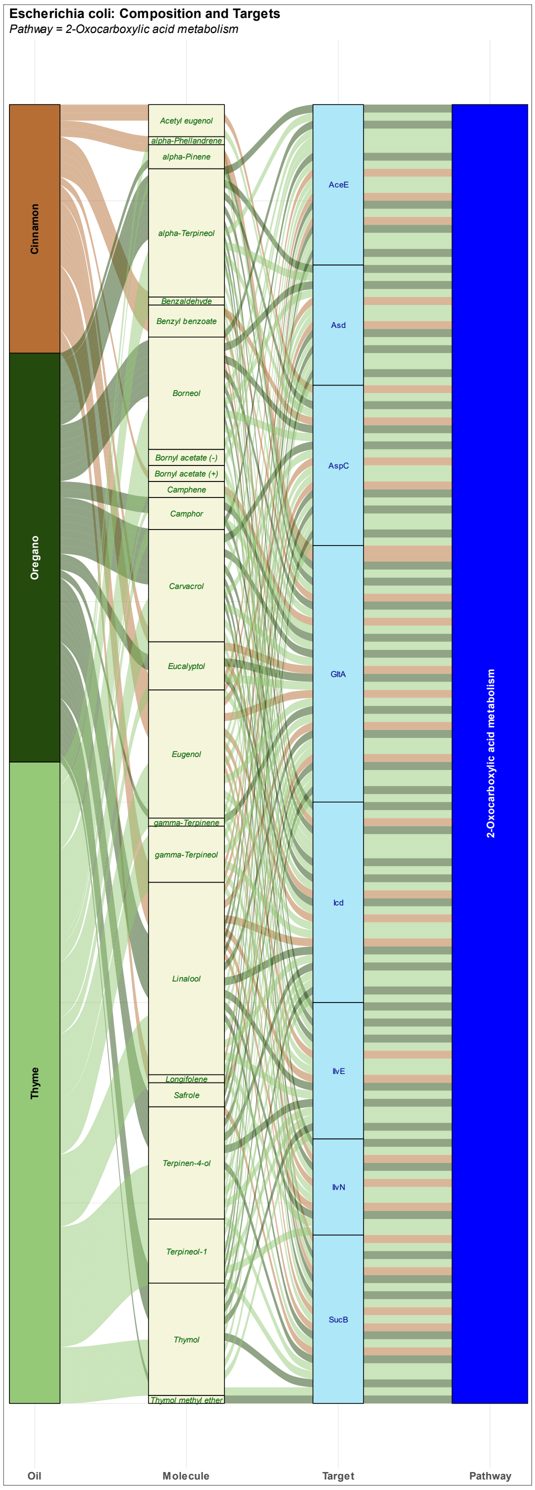

Alanine, aspartate and glutamate metabolism (https://www.kegg.jp/pathway/eco00250)

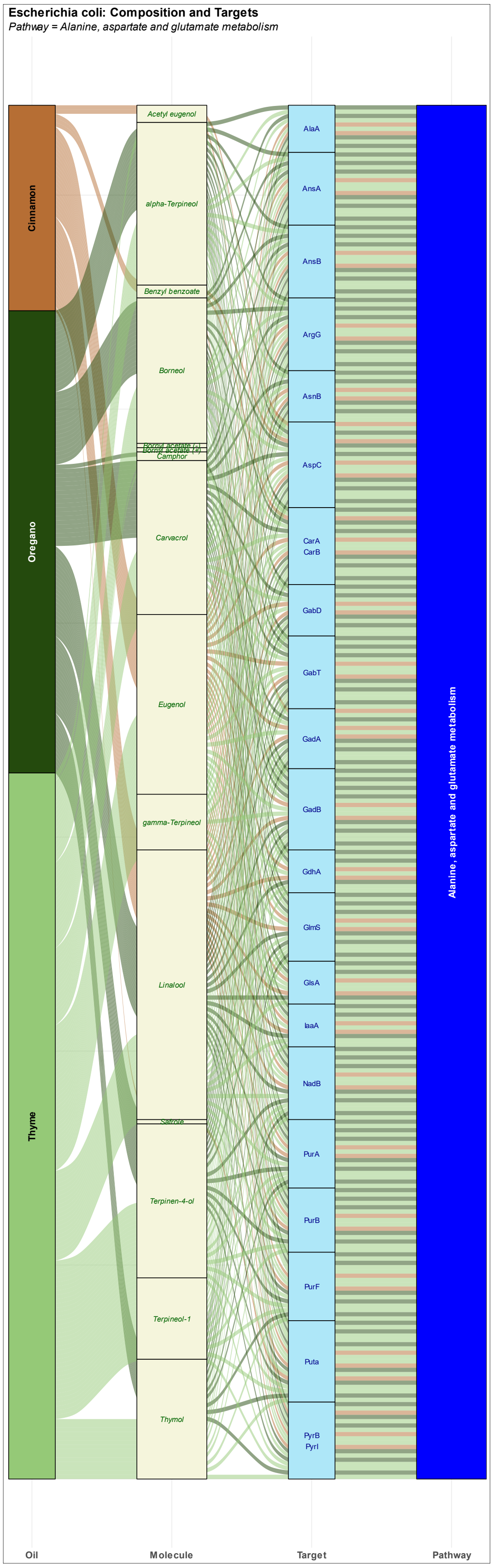

Arginine and proline metabolism (https://www.kegg.jp/pathway/eco00330)

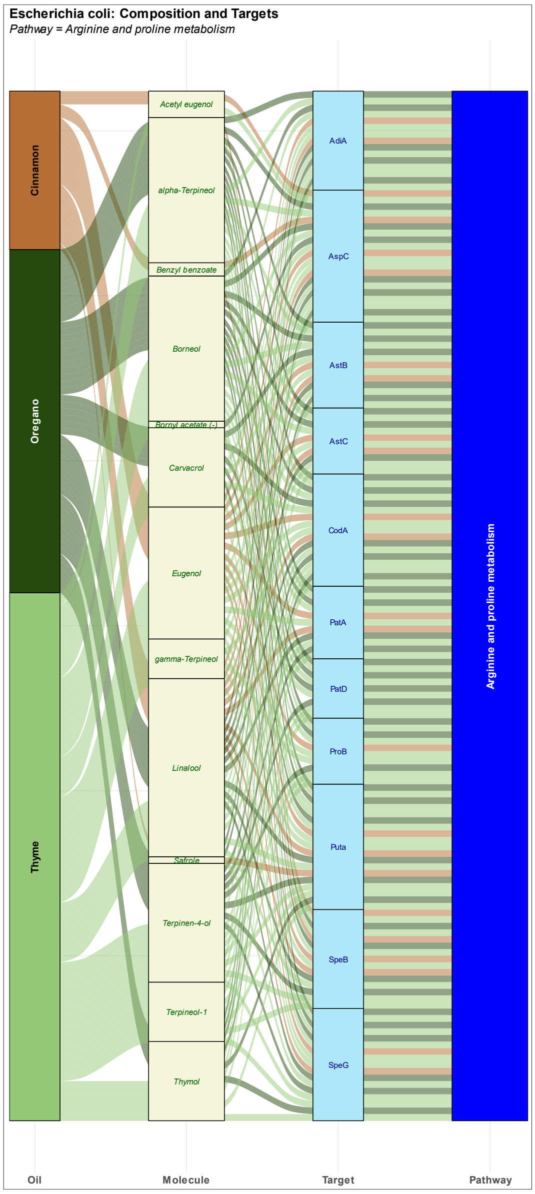

Bacterial chemotaxis (https://www.kegg.jp/pathway/eco02030)

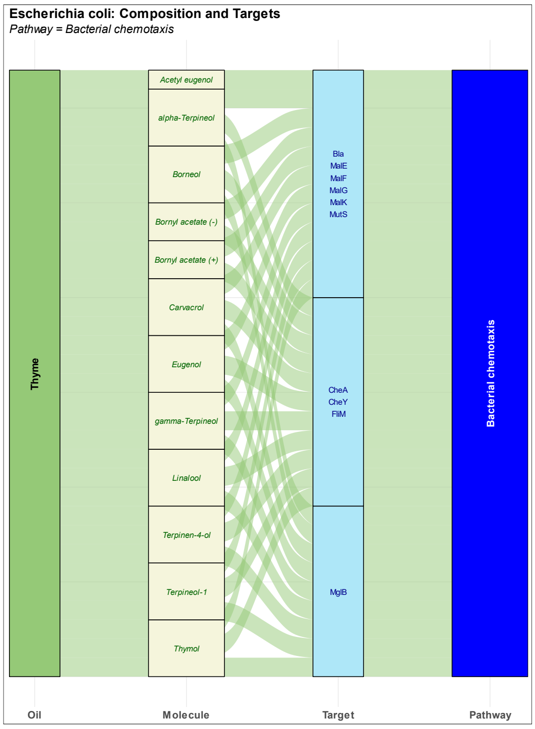

beta-Alanine metabolism (https://www.kegg.jp/pathway/eco00410)

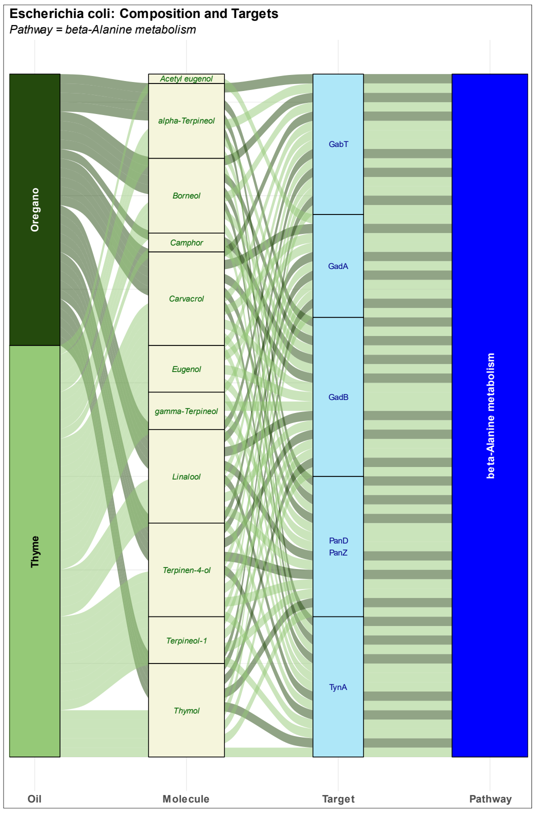

beta-Lactam resistance (https://www.kegg.jp/pathway/eco01501)

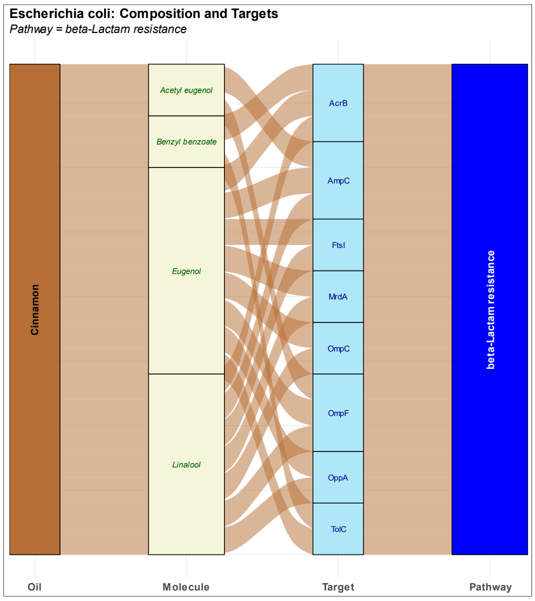

Butanoate metabolism (https://www.kegg.jp/pathway/eco00650)

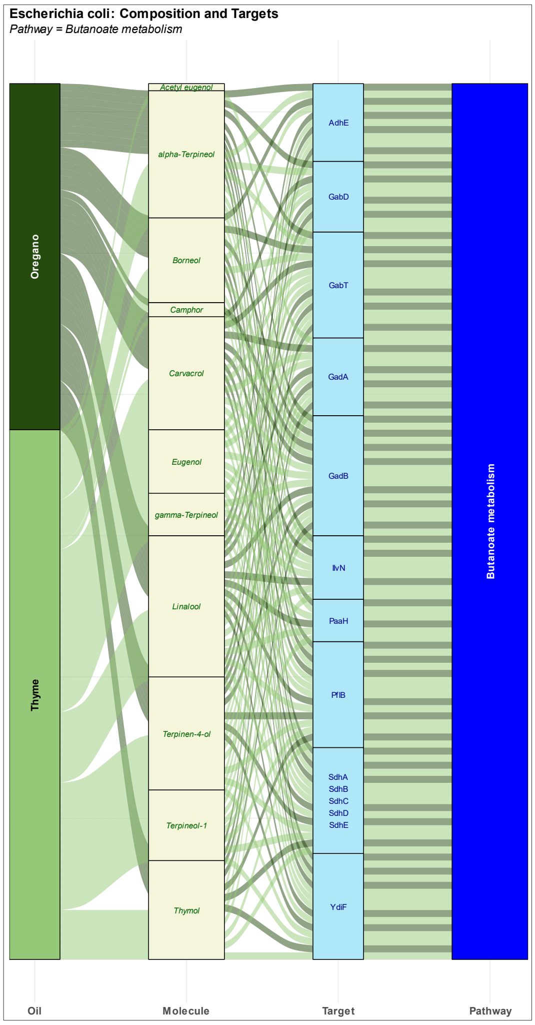

Citrate cycle (TCA cycle) (https://www.kegg.jp/pathway/eco00020)

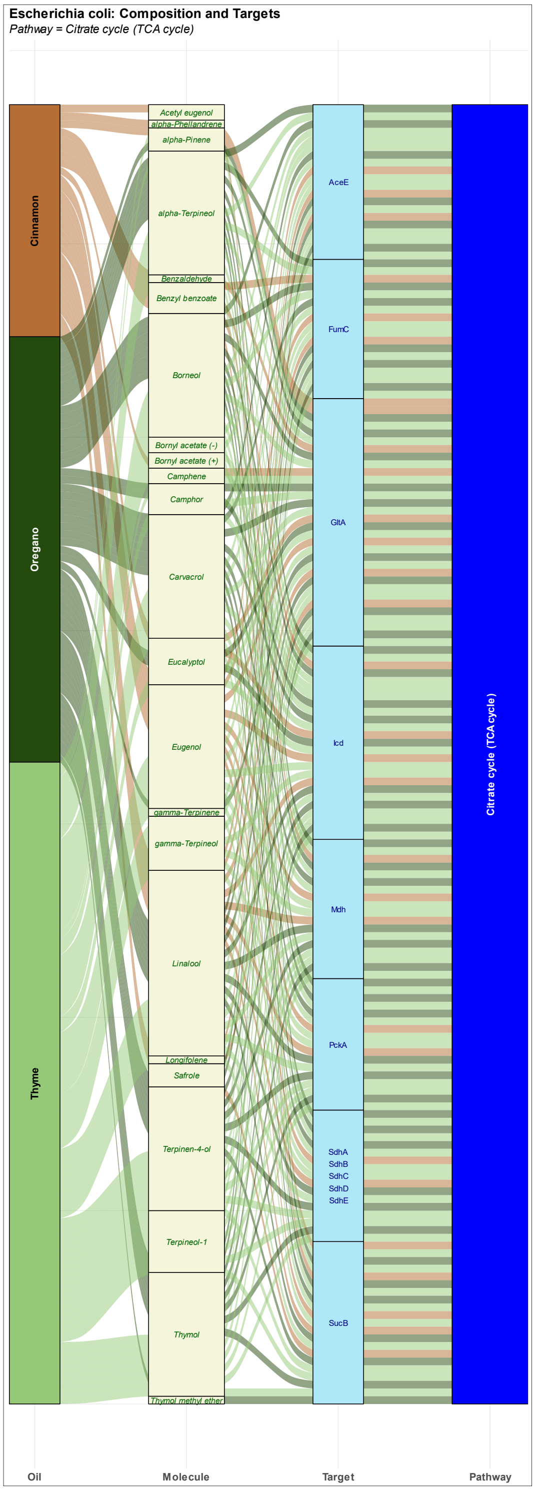

Cyanoamino acid metabolism (https://www.kegg.jp/pathway/eco00460)

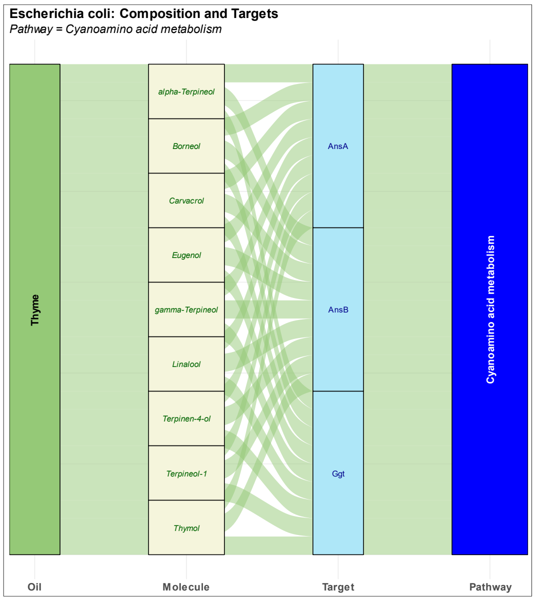

Fatty acid biosynthesis (https://www.kegg.jp/pathway/eco00061)

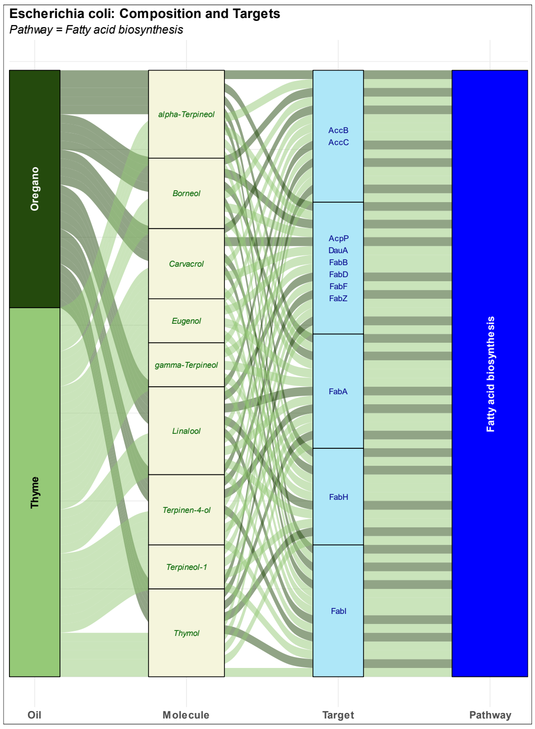

Fatty acid metabolism (https://www.kegg.jp/pathway/eco01212)

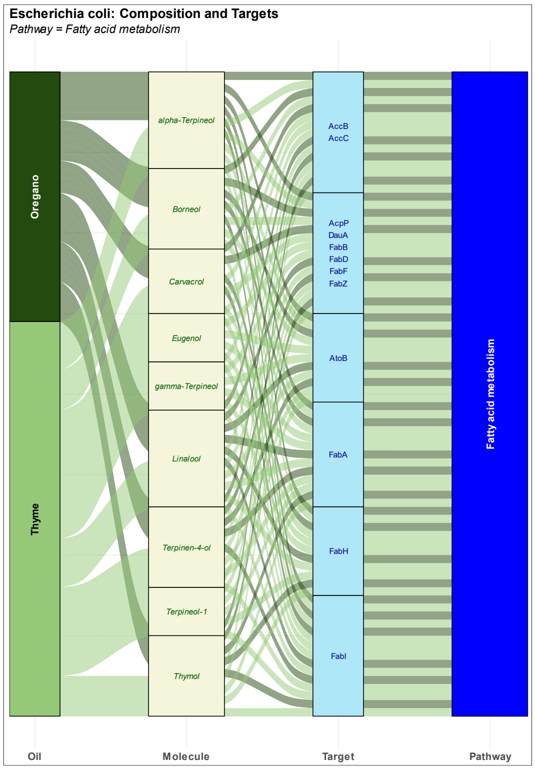

Glutathione metabolism (https://www.kegg.jp/pathway/eco00480)

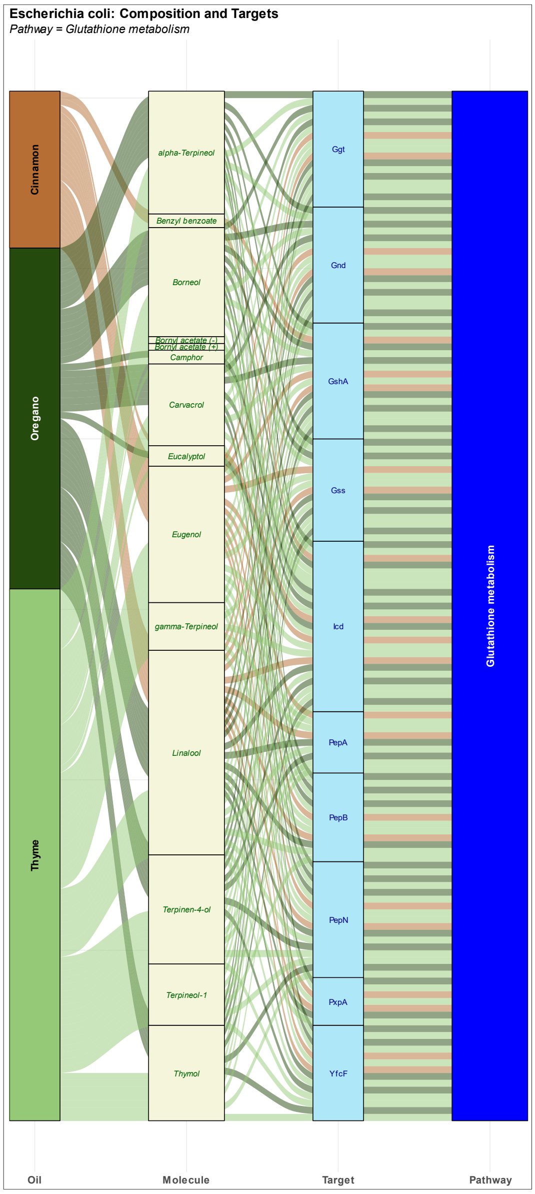

Glyoxylate and dicarboxylate metabolism (https://www.kegg.jp/pathway/eco00630)

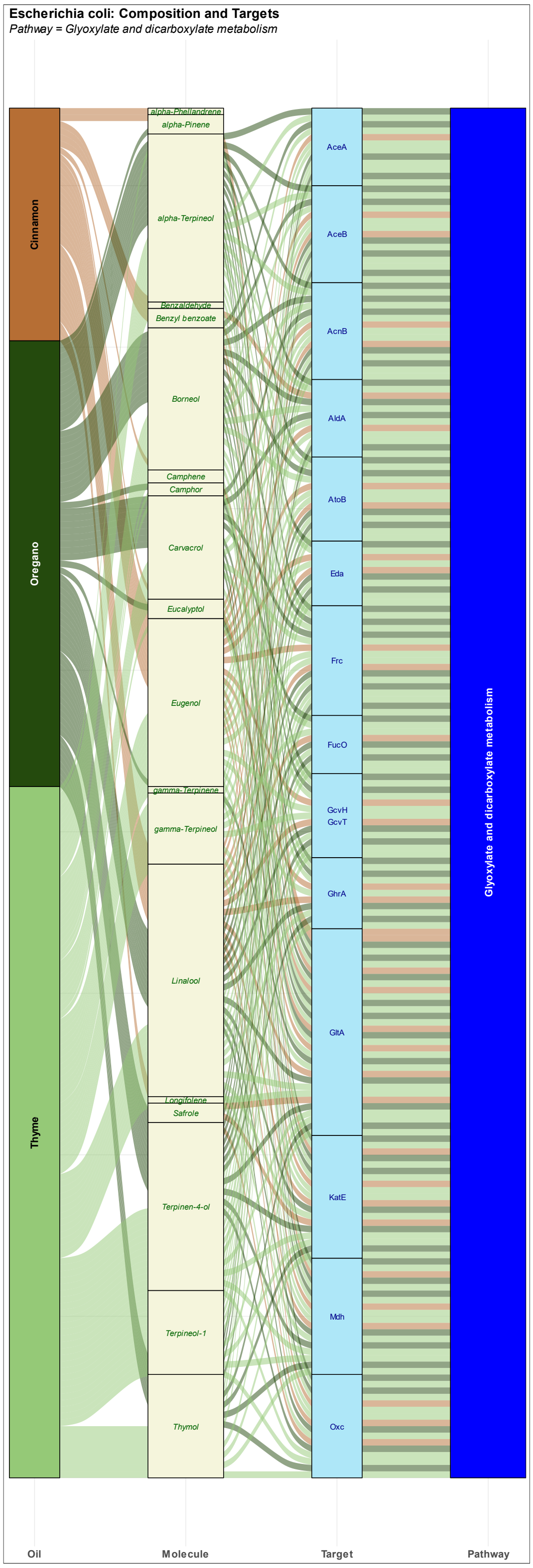

Lipoic acid metabolism (https://www.kegg.jp/pathway/eco00785)

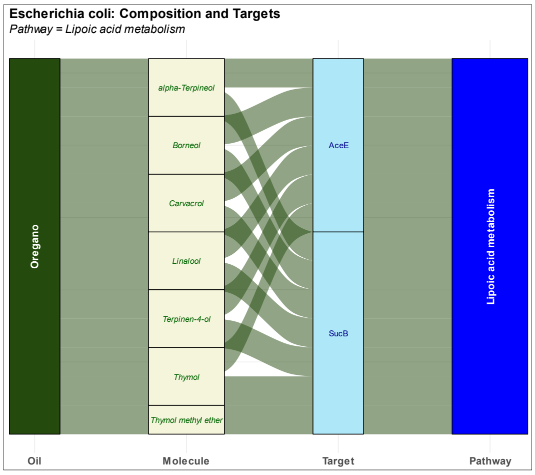

Methane metabolism (https://www.kegg.jp/pathway/eco00680)

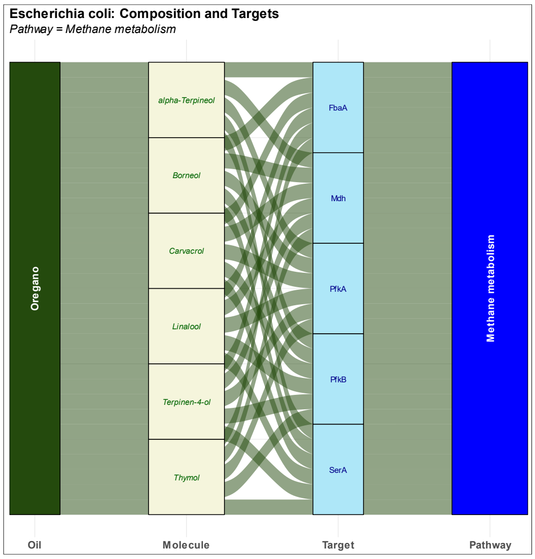

Monobactam biosynthesis (https://www.kegg.jp/pathway/eco00261)

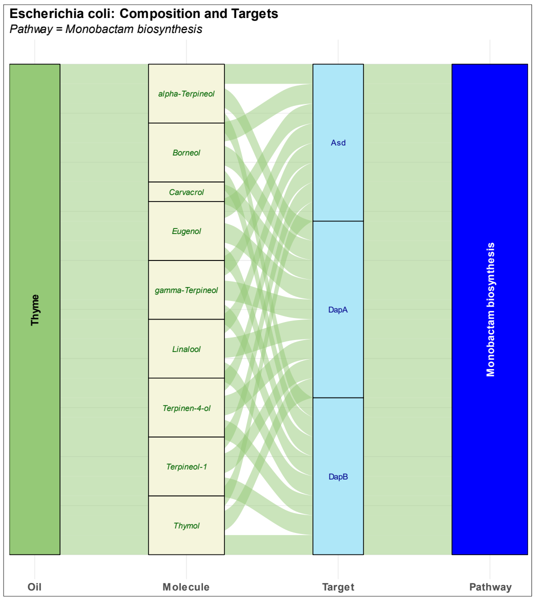

Nitrogen metabolism (https://www.kegg.jp/pathway/eco00910)

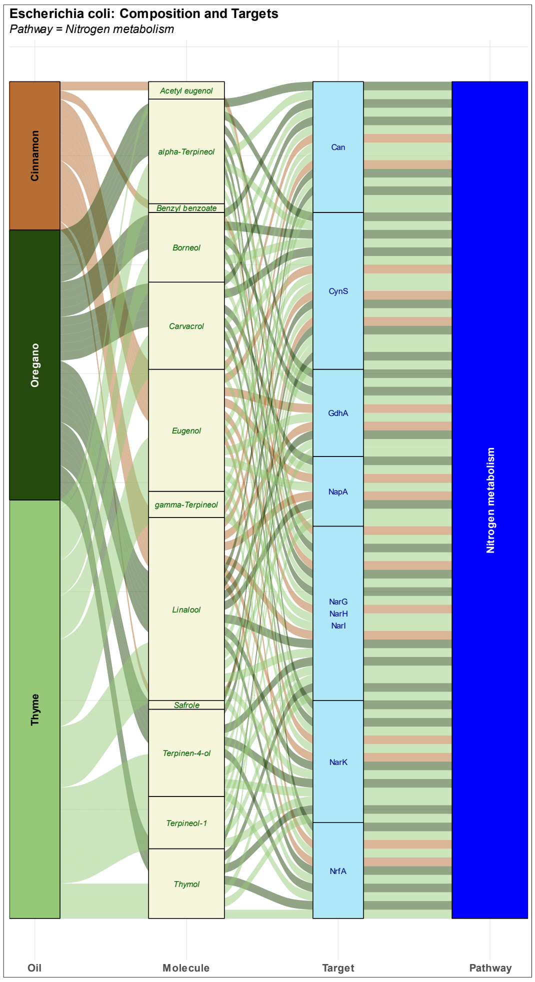

Nitrotoluene degradation (https://www.kegg.jp/pathway/eco00633)

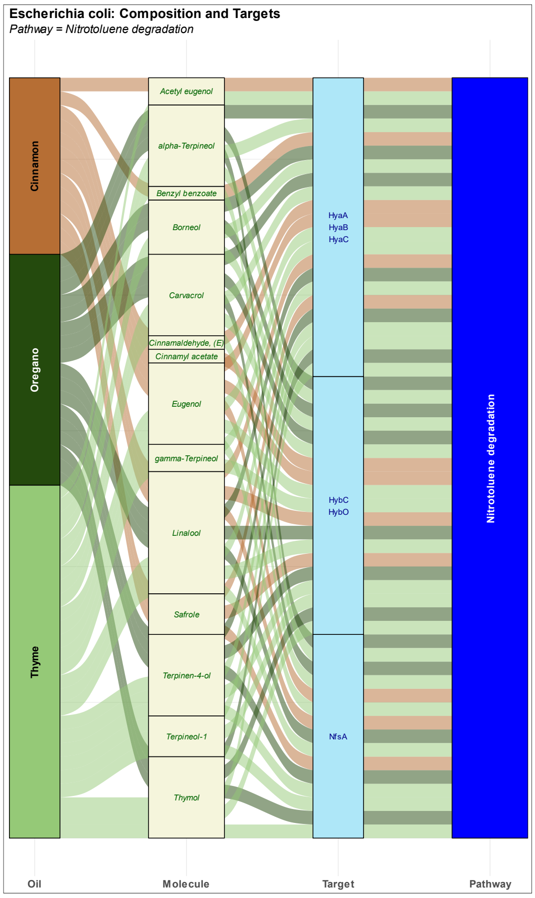

Novobiocin biosynthesis (https://www.kegg.jp/pathway/eco00401)

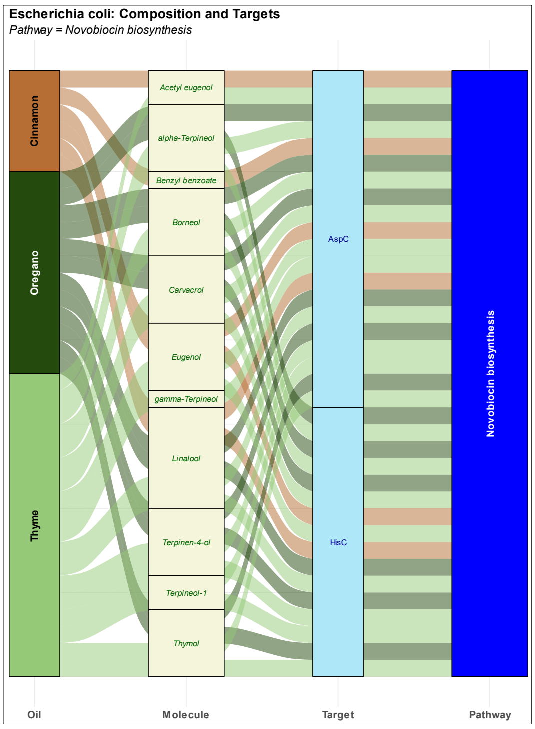

Other carbon fixation pathways (https://www.kegg.jp/pathway/eco00720)

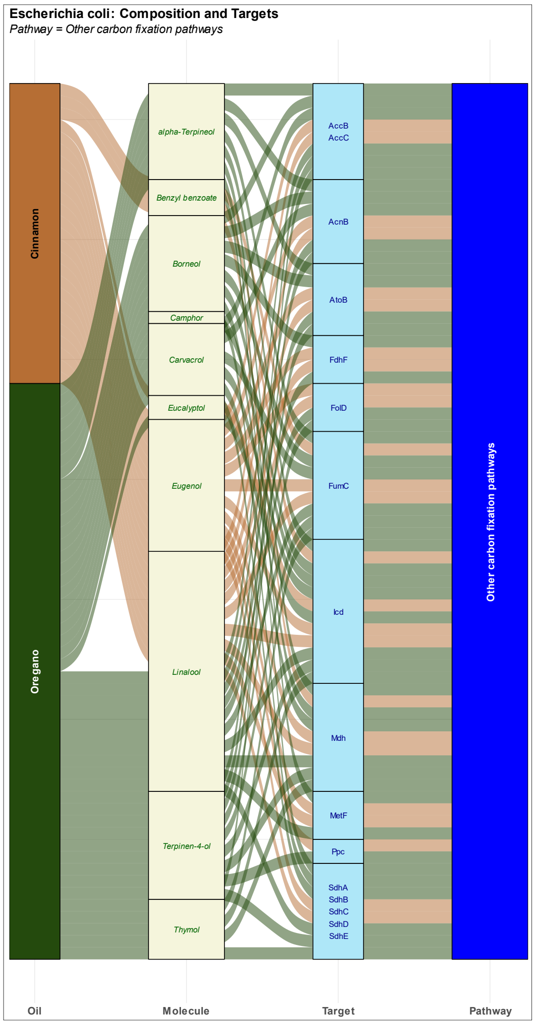

Other glycan degradation (https://www.kegg.jp/pathway/eco00511)

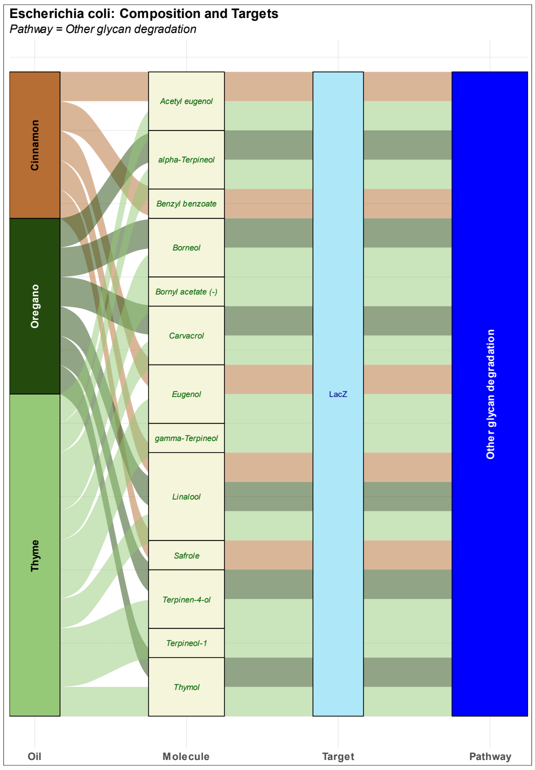

Pantothenate and CoA biosynthesis (https://www.kegg.jp/pathway/eco00770)

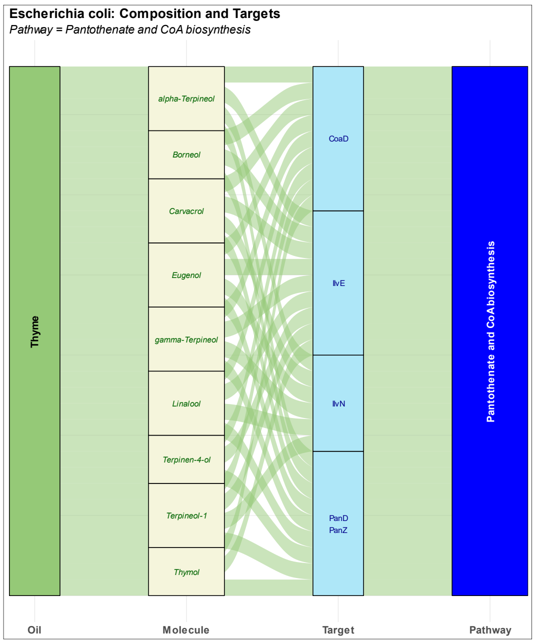

Pentose phosphate pathway (https://www.kegg.jp/pathway/eco00030)

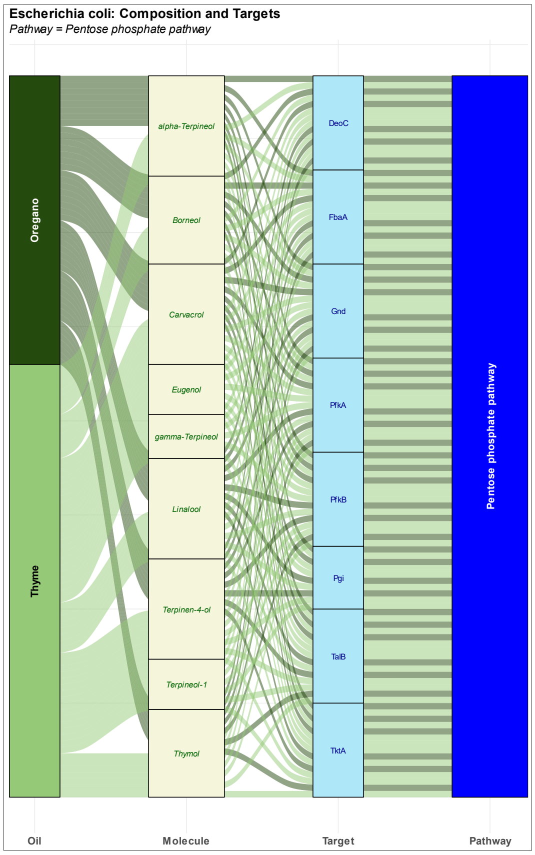

Porphyrin metabolism (https://www.kegg.jp/pathway/eco00860)

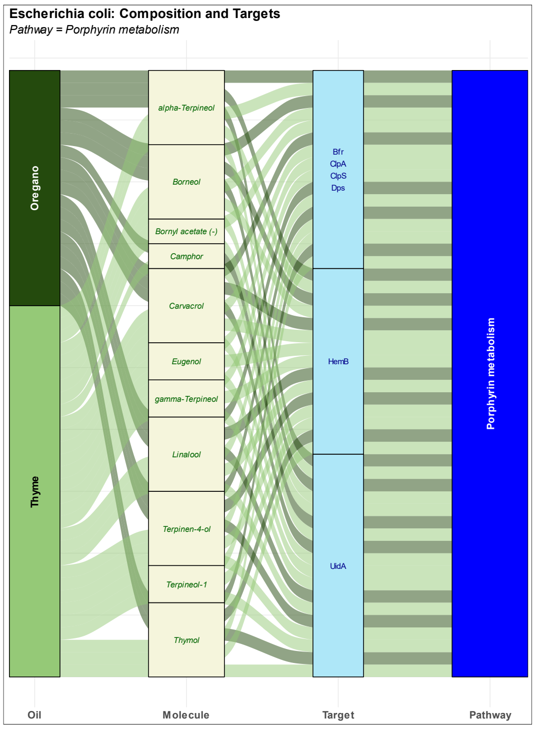

Propanoate metabolism (https://www.kegg.jp/pathway/eco00640)

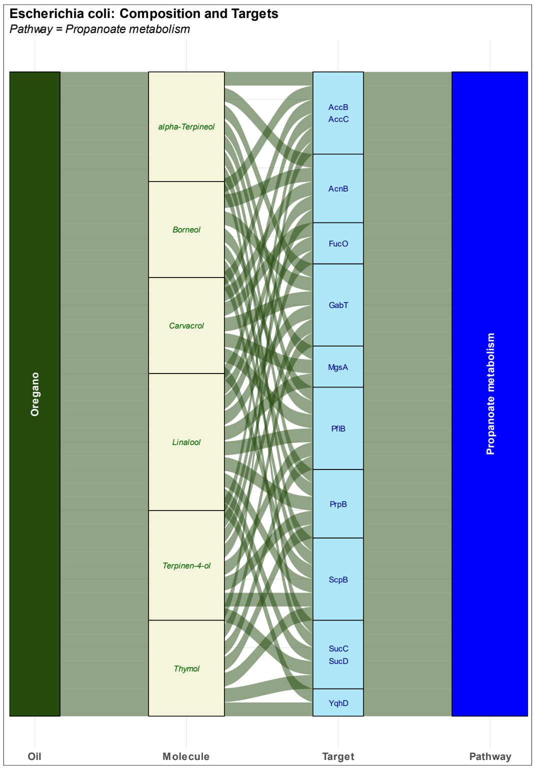

Pyrimidine metabolism (https://www.kegg.jp/pathway/eco00240)

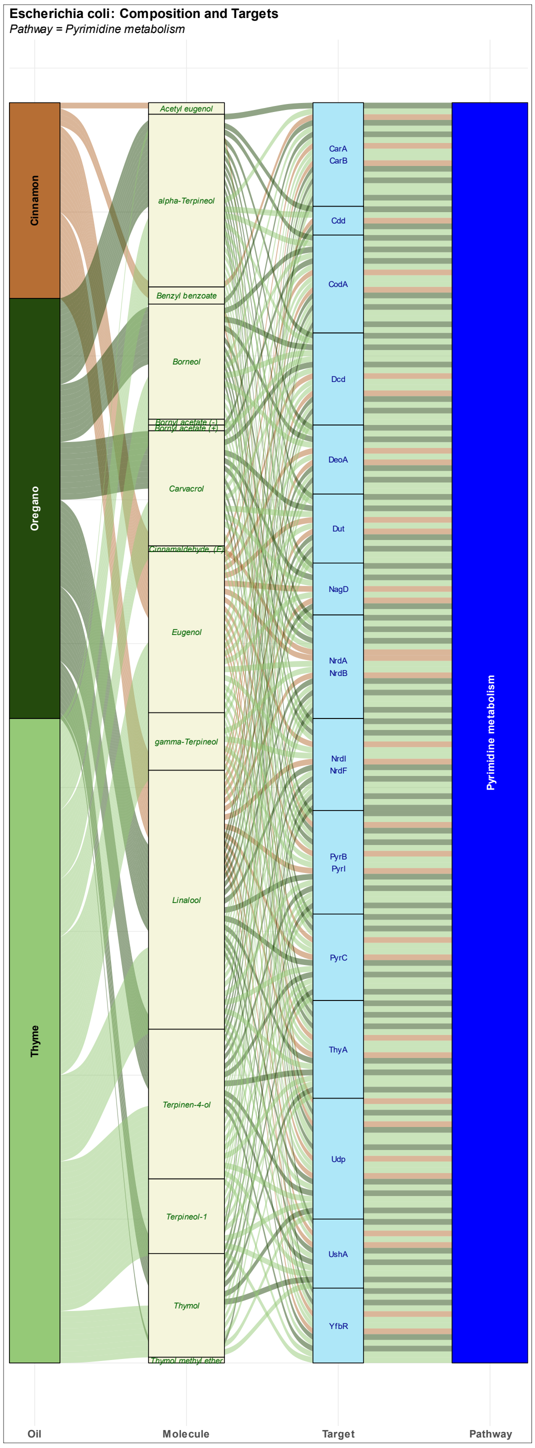

Selenocompound metabolism (https://www.kegg.jp/pathway/eco00450)

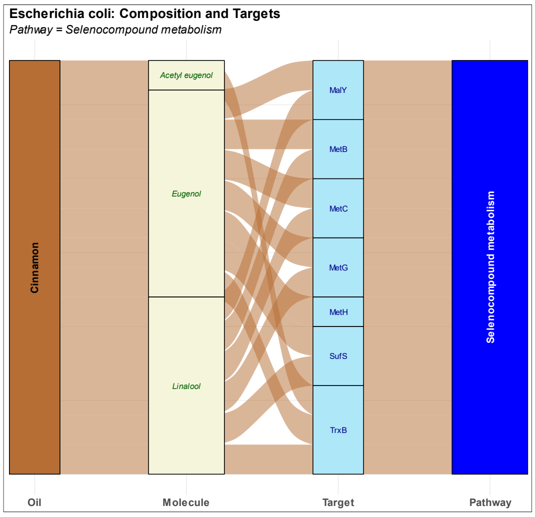

Sphingolipid metabolism (https://www.kegg.jp/pathway/eco00600)

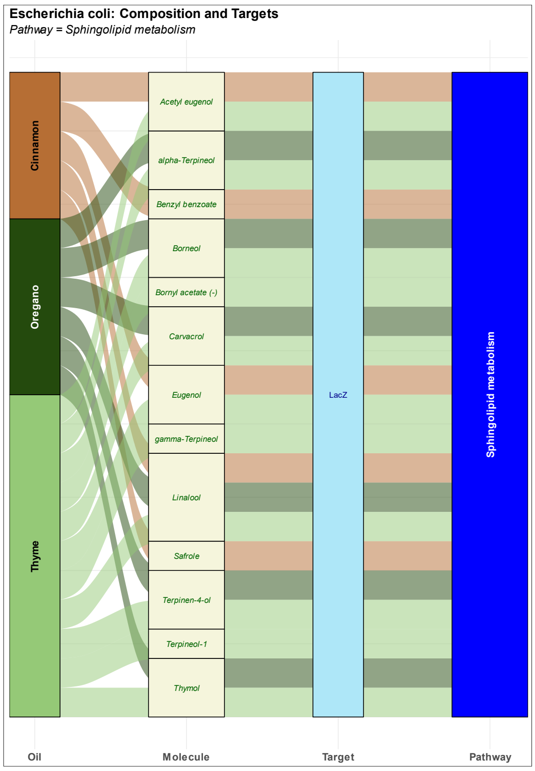

Taurine and hypotaurine metabolism (https://www.kegg.jp/pathway/eco00430)

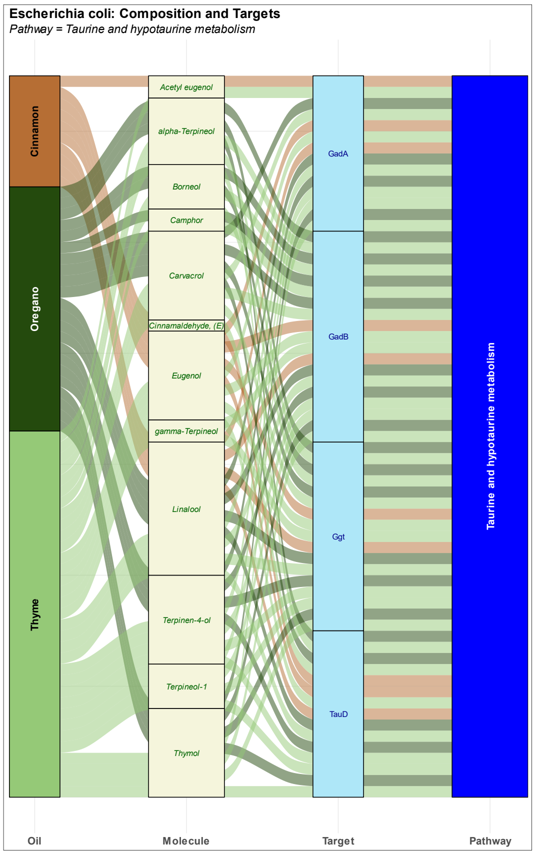

Tryptophan metabolism (https://www.kegg.jp/pathway/eco00380)

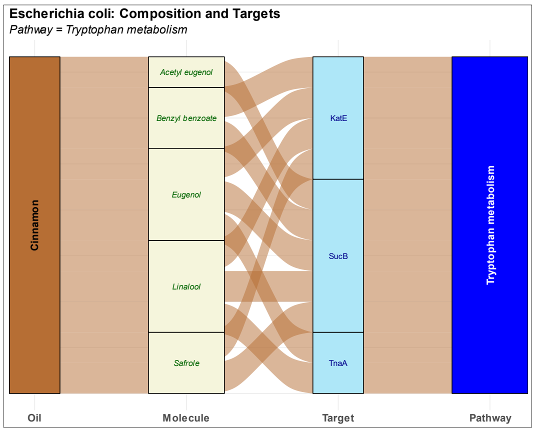

P. aeruginosa

Benzoate degradation (https://www.kegg.jp/pathway/pae00362)

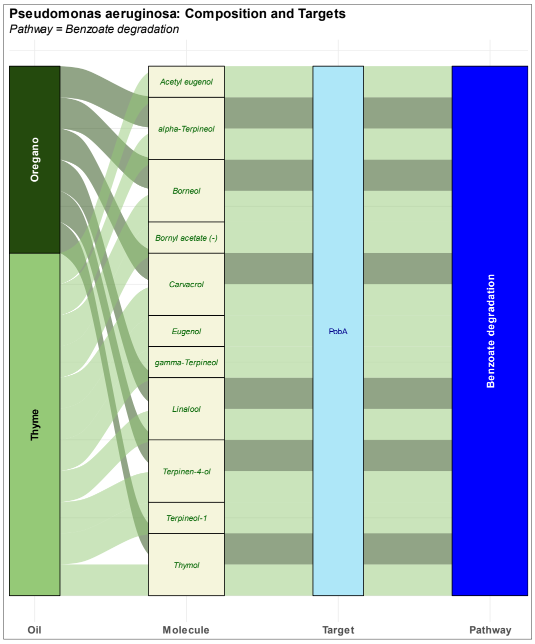

Degradation of aromatic compounds (https://www.kegg.jp/pathway/pae01220)

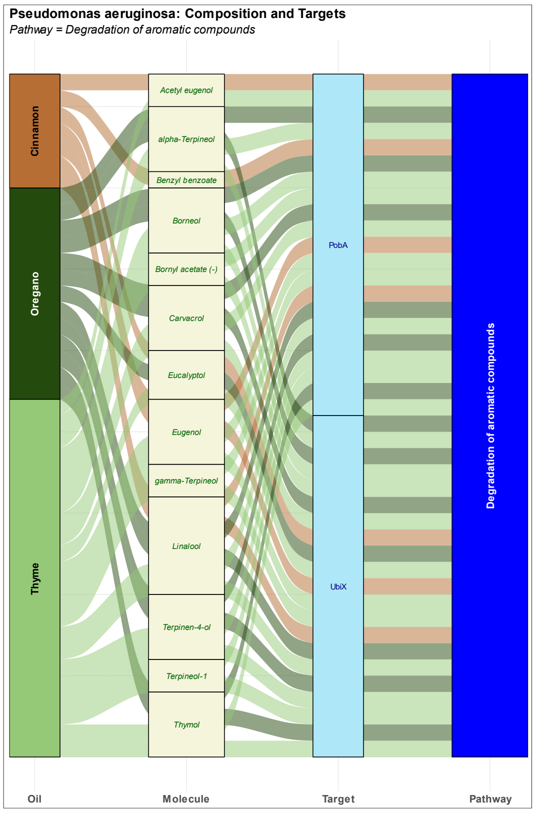

Fatty acid biosynthesis (https://www.kegg.jp/pathway/pae00061)

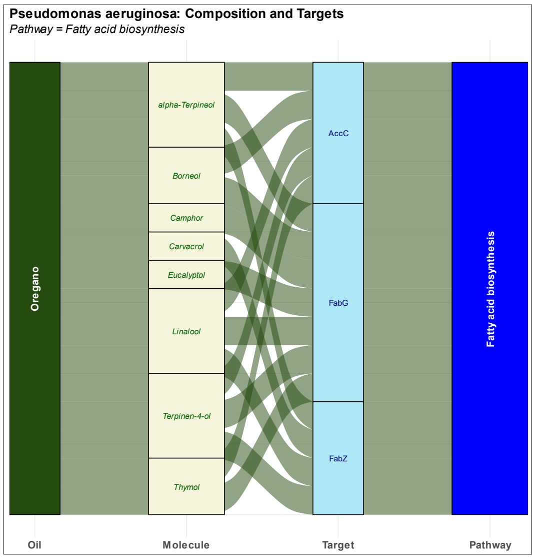

Fatty acid metabolism (https://www.kegg.jp/pathway/pae01212)

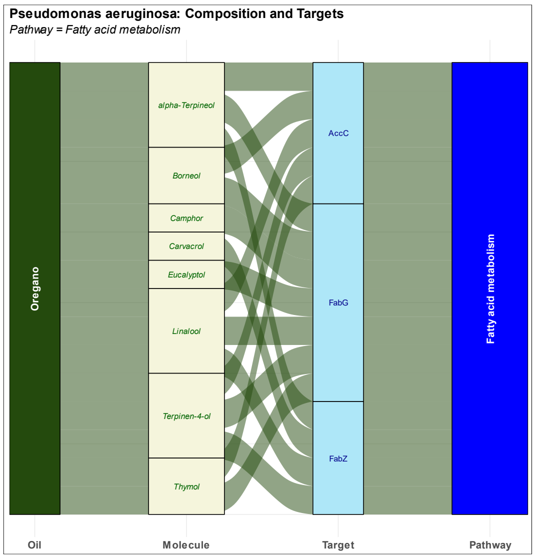

Microbial metabolism in diverse environments (https://www.kegg.jp/pathway/pae01120)

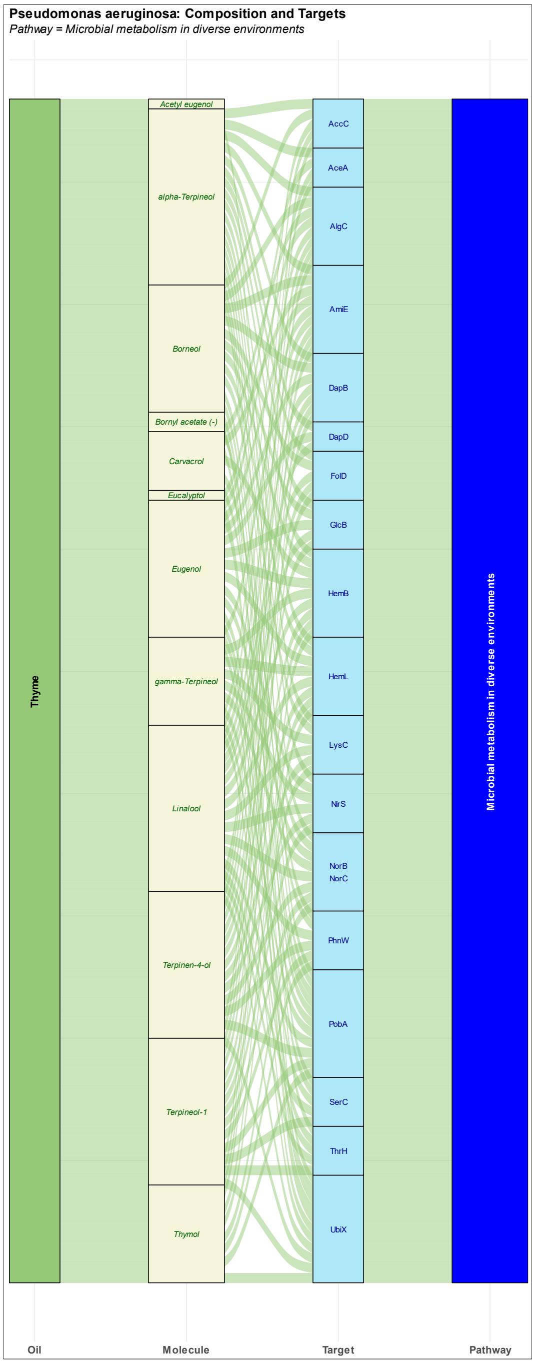

Phenylalanine, tyrosine and tryptophan biosynthesis (https://www.kegg.jp/pathway/pae00400)

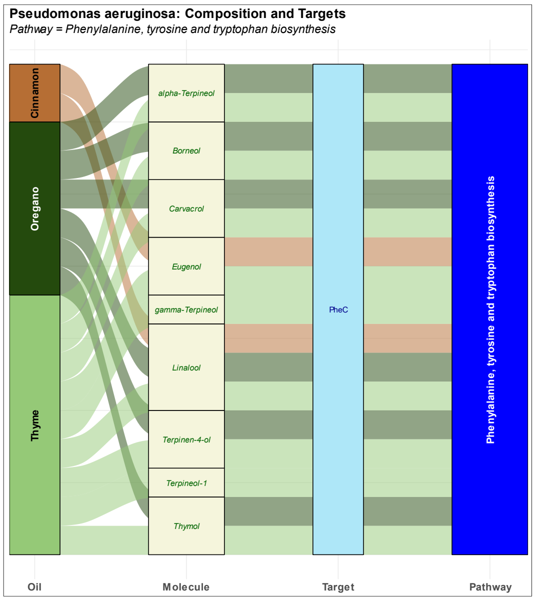

Riboflavin metabolism (https://www.kegg.jp/pathway/pae00740)

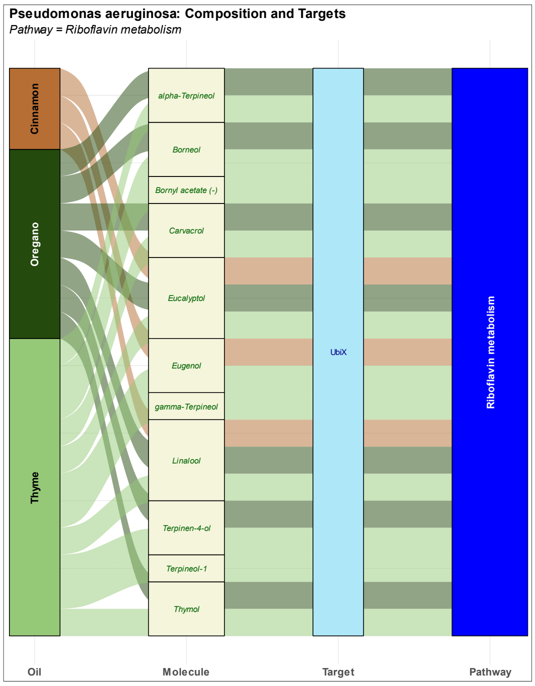

Terpenoid backbone biosynthesis (https://www.kegg.jp/pathway/pae00900)

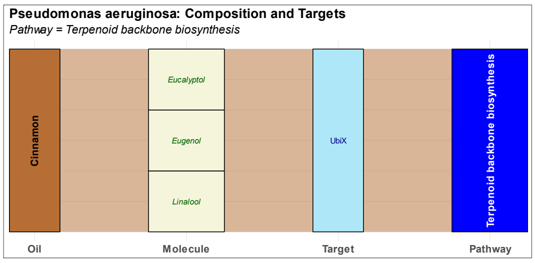

Section S12. Sequence of all the steps (scripts with input, output and description).

Step	Input and Output
**<u>Prepare Data</u>**<br><br>**Data mapping: UniProt <-> ATCC, UniProt <-> KEGG pathways, UniProt <-> Genes**	
**step01_prepare_data_atcc_mapping.R**	**INPUT:** CSV files from ATCC website<br>**OUTPUT:** atcc_bacteria_uniprot.RDS
**Description:** This script is used to create a file named "atcc_bacteria_uniprot.RDS" which is stored in the directory data/Mapping_data<br>NOTE: according to ATCC website, the download of CSV files is available only to "ATCC Genome Portal supporting members or those who have purchased a corresponding physical product". Therefore, in the official repository of the current project we cannot upload CSV files, but only the .RDS file produced by this script with the necessary columns for running the other scripts. However, we keep in the repository the script to create the file RDS from the set of CSV files.	
**step02a_read_kegg_data.R**<br>**step02b_check_kegg_data.R**	**INPUT:** data from KEGG website (via KEGGREST)<br>**OUTPUT(2a):** several files .RDS (for each org_code)<br>**OUTPUT(2b):** kegg_details.xlsx
**(needs to be run with several ATCC codes)**<br><br>**Description:** This script was used to extract KEGG data corresponding to different values of org_code (retrieved from https://www.kegg.jp/brite/br08611)<br>For some values of org_code, the script may fail with the following error message "Error in .getUrl(url, .flatFileParser) : Forbidden (HTTP 403).", because of the server access limitation for multiple queries. The problem was solved by adding Sys.sleep(x), with x=0.3<br>The script saves data into a .RDS file for each org_code.<br>NOTE: We cannot put in the github repository the RDS data, but only the script to create the file RDS by accessing the KEGG webserver through the R package KEGGREST keggList(org_code)<br>For each org_code, at the end of the script we report the success_rate, a percentage value which estimates how many objects have been retrieved, by running the command keggList(org_code).<br>In order to collect all the information from different .RDS files, use the additional script step02b_check_kegg_data.R (it produces an xlsx file, with the information - numer of uniprot, number of pathways, ...).	
**step03_prepare_data_kegg_mapping.R**	**INPUT:** several files .RDS (for each org_code)<br>**OUTPUT:** kegg_bacteria_uniprot.RDS
**Description:** This script creates a file named "kegg_bacteria_uniprot.RDS" which is stored in the directory data/Mapping_data and contains the information from all the .RDS files obtained for the different bacteria (previous point).	

<table>
<tr><th>Step</th><th>Input and Output</th></tr>
<tr><td>step04_prepare_data_genes_mapping.R</td><td>INPUT: data from UniProt website
OUTPUT: gene_uniprot.RDS</td></tr>
<tr><td colspan="2">Description: This script creates a file named "gene_uniprot.RDS". The mapping is limited to the UniProt entries which have a correspondence in the PDB archive. In fact, data are taken from the web (file pdbtosp.txt from UniProt web site)
https://ftp.uniprot.org/pub/databases/uniprot/current_release/knowledgebase/complete/docs/pdbtosp.txt
A data mining procedure elaborates the data by including only some methods: XRAY, NMR, EM, NEUTRON, IR, FIBER, OTHER. The data contains the mapping for all the organisms, not only those studied in the present work.</td></tr>
<tr><td colspan="2">Data from bacteria</td></tr>
<tr><td>step05_prepare_data_bacteria.R</td><td>INPUT: BACTERIADATA.xlsx (manually curated file)
OUTPUT: bacteria_data.RDS</td></tr>
<tr><td colspan="2">Description: This script creates a file named "bacteria_data.RDS" which contains info on bacteria, including codes on different databases (ATCC, KEGG) Input data is a manually curated file (BACTERIADATA.xlsx) starting from the five-letter organism codes extracted from the data frame previously obtained; information for data curation is obtained from UniProt.</td></tr>
<tr><td colspan="2">Data from composition</td></tr>
<tr><td>step06_prepare_data_composition.R</td><td>INPUT: OLI_COMPDATA.xlsx (EOs composition)
OUTPUT: composition.RDS, composition_new .RDS</td></tr>
<tr><td colspan="2">Description: This script creates a file named "composition.RDS" and another file names "composition_new .RDS". It extracts data about composition from an excel file.
There are columns related to the presence (as percentage) in the three oils, with the value 0.0333 we mean 'traces'. The file composition_new is in a wider format, with one column to define the numerical value and another column to define the oil; instead, the file composition has one column for each oil. In both files there is also the phytochemical classification. The two ways of presenting data are due to different uses on other scripts.</td></tr>
<tr><td colspan="2">Data from BioGPS calculations</td></tr>
<tr><td colspan="2">Run BioGPS commandline for the list of pockets and the database of molecules. For technical reasons, it can be convenient to split the job in several pieces.</td></tr>
</table>

Step	Input and Output
**step07_prepare_db_biogps.R**	**INPUT:** data from BioGPS (*.GS.txt files in the directory Biogps_Screening_data) and data from the web (biomaRt, Reactome, Uniprot)<br>**OUTPUT:** biogps.db (SQLite database)
**Description:** The script first creates an empty database and then populates with some data for the data linked to the pockets (pdb, uniprot, genes, pathways).	
**step08_prepare_data_biogps.R**	**INPUT:** biogps.db (SQLite database)<br>**OUTPUT:** biogps_data.RDS
**Description:** The script reads data from the database (zzscores_molpoc, zscores_mol and zscores_poc) and saves an object in the format .RDS to be used in the analysis.<br>The database containing the output of BioGPS from the two steps abovescan be found at https://figshare.com/ndownloader/files/57428944 andhas to be placed in the db folder.	
**<u>Check Data (optional)</u>**	
**step09_check_tablesummary.R**	**INPUT:** .RDS data from previous steps (kegg_bacteria_unified.RDS, atcc_bacteria_uniprot.RDS)<br>**OUTPUT:** finaltable2.csv
**Description:** Note that for the part related to biogps the script need access to the pocketome database (kindly provided by Molecular Discovery Ltd). However, this is not necessary for the analysis.	
**<u>Analysis: Target fishing and network analysis</u>**	
**step10_targetfishing_analysis.R**	**INPUT:** biogps_data.RDS, bacteria_data.RDS, composition_new.RDS<br>**OUTPUT:** Results_targetfishing.xlsx
**Description:** Data are read from the following .RDS files: biogps_data.RDS bacteria_data.RDS composition_new.RDS<br>Output consists in a series of tabular data sets, including network centrality, saved in the file Results_targetfishing.xlsx (in the directory output/target_fishing).	

Step	Input and Output
**<u>Results: barplots and heatmaps</u>**	
**step11_targetfishing_barplots_and_heatmaps.R**	**INPUT:** composition_new.RDS, manual_curation.xlsx, Results_targetfishing.xlsx,<br>**OUTPUT:** barplot_mol_nrtar_majorbacteria.svg, barplot_mol_nrtar_minorbacteria.svg, heatmaps_*_best20.svg (one for each bacterium)

Description: Data are read from the files composition_new.RDS and Results_targetfishing.xlsx
Additional input data is in the file manual_curation.xlsx (available in the directory data/Curation_data). Several sheets are present, including: multiple_genes, proteins (correct characters -uppercase/lowercase- given that all the genesymbols are stored in the databsase as upercase), target_curation, pdb_curation, pubmed, pocket_curation. The sheet Non_ecoli reports data that were removed (originally present for some errors), likely because E. coli is used as expression system. In the sheet multiple_genes, for each bacterium we report genes as groups whenever they correspond to the same complex or should be considered as duplicates for any other reason. Some genes from the analysis that have this peculiarity are the following:
Staphylococcus aureus: ACCA/ACCD
Pseudomonas aeruginosa: AMIC/AMIR, PQSB/PQSC, GSPI/GSPJ/GSPK, NORB/NORC
Escherichia coli: MCBA/MCBB/MCBC/MCBD, CARA/CARB, NARG/NARH/NARI
In the script these are then considered as the same genegroup and the higher centrality value along the genegroup is assigned. The script also produces the two barplots (for major and minor bacteria) and heatmaps for the major bacteria.
Data are read from the XLSX file of the previous step. First, data are considered by focusing on molecules; the question is: How many bacterial targets does each molecule hit?
Second, data are considered by focusing on phytocomplexes; the question is: How do the phytocomplexes interact with each target (through which molecules)?
We calculate a contribution for each target, by considering the zzscore and the percentage (composition). Then, we calculate the sum of contributions over all the molecules of phytocomplexes, and add the data about centrality.
We store the data of zzscore from molecule-target of oil-bacterium pairs and then extract the top n genes for each bacteria/oil pair (can adjust this value).
After having created a unique list of genes per bacteria (i.e., each bacteria's top n genes) we use these shortlist of genes (in dataframe format) to filter the previous data.
In order to get a shared list among the bacteria, we calculate the sum of `sum_contr` over the three oils for each gene; this will be used to rank genes in the Y axis of the heatmaps and scatterplot. We finally merge the ranked genes back with the filtered dataset (which is now ranked). In order to solve the problem of genes that should be considered as a group, we create a new data frame in which we add a new column, that is named 'gene'. This column is the name of grouped genes (whenever available), otherwise it is the old gene-symbol name. Then we use this (column gene) with bacteria and oil_eng to group rows and take only one row for group (with highest centrality).

Step	Input and Output
**<u>Results: GSEA</u>**	
**step12_gsea_analysis.R**	**INPUT:** bacteria_data.RDS, biogps_data.RDS, kegg_bacteria_unified.RDS, composition_new.RDS, manual_curation.xlsx<br>**OUTPUT:** Results_pathwaysenrichment_gsea.xlsx, all_pathways.RDS, sign_pathways.RDS, molecules_interaction.RDS
**Description:** In this step we perform pathway enrichment analysis focusing on pathways, molecules, bacteria, and oils. Input data are read from various .RDS files, filtered and processed in order to conduct gene set enrichment analyses (GSEA) for different bacteria-oil pairs across multiple pathways. The script identifies gene clusters linked to specific pathways, computes contribution scores, and filters pathways based on significance criteria like p-value and pathway size. It then generates a comprehensive heatmap visualizing pathway enrichments across bacteria and oils, using color intensities to represent p-values and annotations for gene interactions. Finally, it exports the results—including enriched pathways, gene-pathway associations, molecular contributions, and interaction details—into .xlsx and .RDS files for further analysis.	
**step13_gsea_alluvialplots.R**	**INPUT:** sign_pathways.RDS, molecules_interaction.RDS<br>**OUTPUT:**.SVG files, one for each pathway
**Description:** This last R script generates alluvial (flow) plots illustrating the relationships among bacteria, pathways, molecules, genes, and oils based on pathway enrichment data stored in specific .RDS files.<br>It reads data from an .xlsx file containing pathways and interaction details, then processes each pathway-bacteria pair iteratively. For each pair, it extracts relevant targets with strong interactions, it curates pathway names for clarity (splitting long names into two lines), and it assigns colors to oils. The script constructs layered data structures to represent different object categories (oils, molecules, genes, pathways), assigns visual styles based on category, and creates alluvial plots using *ggplot2* and *ggalluvial*, with customized text styles and color schemes. Each plot visualizes the flow of relationships from oils through molecules and genes to pathways, and is saved as an SVG file for further visualization.	
